# Supplementary material for: Temporal refinement of Dach1 expression contributes to the development of somatosensory neurons
Source: EMBO J. 2025 Apr 9;44(10):2882–905. doi: 10.1038/s44318-025-00427-y (PMC12084601; doi:10.1038/s44318-025-00427-y)
Supplement: Supplementary file 1 — Appendix [file 44318_2025_427_MOESM1_ESM.pdf]

Appendix for:
Temporal refinement of Dach1 expression contributes to the development of somatosensory neurons

This file includes:

Appendix figures and figure legends S1-5:

- **Appendix Figure S1 (Pages 2-3)**
- **Appendix Figure S2 (Pages 4-6)**
- **Appendix Figure S3 (Pages 7-9)**
- **Appendix Figure S4 (Pages 10-11)**
- **Appendix Figure S5 (Pages 12-13)**

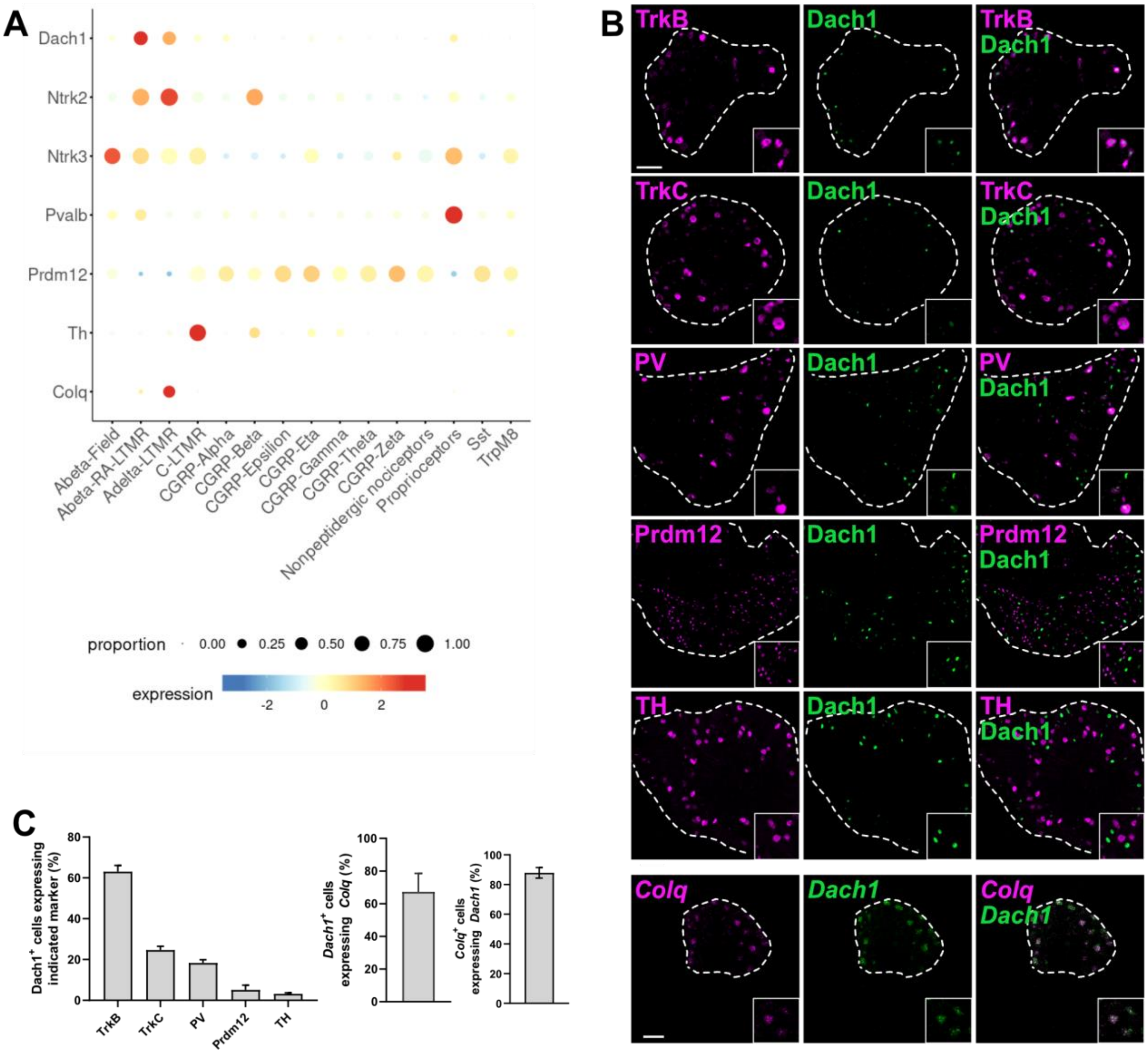
A
Dach1
Ntrk2
Ntrk3
Pvalb
Prdm12
Th
Colq
Abeta-Field
Abeta-RA-LTMR
Adelta-LTMR
C-LTMR
CGRP-Alpha
CGRP-Beta
CGRP-Epsilon
CGRP-Eta
CGRP-Gamma
CGRP-Theta
CGRP-Zeta
Nonpeptidergic nociceptors
Proprioceptors
Sst
TrpM8
proportion 0.00 0.25 0.50 0.75 1.00
expression -2 0 2
B
TrkB
Dach1
TrkC
PV
Prdm12
TH
Colq
C
Dach1+ cells expressing indicated marker (%)
TrkB
TrkC
PV
Prdm12
TH
Dach1+ cells expressing Colq (%)
Colq+ cells expressing Dach1 (%)

Appendix Figure S1. Dach1 is predominantly retained in TrkB^{+} somatosensory neurons at adulthood.

(A) Bubble plot of *Dach1* and selected genes highlighting *Dach1* enriched expression in Aβ-RA-LTMR and Aδ-LTMR in adult somatosensory neurons (generated using https://ernforsgroup.shinyapps.io/MouseDRGNeurons/).

(B) Double immunohistochemistry targeting Dach1 and indicated somatosensory subtype markers performed on transverse sections of thoracic dorsal root ganglia harvested from adult wild-type mice. Co-staining of *Colq* and *Dach1* was performed by fluorescent *in situ* hybridization (RNAScope). Scale bar, 100 µm.

(C) Percentage of colocalization of Dach1 and indicated neuronal markers (N= 2, mean ± SEM)

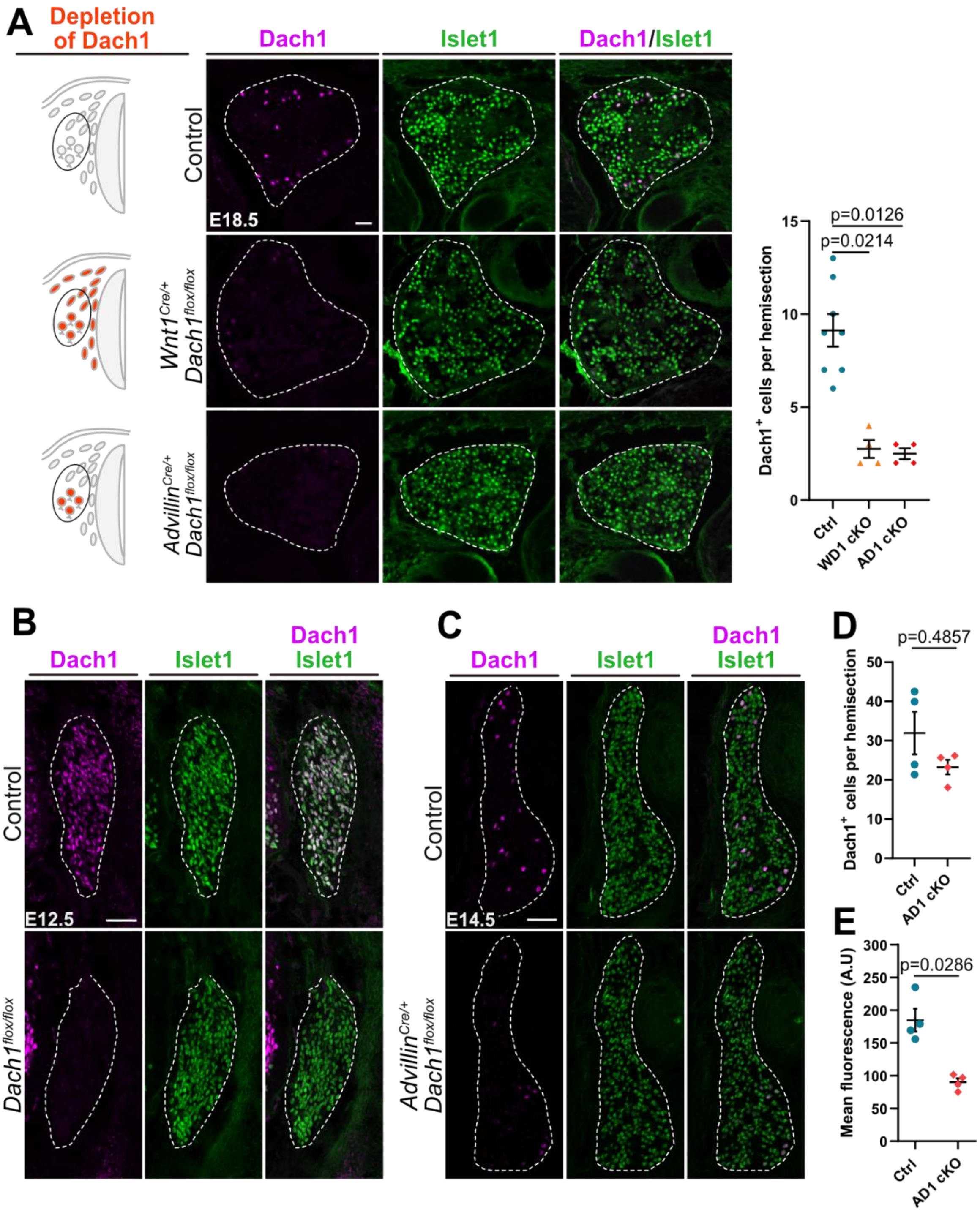

A
Depletion of Dach1
Dach1
Islet1
Dach1/Islet1
Control
E18.5
Wnt1Cre/+ Dach1flox/flox
AdvillinCre/+ Dach1flox/flox
p=0.0126
p=0.0214
Dach1+ cells per hemisection
15
10
5
0
Ctrl
WD1 cKO
AD1 cKO
B
Dach1
Islet1
Dach1 Islet1
Control
E12.5
Wnt1Cre/+ Dach1flox/flox
C
Dach1
Islet1
Dach1 Islet1
Control
E14.5
AdvillinCre/+ Dach1flox/flox
D
p=0.4857
Dach1+ cells per hemisection
50
40
30
20
10
0
Ctrl
AD1 cKO
E
p=0.0286
Mean fluorescence (A.U)
300
250
200
150
100
50
0
Ctrl
AD1 cKO

Appendix Figure S2. Validation of mouse line strategies to conditionally invalidate Dach1.

(A) Left, schematic representation of the two strategies established to deplete Dach1 from the neural crest or from post-mitotic somatosensory neurons. Cells expected to be targeted by each startegy are labelled in orange. Middle, Representative pictures of double immunostainings targeting Dach1 and the pan-sensory neuron marker Islet1 performed on thoracic transverse DRG sections of control, $\textit{Wnt1}^{Cre/+}\textit{;Dach1}^{flox/flox}$ (*WD1* cKO) and $\textit{Advillin}^{Cre/+}\textit{;Dach1}^{flox/flox}$ (*AD1* cKO) E18.5 embryos. Right, quantification analysis represented as scatter dot plot comparing the mean number of neurons immunostained for Dach1 detected in dorsal root ganglia on transverse hemisections of indicated genotypes. Each dot in this scatter plot indicates the mean value obtained for a single embryo. Graphical data in this panel and in the subsequent ones are presented as mean ± SEM, each dot in scatter plots representing a biological replicate. Kruskal-Wallis test with Dunn's post hoc multiple comparison.

(**B**) Representative pictures of double immunostainings targeting Dach1 and the pan-sensory neuron marker Islet1 performed on thoracic transverse DRG sections of control and $\textit{Wnt1}^{Cre/+}\textit{;Dach1}^{flox/flox}$ (*WD1* cKO) E12.5 embryos. Note that Dach1 is already completely abolished at this stage in the *WD1* cKO line.

(**C**) Representative pictures of double immunostainings targeting Dach1 and the pan-sensory neuron marker Islet1 performed on thoracic transverse DRG sections of control and $\textit{Advillin1}^{Cre/+}\textit{;Dach1}^{flox/flox}$ (*AD1* cKO) E12.5 embryos. Note that Dach1 is still detected at this stage, depsite at a lower fluorescence level.

(**D**) Quantification of the mean number of $Dach1^{+}$ cells per DRG cells on hemisections of E14.5 control and *AD1* cKO embryos. Mann-Whitney test.

(**E**) Quantification of the mean fluorescence level of Dach1^{+} immunostaining (arbitrary unit, A.U.) in DRG neurons of E14.5 control and *AD1* cKO embryos. Mann-Whitney test.

Dorsal root ganglia are delineated by white dashed lines.

Scale bar, 50 μm.

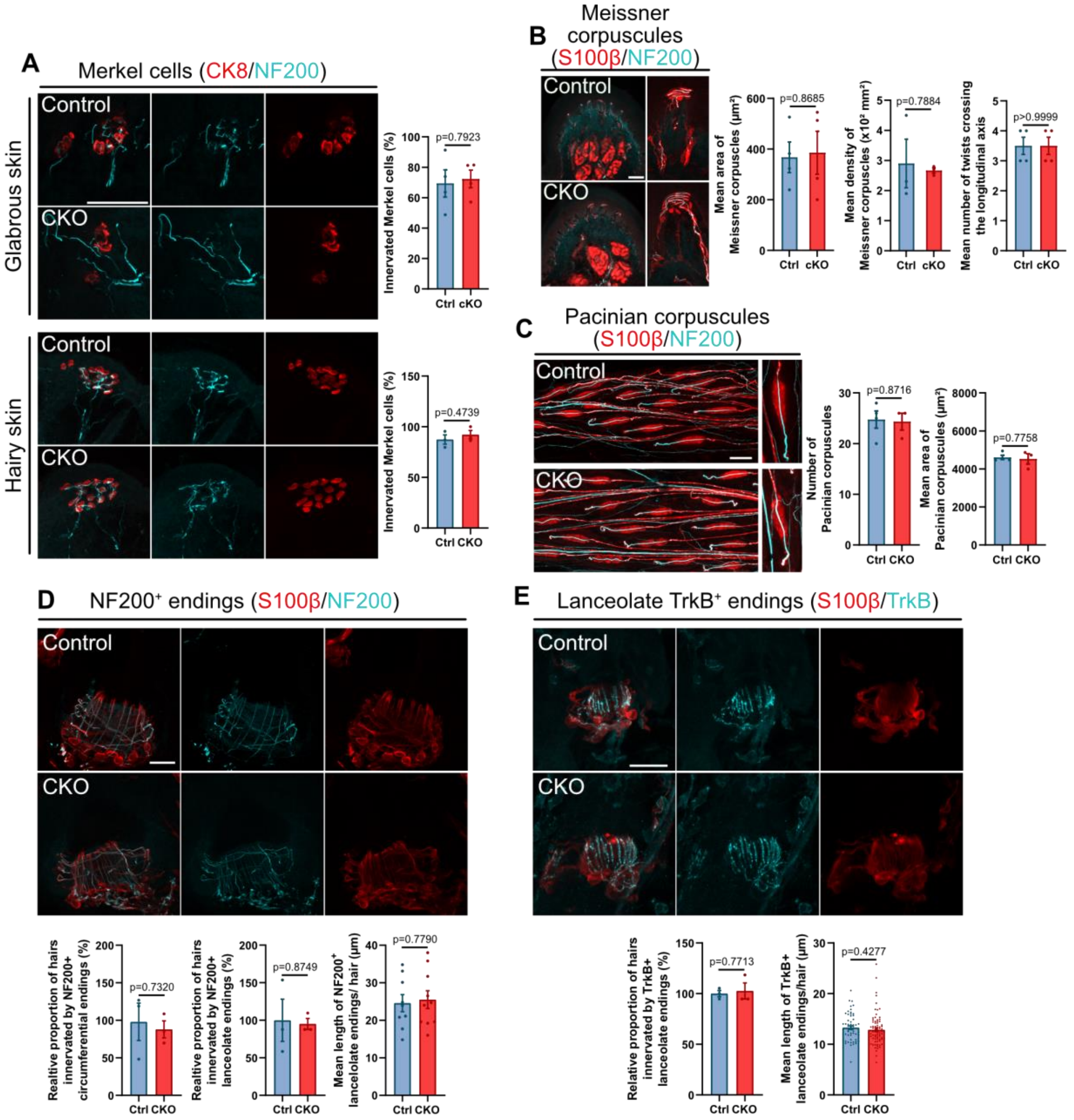
A
Merkel cells (CK8/NF200)
Glabrous skin
Hairy skin
Control
CKO
Innervated Merkel cells (%)
p=0.7923
p=0.4739
Ctrl cKO
Ctrl CKO
B
Meissner corpuscules (S100β/NF200)
Mean area of Meissner corpuscles (µm²)
p=0.8685
Mean density of Meissner corpuscles (x10² mm²)
p=0.7884
Mean number of twists crossing the longitudinal axis
p>0.9999
Ctrl cKO
C
Pacinian corpuscules (S100β/NF200)
Number of Pacinian corpuscules
p=0.8716
Mean area of Pacinian corpuscules (µm²)
p=0.7758
Ctrl CKO
D
NF200⁺ endings (S100β/NF200)
Realtive proportion of hairs innervated by NF200+ circumferential endings (%)
p=0.7320
Realtive proportion of hairs innervated by NF200+ lanceolate endings (%)
p=0.8749
Mean length of NF200⁺ lanceolate endings/ hair (µm)
p=0.7790
Ctrl CKO
E
Lanceolate TrkB⁺ endings (S100β/TrkB)
Relative proportion of hairs innervated by TrkB+ lanceolate endings (%)
p=0.7713
Mean length of TrkB+ lanceolate endings/hair (µm)
p=0.4277
Ctrl CKO

Appendix Figure S3. The loss of Dach1 does not affect the innervation and morphology of touch receptor end-organs.

(A) Left, Representative immunostaining images of Merkel cells and their innervating fibers in glabrous or hairy skin cross-sections collected from mice of indicated genotypes. Right, Quantification of the proportion of innervated Merkel cells. Mean ± SEM, Student's unpaired t tests. Each dot represents the mean percentage of innervated Merkel cells from a single individual. Scale bar, 50 μm.

(B) Left, Representative immunostaining images of Meissner corpuscules and their innervating fibers in glabrous skin cross-sections collected from mice of indicated genotypes. Right, Quantification of the area (left) and density (center) of Meissner corpuscules as well as the number of NF200+ fiber twists crossing the longitundinal axis (right) as a readout of their innervation complexity. Mean ± SEM, Student's unpaired t tests. Each dot represents the mean value obtained for the indicated parameters in a single individual. Scale bar, 100 μm.

(C) Left, Representative whole-mount immunostaining images of Ulnar Pacinian corpuscules and their innervating fibers from mice of indicated genotypes. Right, Quantification of the number (left) and mean area (right) of Ulnar Pacinian corpuscules. Mean ± SEM, Student's unpaired t tests. Each dot represents the mean value obtained for the indicated parameters in a single individual. Scale bar, 100 μm.

(D) Up, Representative immunostaining images of NF200+ Aβ circumferential and lanceolate endings innervating guard hair follicles and their S100β+ associated terminal Schwann cells. Bottom, Quantification of the relative proportion of guard hairs innervated by Aβ circumferential (left) or Aβ lanceolate endings (center) in *AD1* cKO mice compared to control. Each dot represents the mean value obtained for the indicated parameters in a single individual. Bottom right, Quantification of the mean length of Aβ lanceolate endings innervating a hair

follicle in mice of indicated genotypes. Each dot represents the mean lenght of Aβ lanceolate endings innervating a single guard hair follicle. Mean ± SEM, Student's unpaired t tests. Scale bar, 20 μm.

(E) Up, Representative immunostaining images of TrkB+ Aδ lanceolate endings innervating zigzag hair follicles and their S100β+ associated terminal Schwann cells. Bottom left, Quantification of the relative proportion of zigzag hairs innervated by Aδ longitudinal endings in *AD1* cKO mice compared to control. Each dot represents the mean value obtained for a single individual. Bottom right, Quantification of the mean length of Aδ lanceolate endings innervating a zigzag hair follicle in mice of indicated genotypes. Each dot represents the mean lenght calculated for a single hair follicle. Mean ± SEM, Student's unpaired t tests. Scale bar, 20 μm.

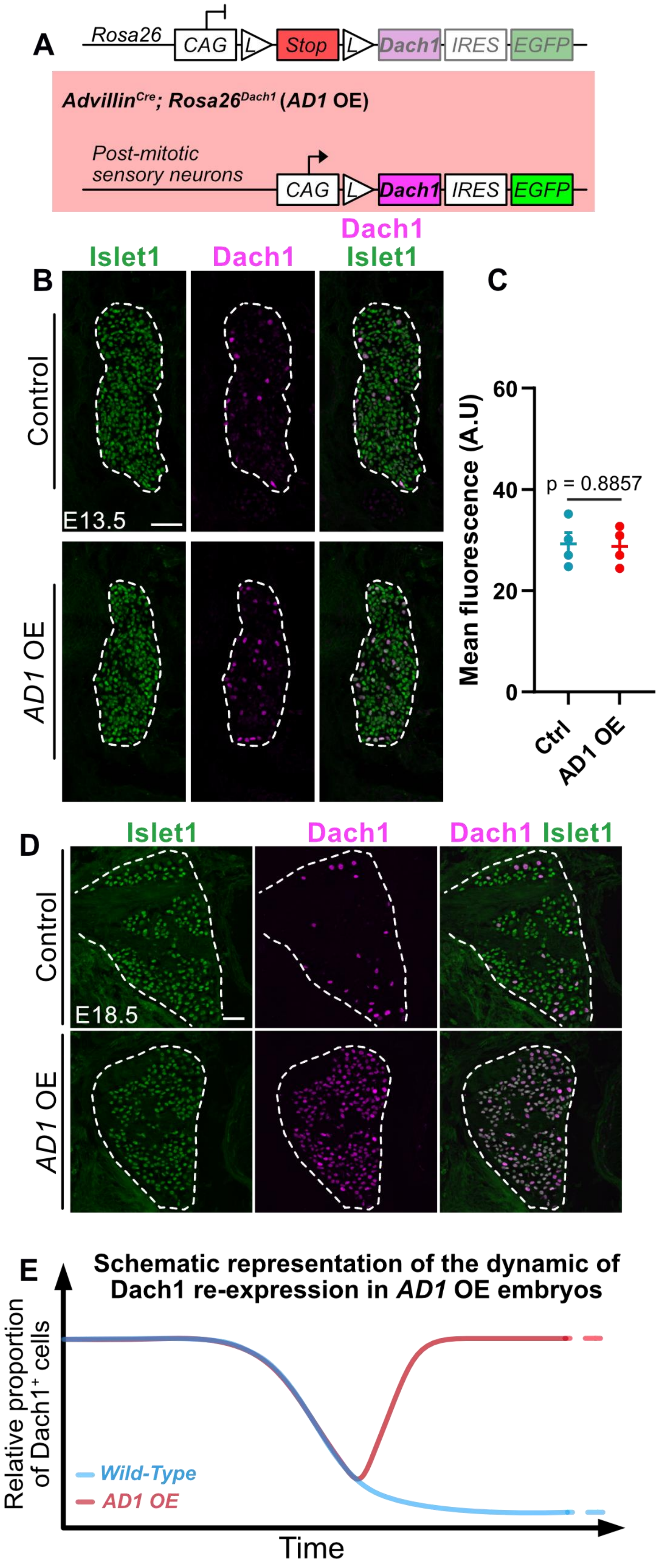
A
Rosa26
CAG
L
Stop
L
Dach1
IRES
EGFP
Advillin^Cre; Rosa26^Dach1 (AD1 OE)
Post-mitotic
sensory neurons
CAG
L
Dach1
IRES
EGFP
B
Islet1
Dach1
Dach1
Islet1
Control
E13.5
AD1 OE
C
Mean fluorescence (A.U)
60
40
20
0
p = 0.8857
Ctrl
AD1 OE
D
Islet1
Dach1
Dach1 Islet1
Control
E18.5
AD1 OE
E
Schematic representation of the dynamic of
Dach1 re-expression in AD1 OE embryos
Relative proportion
of Dach1+ cells
Wild-Type
AD1 OE
Time

Appendix Figure S4. Transgenic mouse line strategy to perturb the temporal refinement of Dach1 *in vivo.*

(A) Schematic representation of the strategy established to force late stage pan-neuronal expression of Dach1 in post-mitotic somatosensory neurons (red background). To make reading easier, embryos overexpressing *Dach1* through the *Advillin^{Cre} and Rosa26^{Dach1}* alleles are abbreviated *AD1* OE.

(B) Representative pictures of double immunostainings targeting Dach1 and the pan-sensory neuron marker Islet1 performed on thoracic transverse DRG sections of control and *AD1* OE E13.5 embryos. Scale bar, 50 µm.

(C) Quantification of the mean fluorescence level of Dach1 immunostaining (arbitrary unit, A.U.) among Islet1^{+} DRG neurons of E13.5 control and *AD1* OE embryos. Each dot represents the mean value obtained for a single individual. Mean ± SEM, Mann-Whitney test.

(D) Representative pictures of double immunostainings targeting Dach1 and the pan-sensory neuron marker Islet1 performed on thoracic transverse DRG sections of control and *AD1* OE E18.5 embryos. Note that the picture depicting Islet1 staining in the *AD1* OE condition is also used to illustrate data in Figure 5A.

Scale bar, 50 µm.

(E) Schematic representation of the characteristic features of the startegy established to counter the temporal refinement of Dach1. The blue line summarizes the dynamic of Dach1 broad-to-restricted expression observed in wild-type embryos. The red line recapitulates the results observed in the *AD1* OE embryos, namely that *Dach1* temporal restriction is initially properly triggered at E13.5 before being broadly expressed again at later stage.

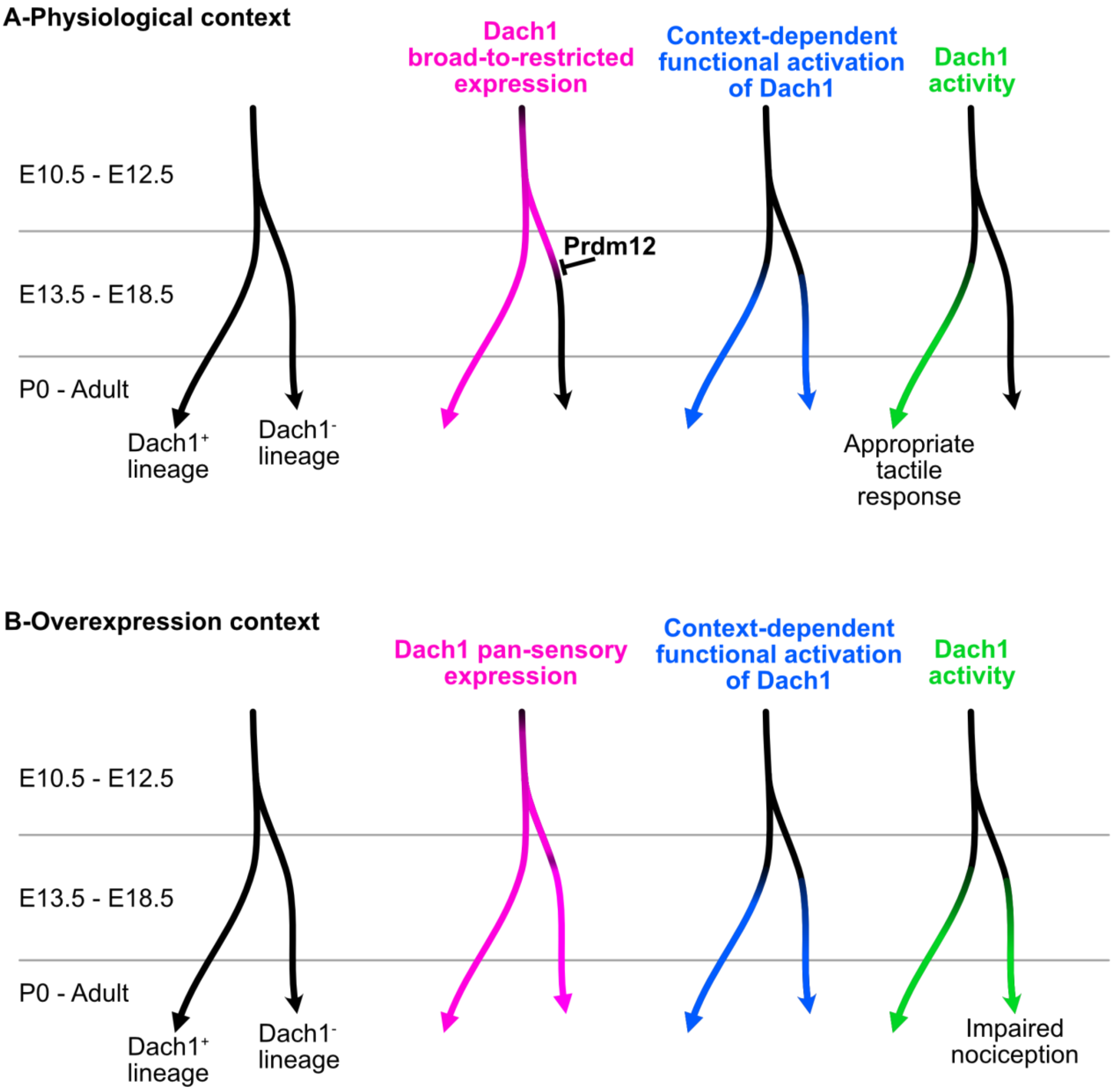
A-Physiological context
Dach1 broad-to-restricted expression
Context-dependent functional activation of Dach1
Dach1 activity
E10.5 - E12.5
E13.5 - E18.5
P0 - Adult
Prdm12
Dach1+ lineage
Dach1- lineage
Appropriate tactile response
B-Overexpression context
Dach1 pan-sensory expression
Context-dependent functional activation of Dach1
Dach1 activity
E10.5 - E12.5
E13.5 - E18.5
P0 - Adult
Dach1+ lineage
Dach1- lineage
Impaired nociception

Appendix Figure S5. Model of Dach1 refinement and functional activity. Dach1 is expressed following a broad-to-restricted expression dynamic, being first expressed in all post-mitotic developing somatosensory neurons before seggregating into a branch representing neuronal subtypes maintaining Dach1 expression and another branch representing neuronal subtypes repressing Dach1, in part through the activity of Prdm12 (A-pink path). This change timely occurs before a contextual developmental switch driving the functional activation of Dach1 (A-Blue path). The combination of its timely refined expression and triggering of its functional competence results in an appropriate subtype-restricted Dach1 activity required for appropriate tactile response (A-Green path). The forced maintenance of Dach1 in the putative Dach1^{-} lineage (B-Pink path) associated with the triggering of its functional activation (B-Blue path) consequently results in Dach1 becoming functional in the putative Dach1^{-} lineage, ultimately resulting in impaired nociception (B-Green path).
