# Supplementary figures and images for: Temporal refinement of Dach1 expression contributes to the development of somatosensory neurons
Source: EMBO J. 2025 Apr 9;44(10):2882–905. doi: 10.1038/s44318-025-00427-y (PMC12084601; doi:10.1038/s44318-025-00427-y)

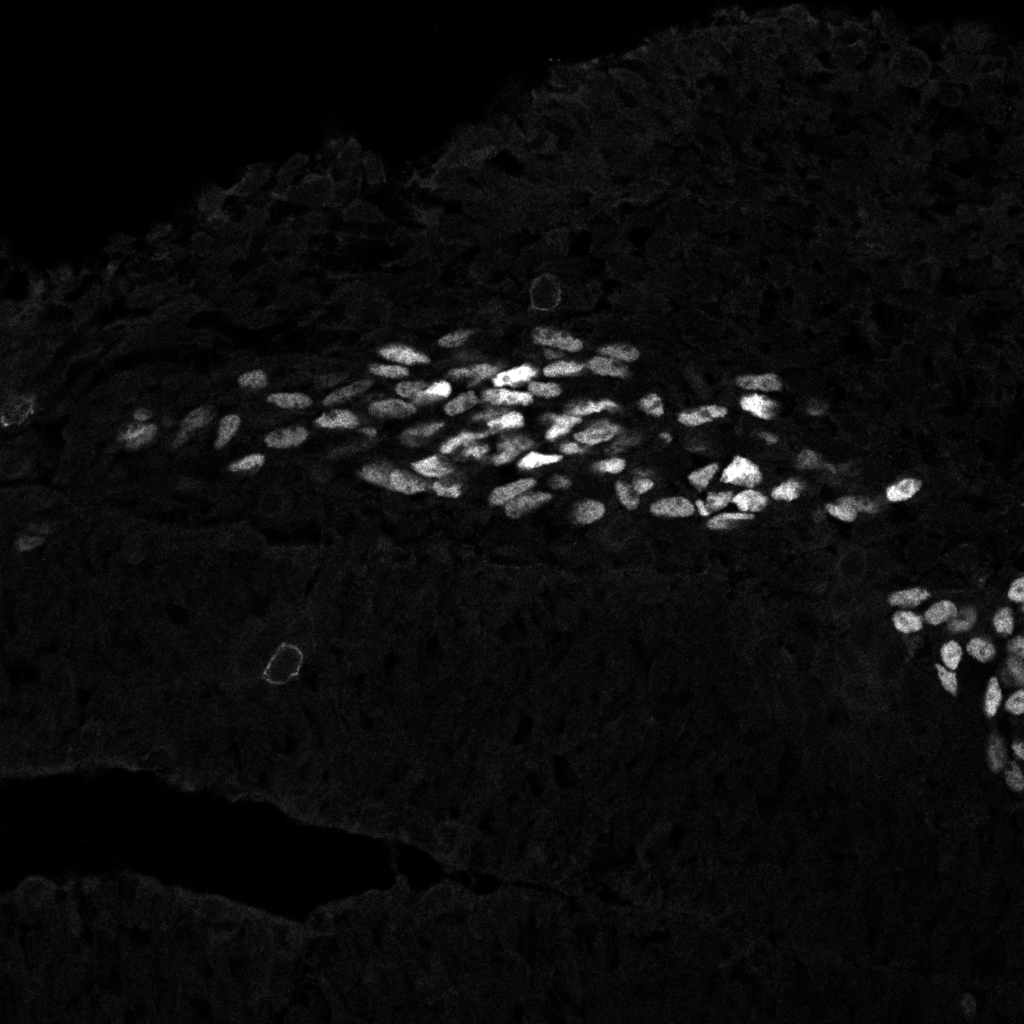

Supplement: Supplementary file 6 — Source data Fig. 1 [file 44318_2025_427_MOESM6_ESM.zip › Figure 1/1A/WT1 E10.5 Dach1 green Islet1 red 40x 008.tif]

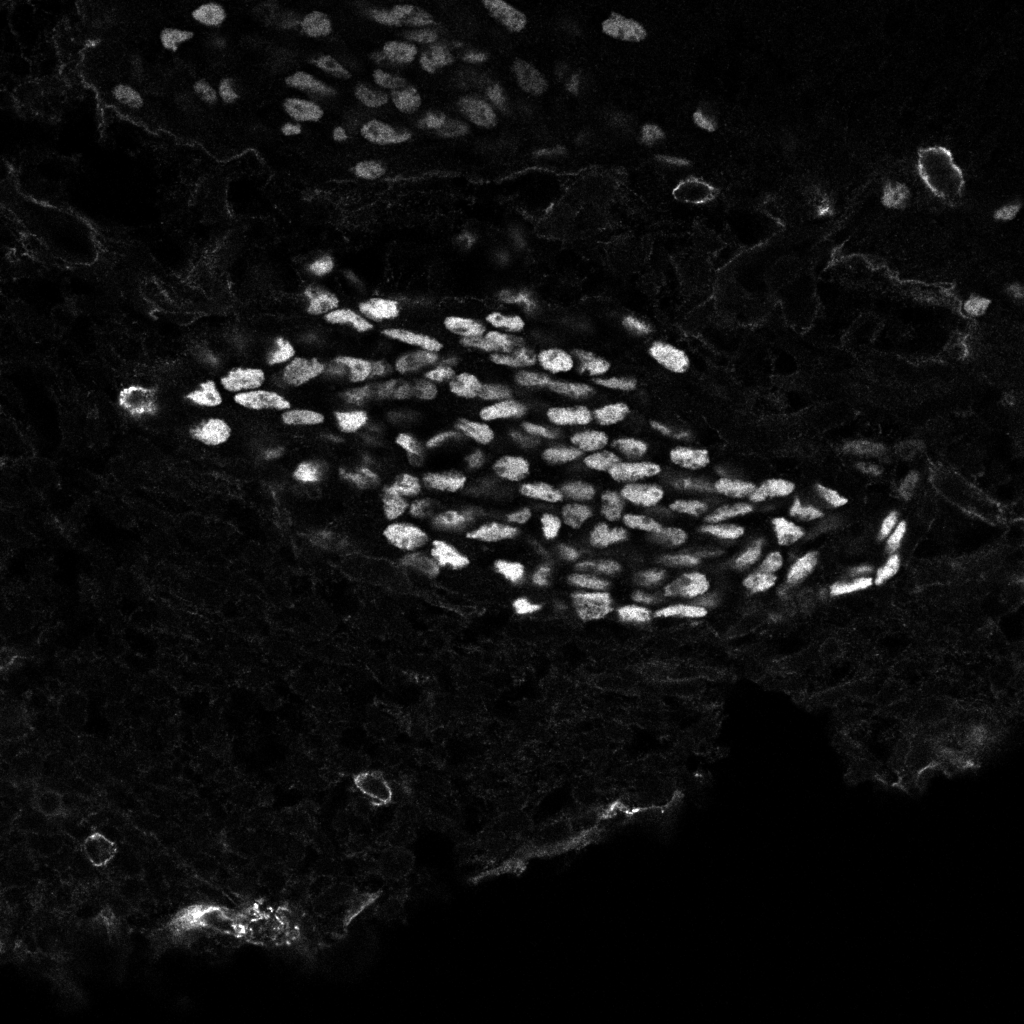

Supplement: Supplementary file 6 — Source data Fig. 1 [file 44318_2025_427_MOESM6_ESM.zip › Figure 1/1A/WT1 E11.5 Dach1 green Islet1 red 40x 009.tif]

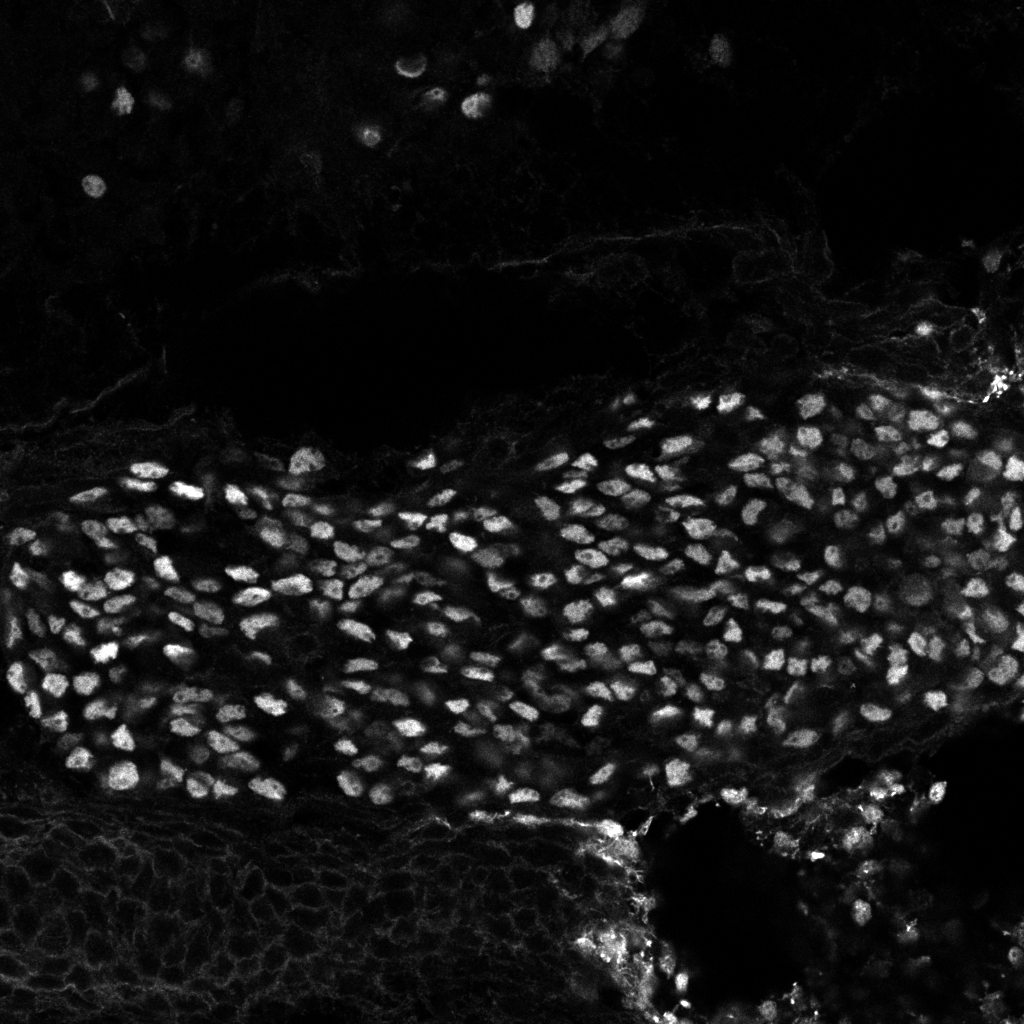

Supplement: Supplementary file 6 — Source data Fig. 1 [file 44318_2025_427_MOESM6_ESM.zip › Figure 1/1A/WT1 E12.5 Dach1 green Islet1 red 40x 001.tif]

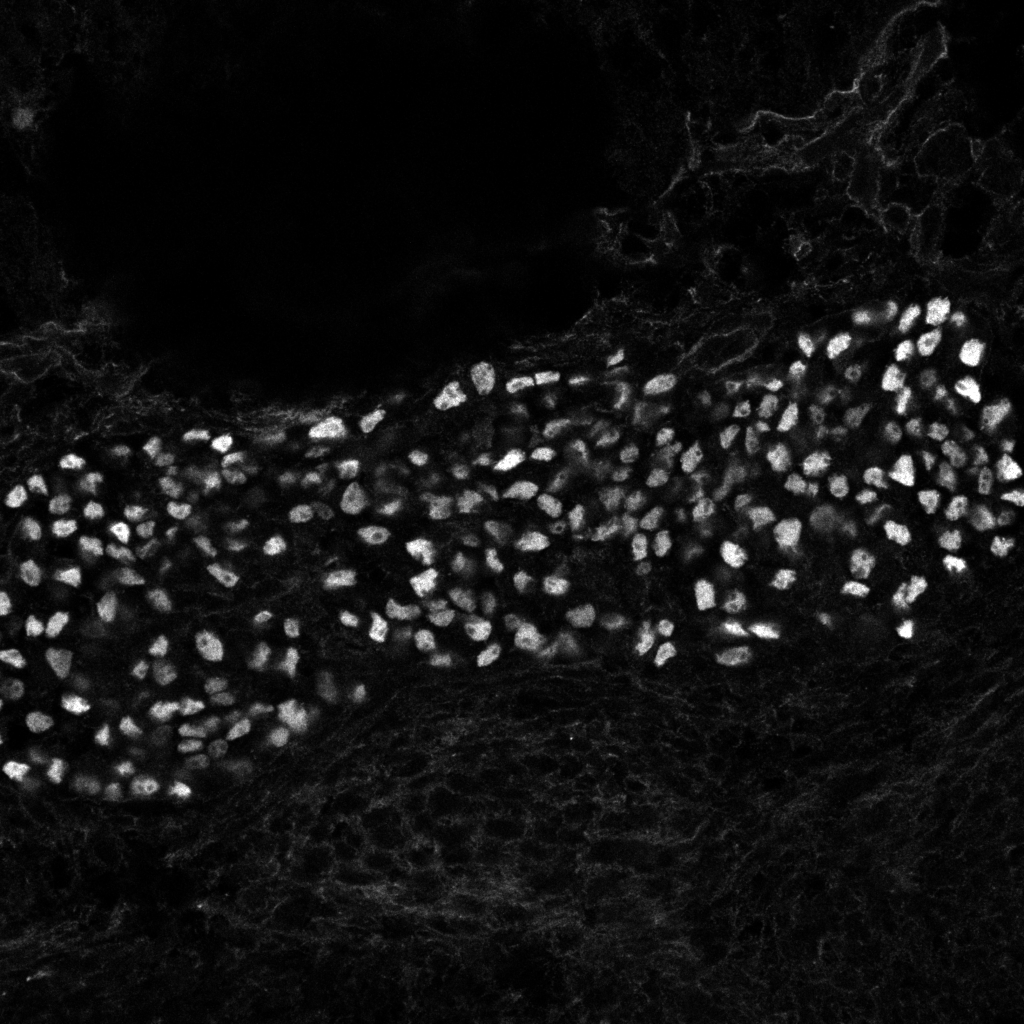

Supplement: Supplementary file 6 — Source data Fig. 1 [file 44318_2025_427_MOESM6_ESM.zip › Figure 1/1A/WT2 E13.5 Dach1 green Islet1 red 40x 001.tif]

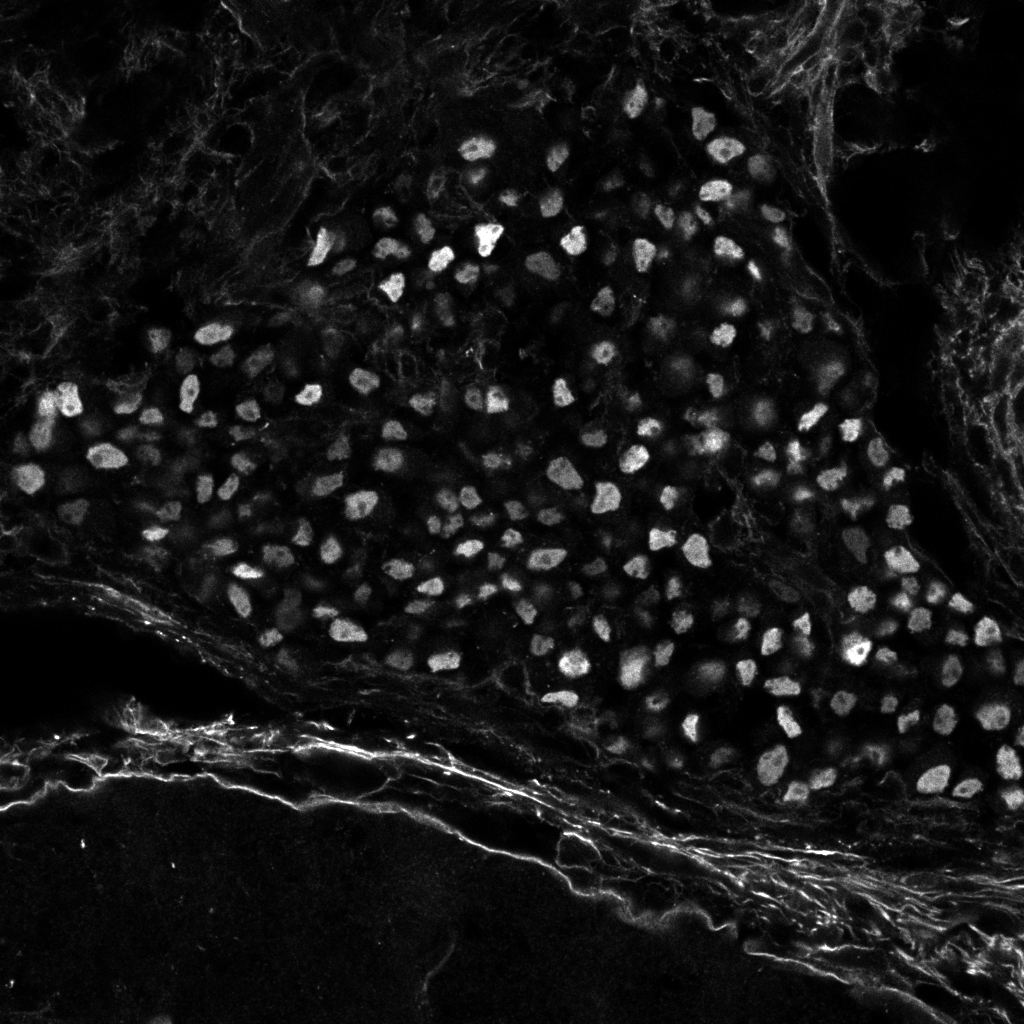

Supplement: Supplementary file 6 — Source data Fig. 1 [file 44318_2025_427_MOESM6_ESM.zip › Figure 1/1A/WT2 E14.5 Dach1 green Islet1 red 40x 001.tif]

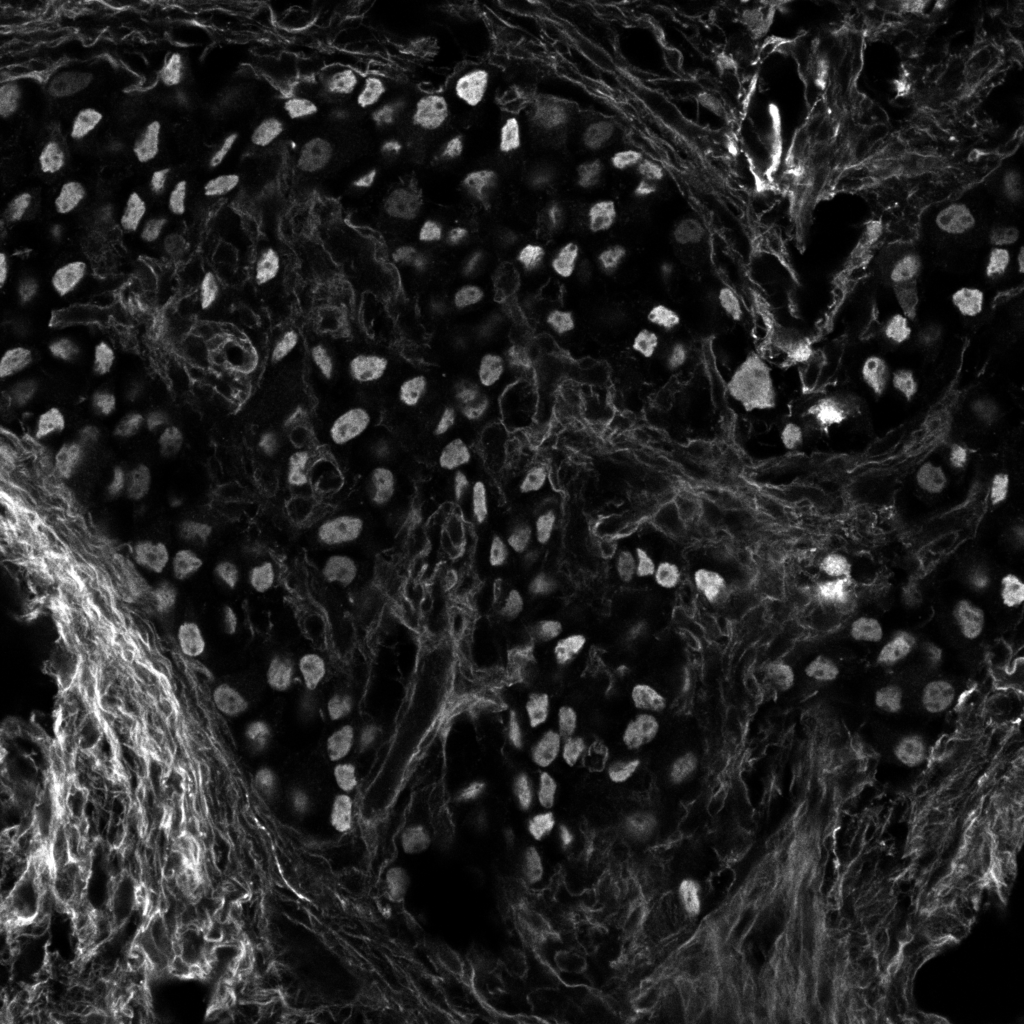

Supplement: Supplementary file 6 — Source data Fig. 1 [file 44318_2025_427_MOESM6_ESM.zip › Figure 1/1A/WT2 E16.5 Dach1 green Islet1 red 40x 001.tif]

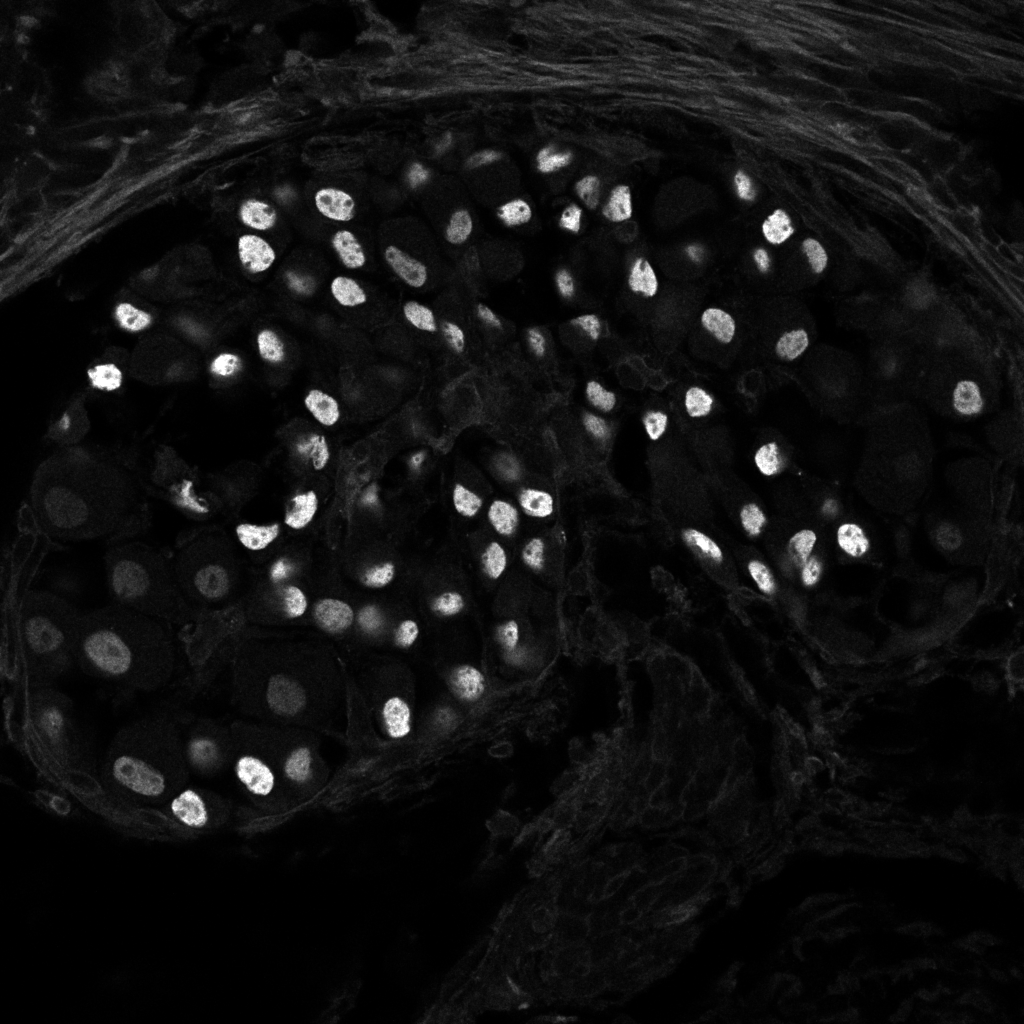

Supplement: Supplementary file 6 — Source data Fig. 1 [file 44318_2025_427_MOESM6_ESM.zip › Figure 1/1A/WT2 E18.5 Dach1 green Islet1 red 40x 001.tif]

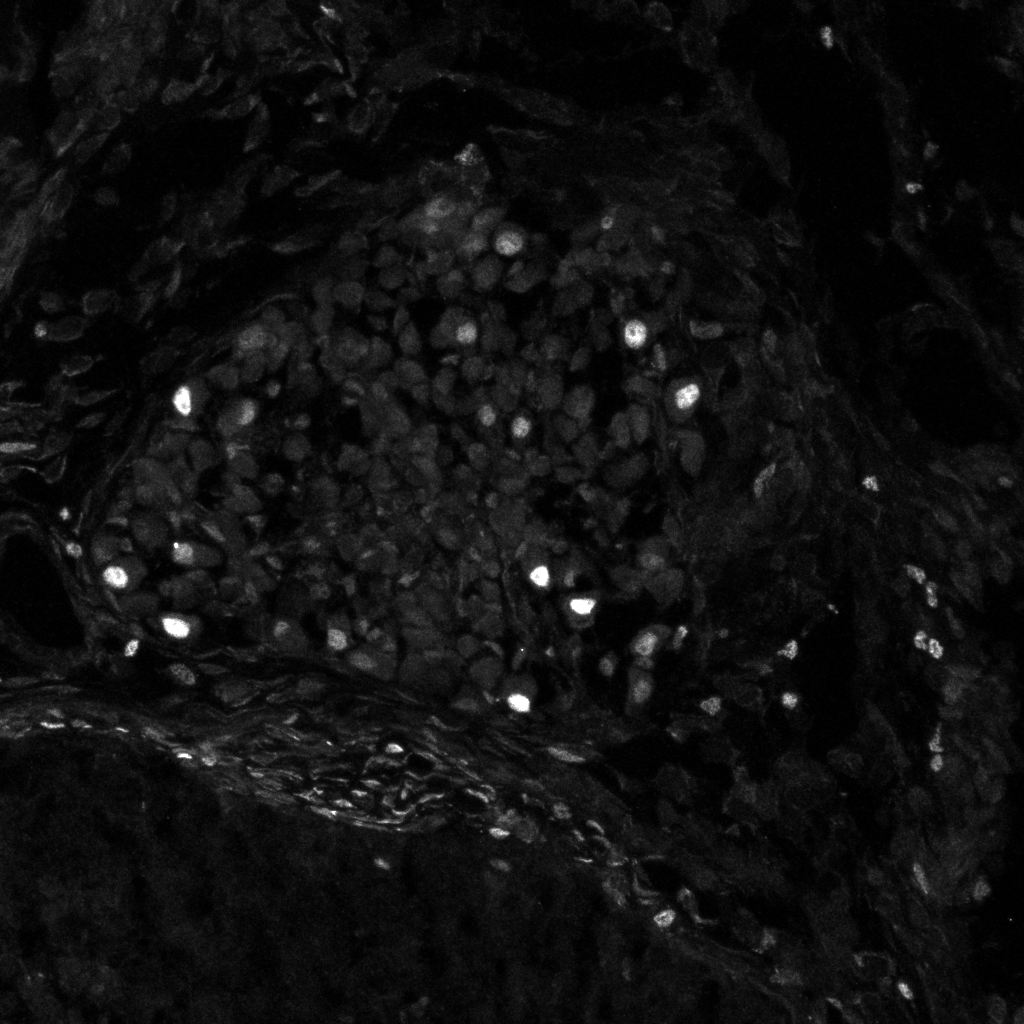

Supplement: Supplementary file 6 — Source data Fig. 1 [file 44318_2025_427_MOESM6_ESM.zip › Figure 1/1E/WT E18.5 Dach1 green c-Maf red 20X 005.tif]

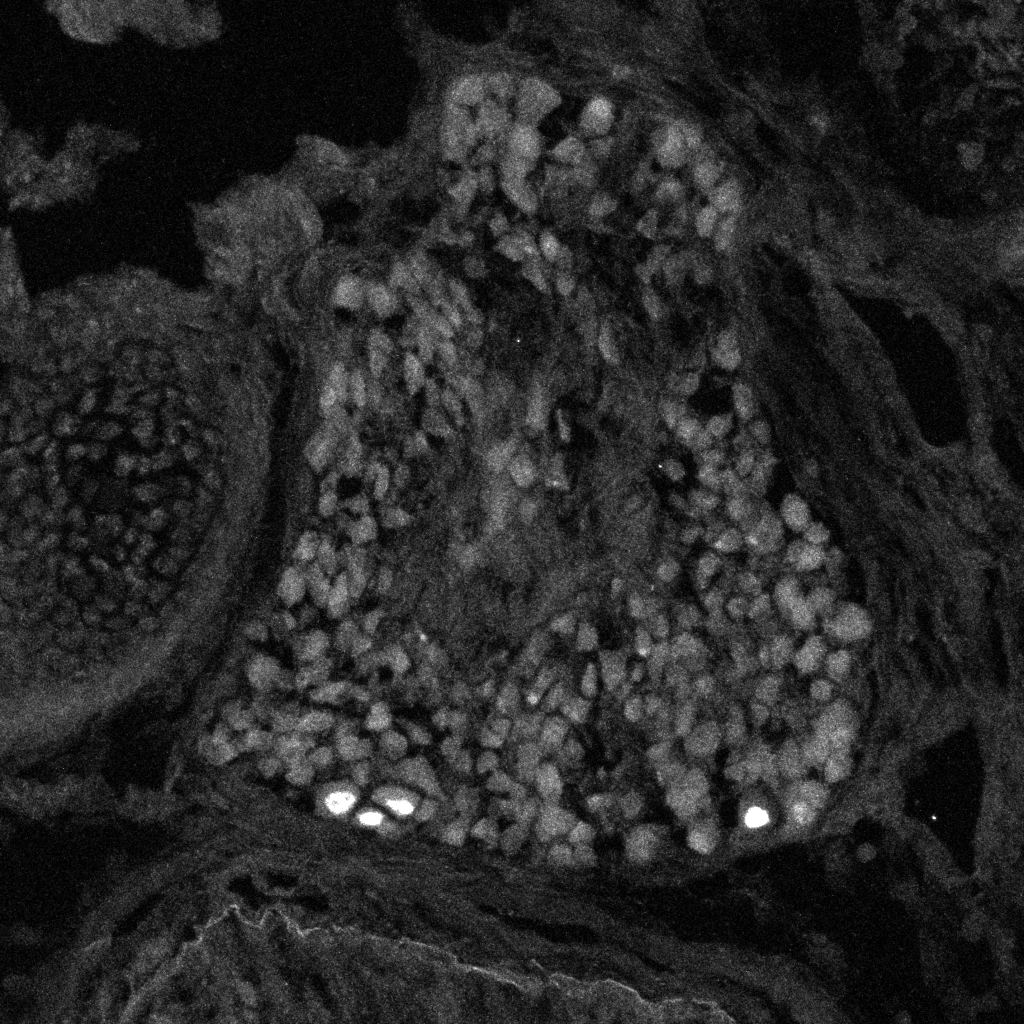

Supplement: Supplementary file 6 — Source data Fig. 1 [file 44318_2025_427_MOESM6_ESM.zip › Figure 1/1E/WT E18.5 Dach1 green MafA red 20X 003.tif]

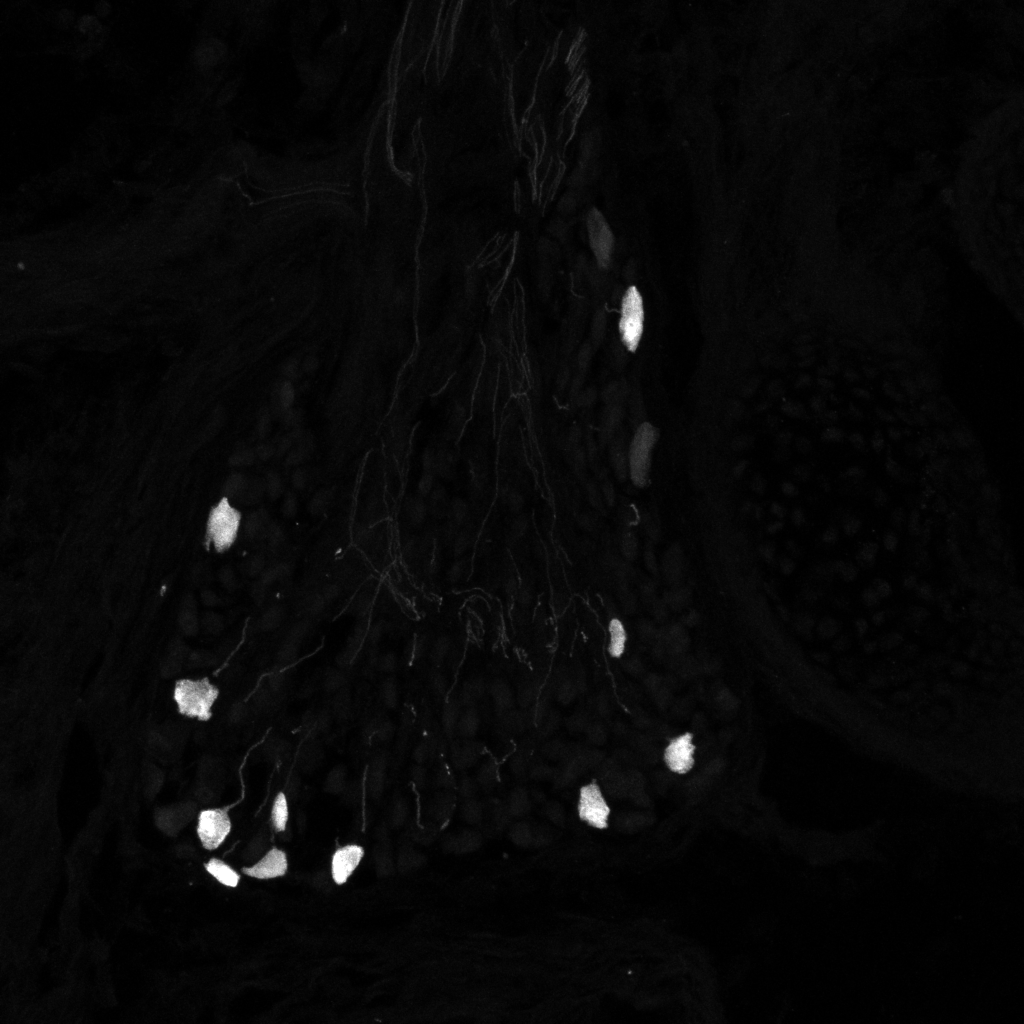

Supplement: Supplementary file 6 — Source data Fig. 1 [file 44318_2025_427_MOESM6_ESM.zip › Figure 1/1E/WT E18.5 Dach1 green Parvalbumin red.tif]

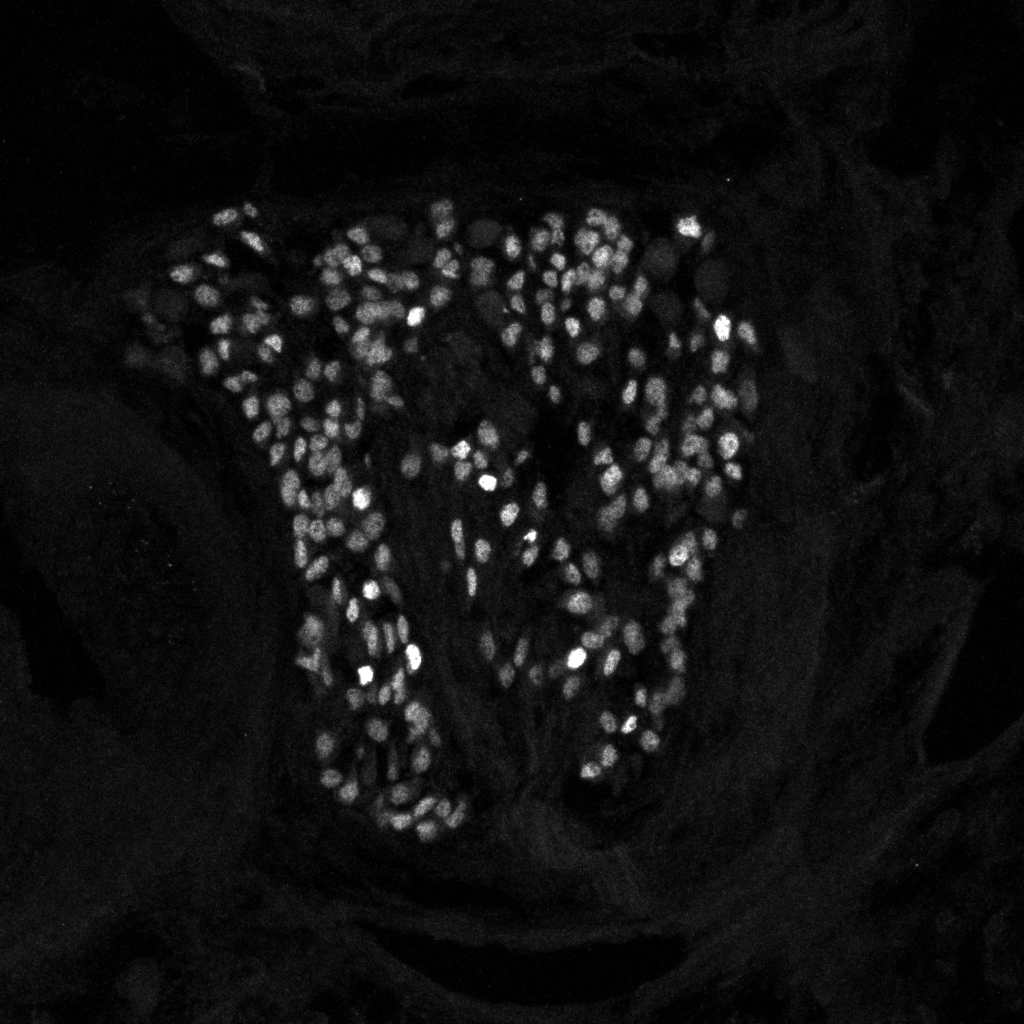

Supplement: Supplementary file 6 — Source data Fig. 1 [file 44318_2025_427_MOESM6_ESM.zip › Figure 1/1E/WT E18.5 Dach1 green Prdm12 red 006.tif]

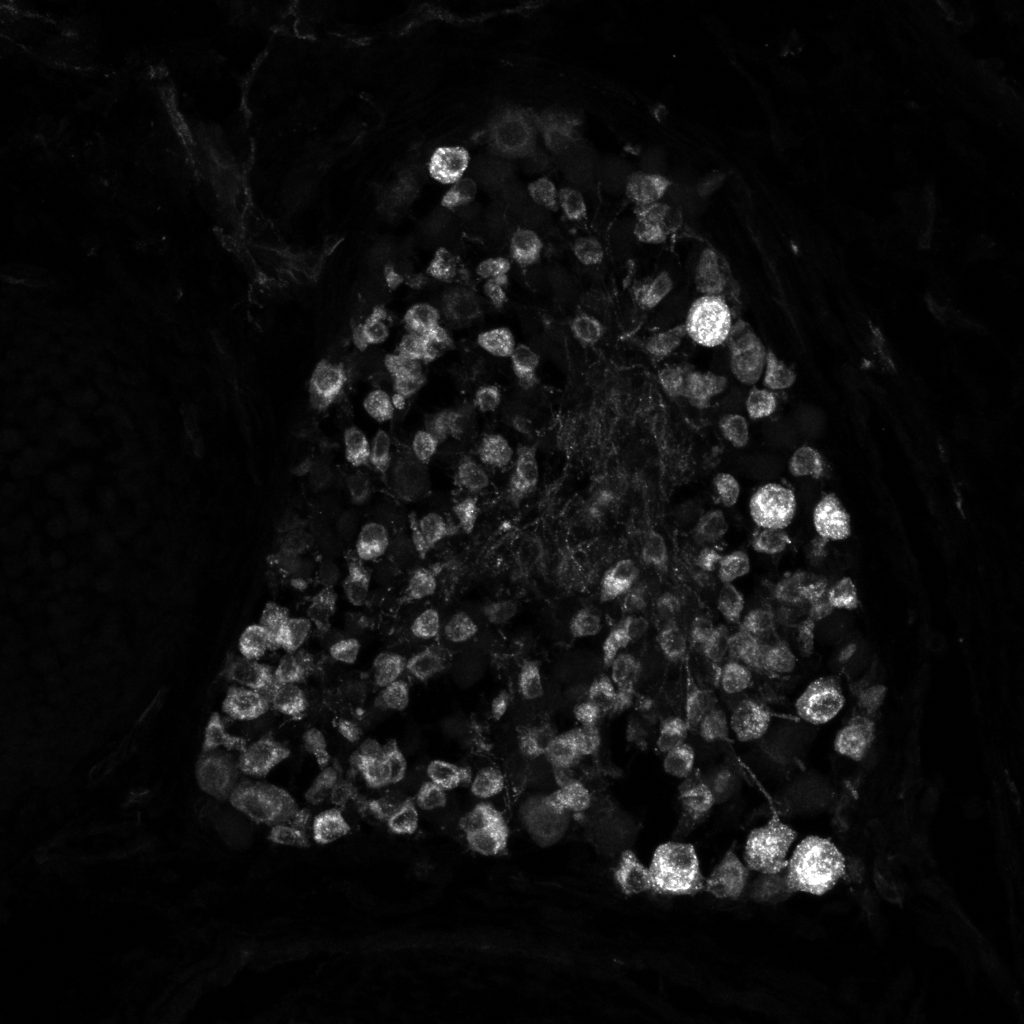

Supplement: Supplementary file 6 — Source data Fig. 1 [file 44318_2025_427_MOESM6_ESM.zip › Figure 1/1E/WT E18.5 Dach1 green Ret red 20X 002.tif]

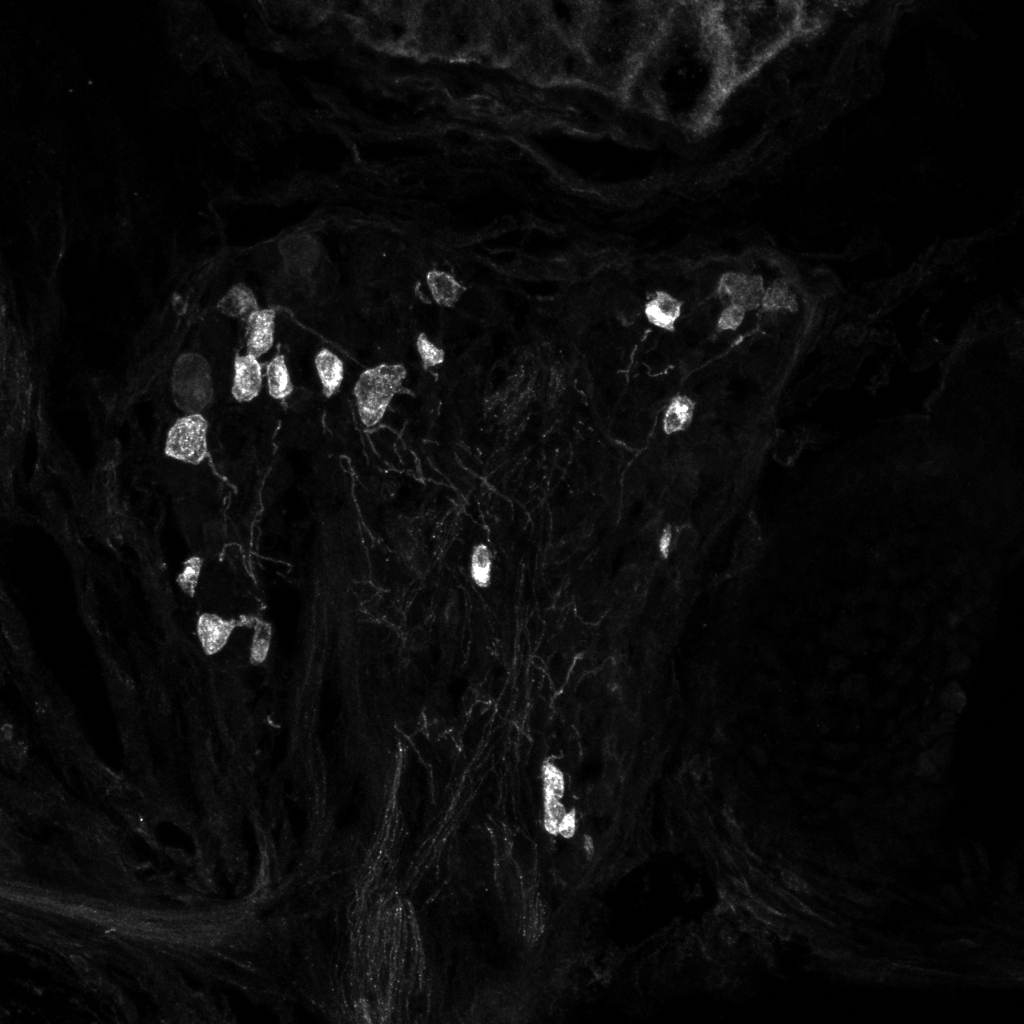

Supplement: Supplementary file 6 — Source data Fig. 1 [file 44318_2025_427_MOESM6_ESM.zip › Figure 1/1E/WT E18.5 Dach1 green TrkB red 2.tif]

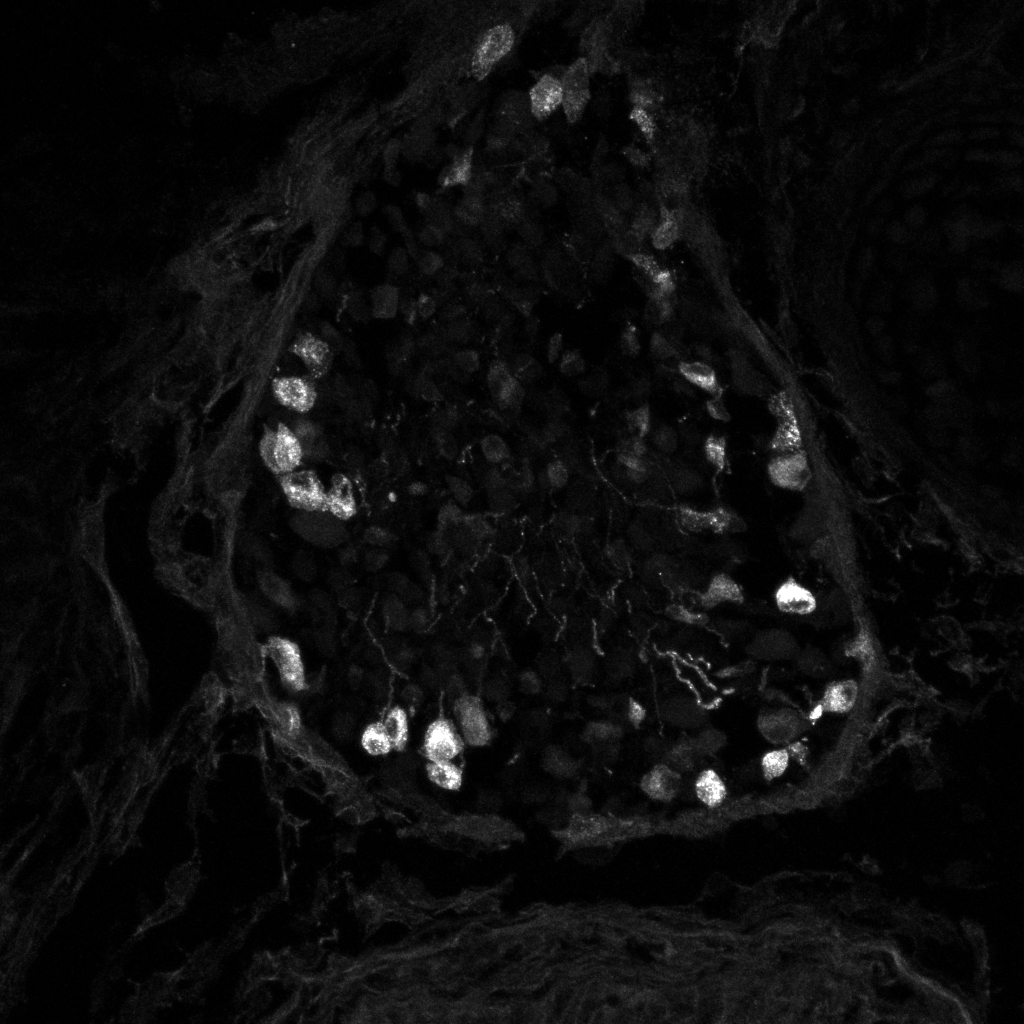

Supplement: Supplementary file 6 — Source data Fig. 1 [file 44318_2025_427_MOESM6_ESM.zip › Figure 1/1E/WT E18.5 Dach1 green TrkC red 4.tif]

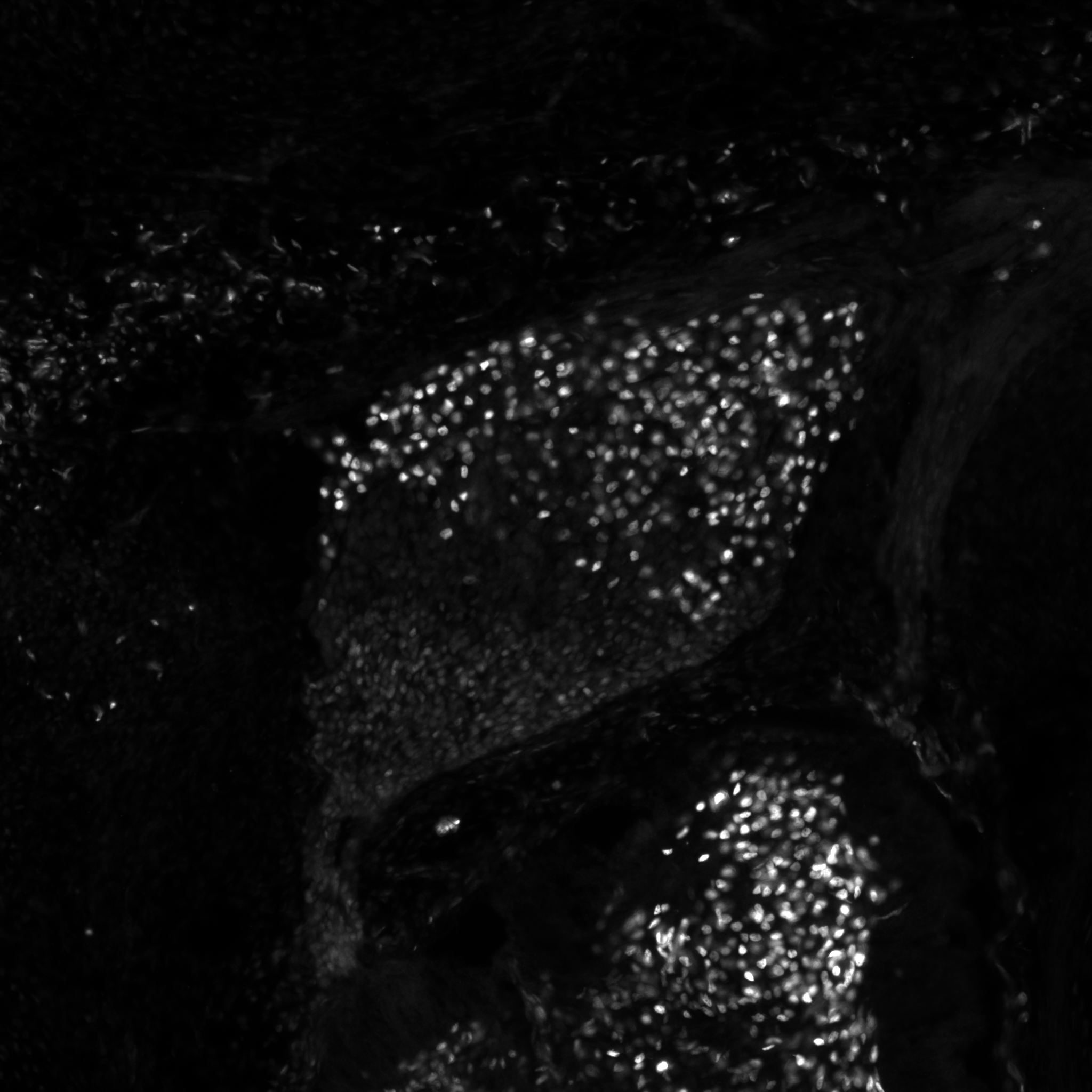

Supplement: Supplementary file 6 — Source data Fig. 1 [file 44318_2025_427_MOESM6_ESM.zip › Figure 1/1F/Snap-18930.tif]

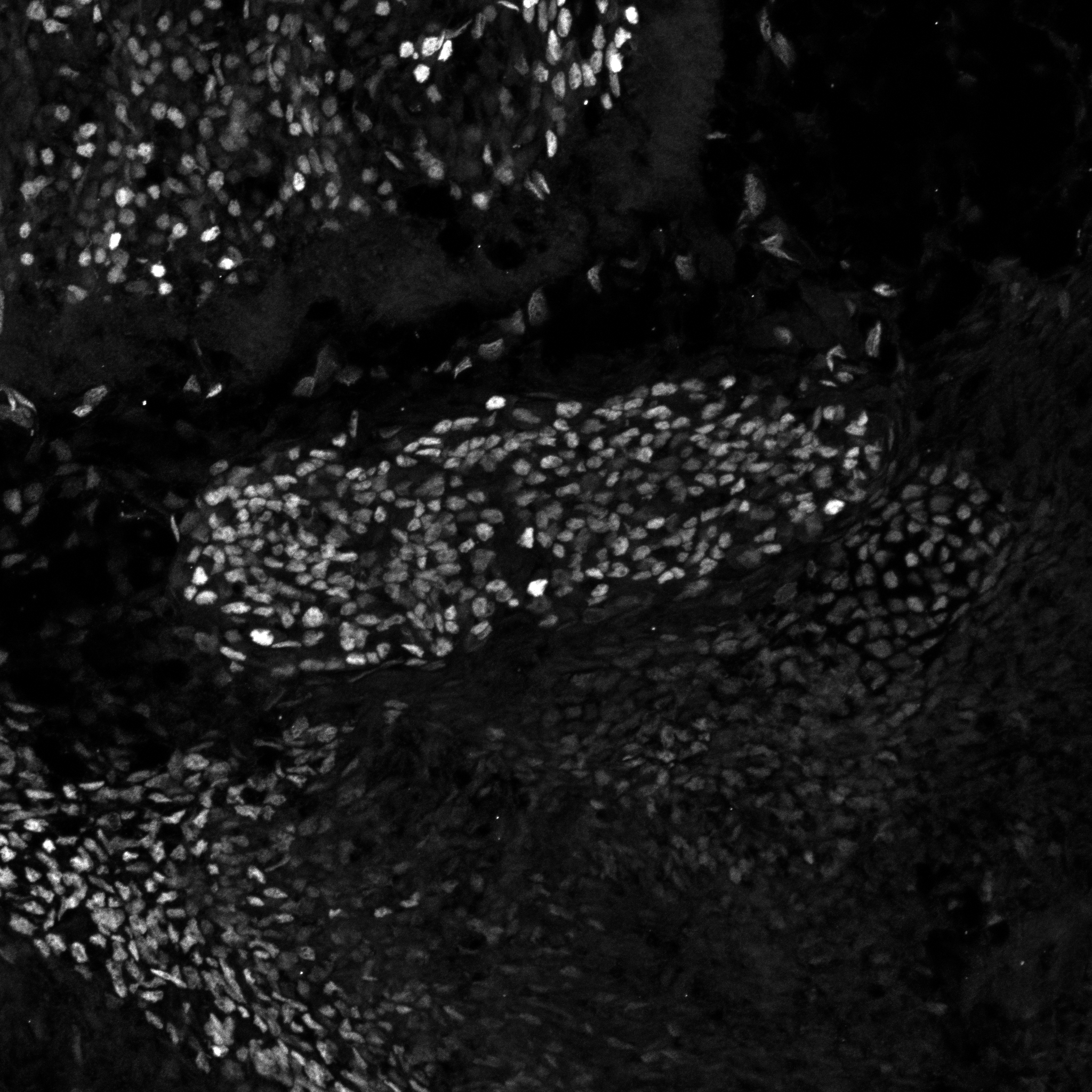

Supplement: Supplementary file 7 — Source data Fig. 2 [file 44318_2025_427_MOESM7_ESM.zip › Figure 2/2A/MAX_E12-5-TrkA+Prdm12+Dach1-2.tif]

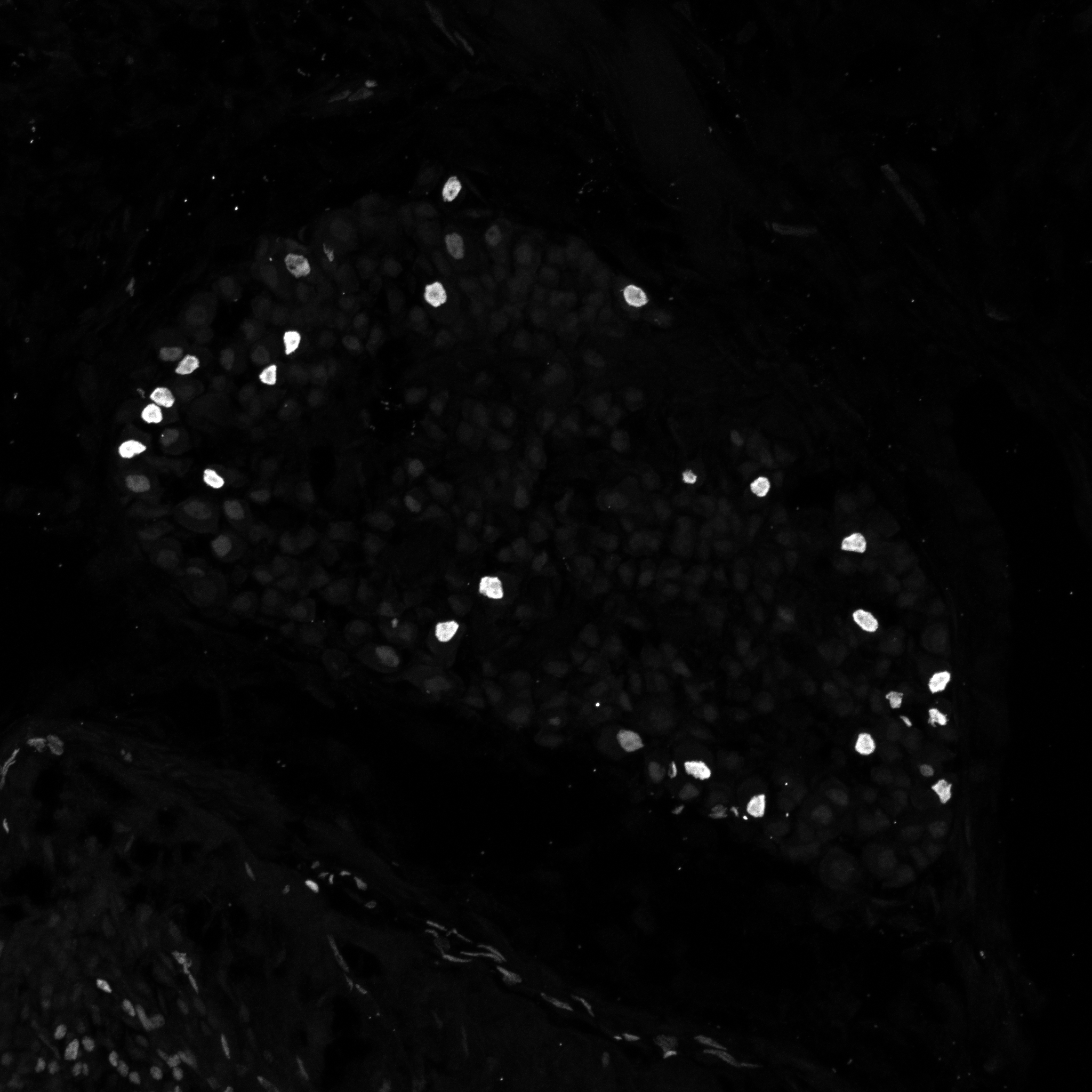

Supplement: Supplementary file 7 — Source data Fig. 2 [file 44318_2025_427_MOESM7_ESM.zip › Figure 2/2A/MAX_E16-5-TrkA+Prdm12+Dach1-2.tif]

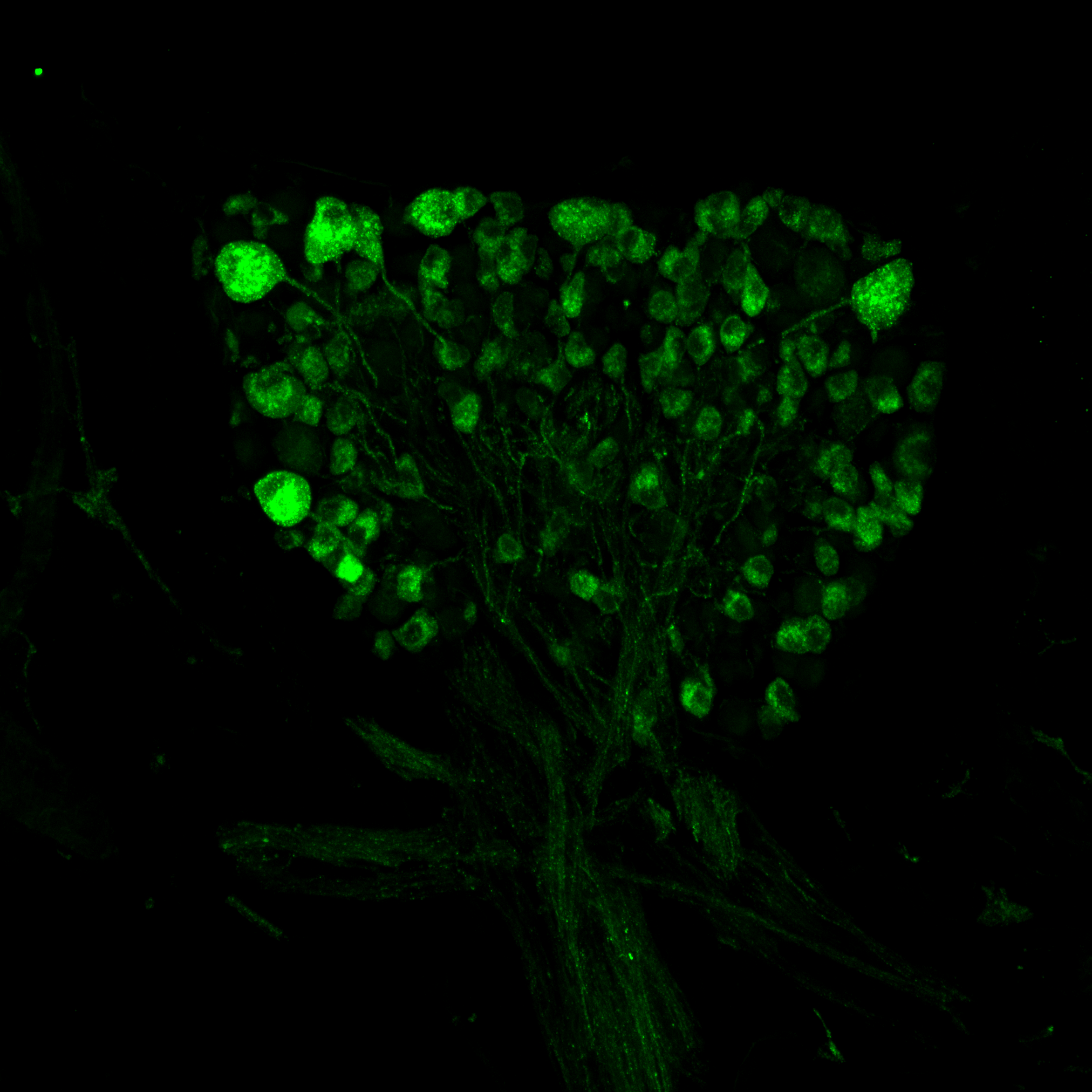

Supplement: Supplementary file 7 — Source data Fig. 2 [file 44318_2025_427_MOESM7_ESM.zip › Figure 2/2B/Ctrl E18-5- RET.tif]

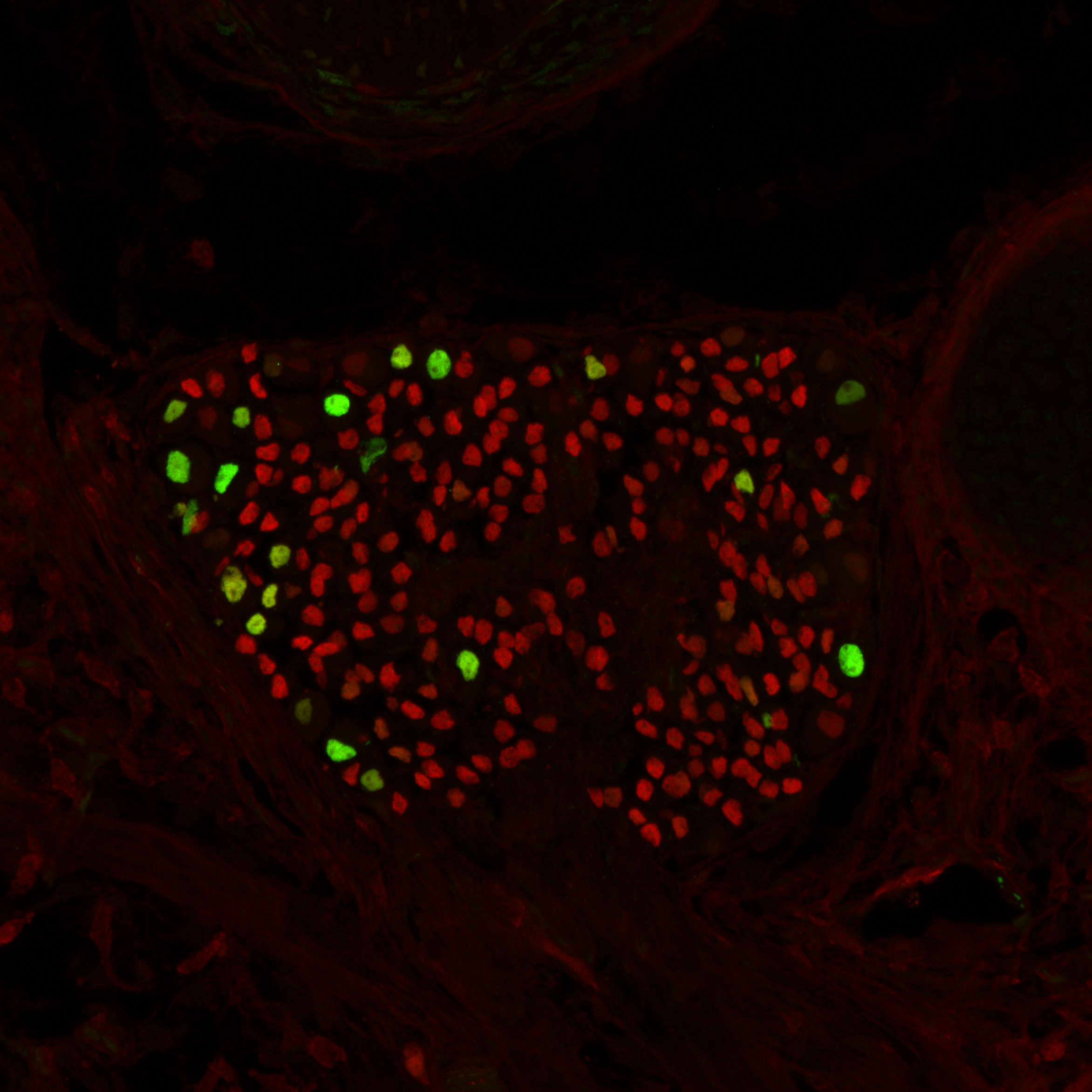

Supplement: Supplementary file 7 — Source data Fig. 2 [file 44318_2025_427_MOESM7_ESM.zip › Figure 2/2B/Ctrl-Dach1+Isl1-E18-5.tif]

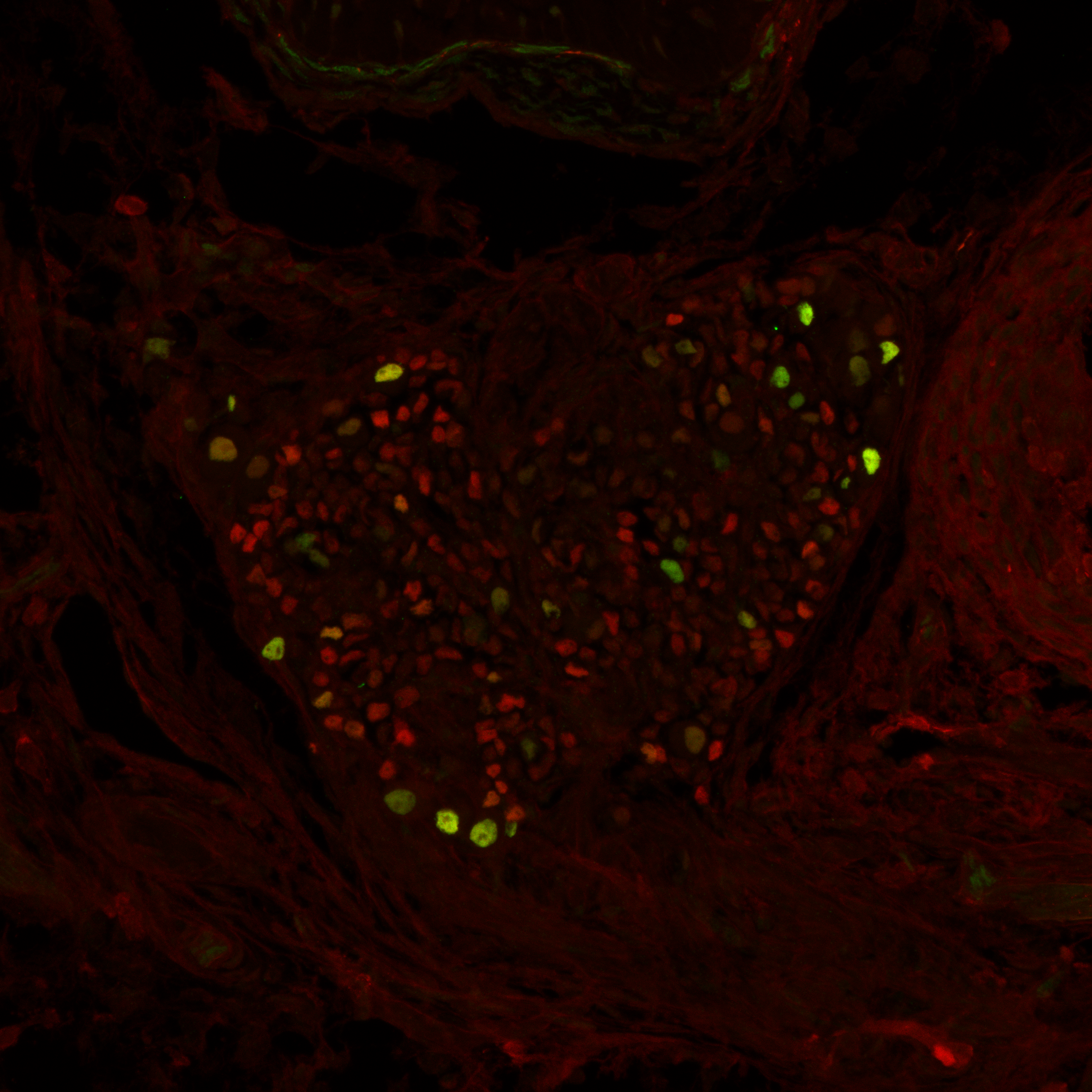

Supplement: Supplementary file 7 — Source data Fig. 2 [file 44318_2025_427_MOESM7_ESM.zip › Figure 2/2B/NGF-Bax-Dach1+Isl1-E18-5.tif]

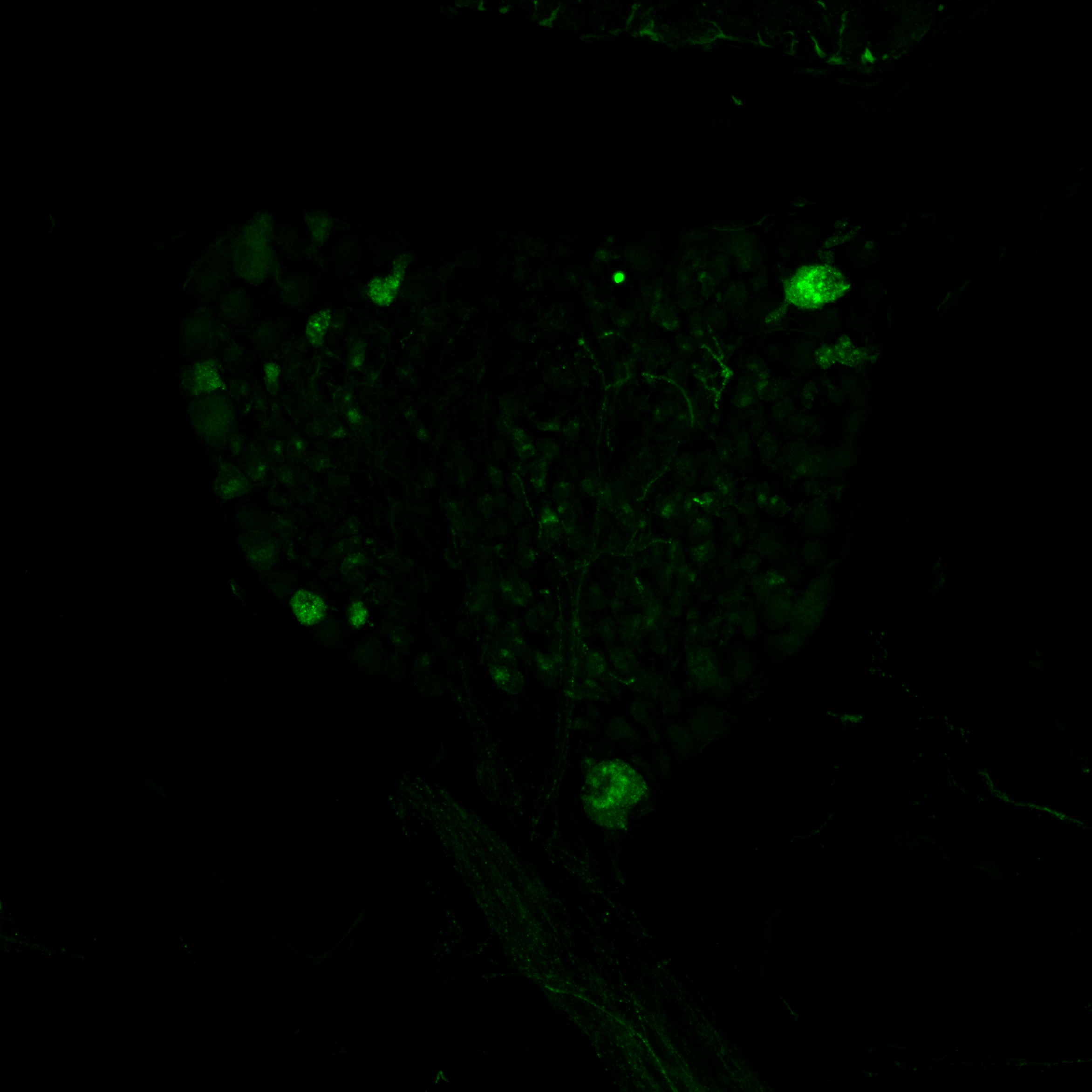

Supplement: Supplementary file 7 — Source data Fig. 2 [file 44318_2025_427_MOESM7_ESM.zip › Figure 2/2B/NGFBax E18.5- RET.tif]

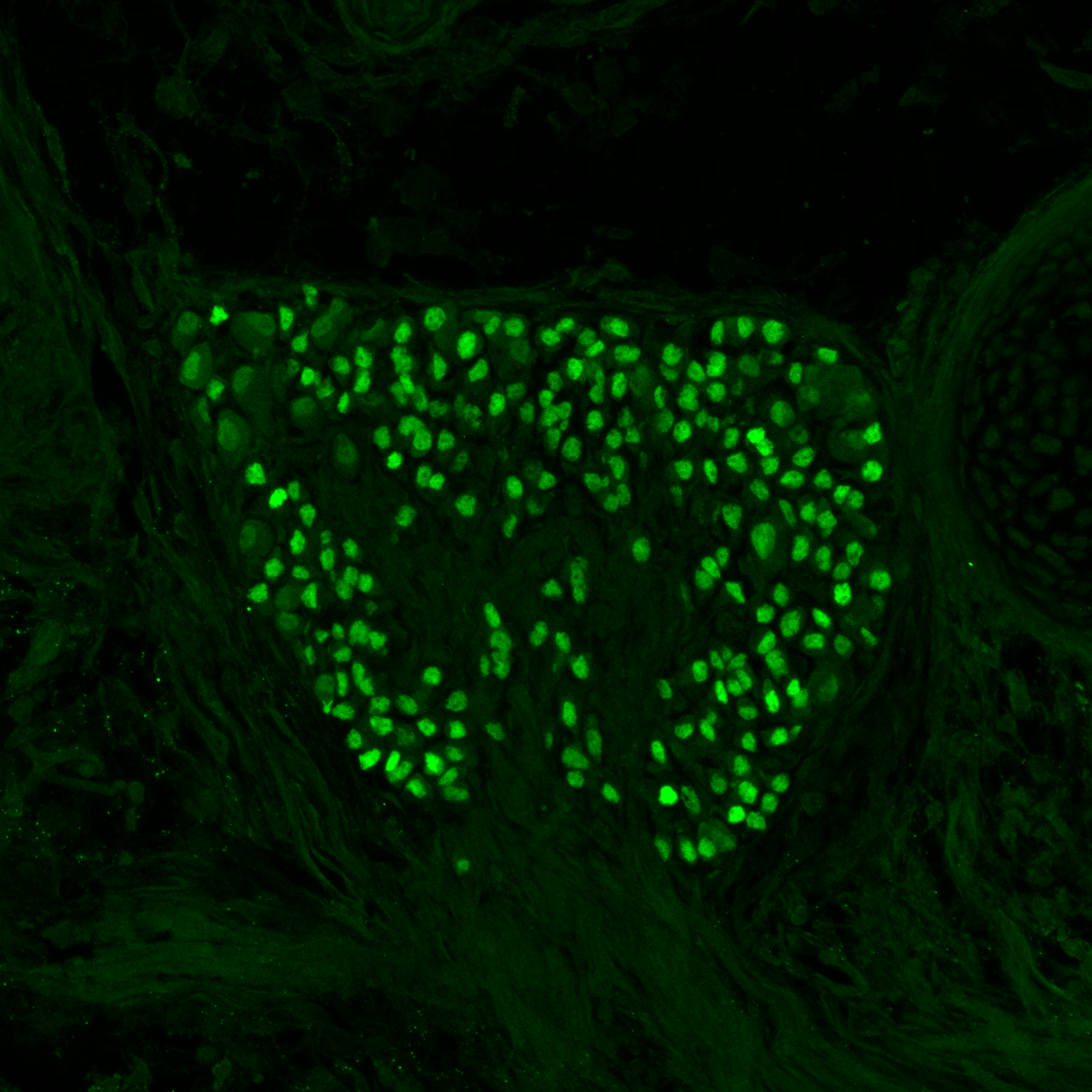

Supplement: Supplementary file 7 — Source data Fig. 2 [file 44318_2025_427_MOESM7_ESM.zip › Figure 2/2C/MAX_Ctrl 2 E18.5- IF Prdm12-2.tif]

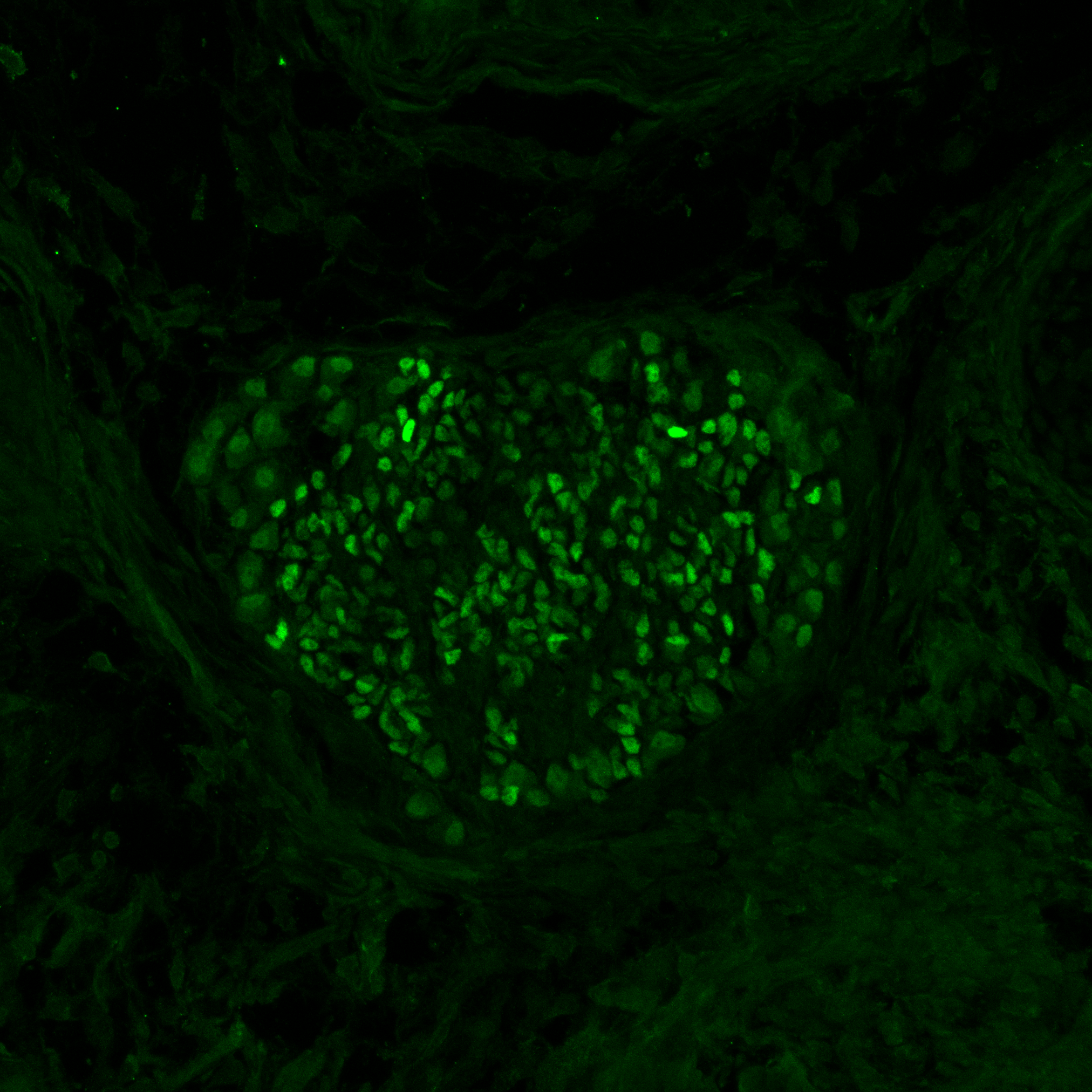

Supplement: Supplementary file 7 — Source data Fig. 2 [file 44318_2025_427_MOESM7_ESM.zip › Figure 2/2C/MAX_NGFBax 2 E18.5- IF Prdm12.tif]

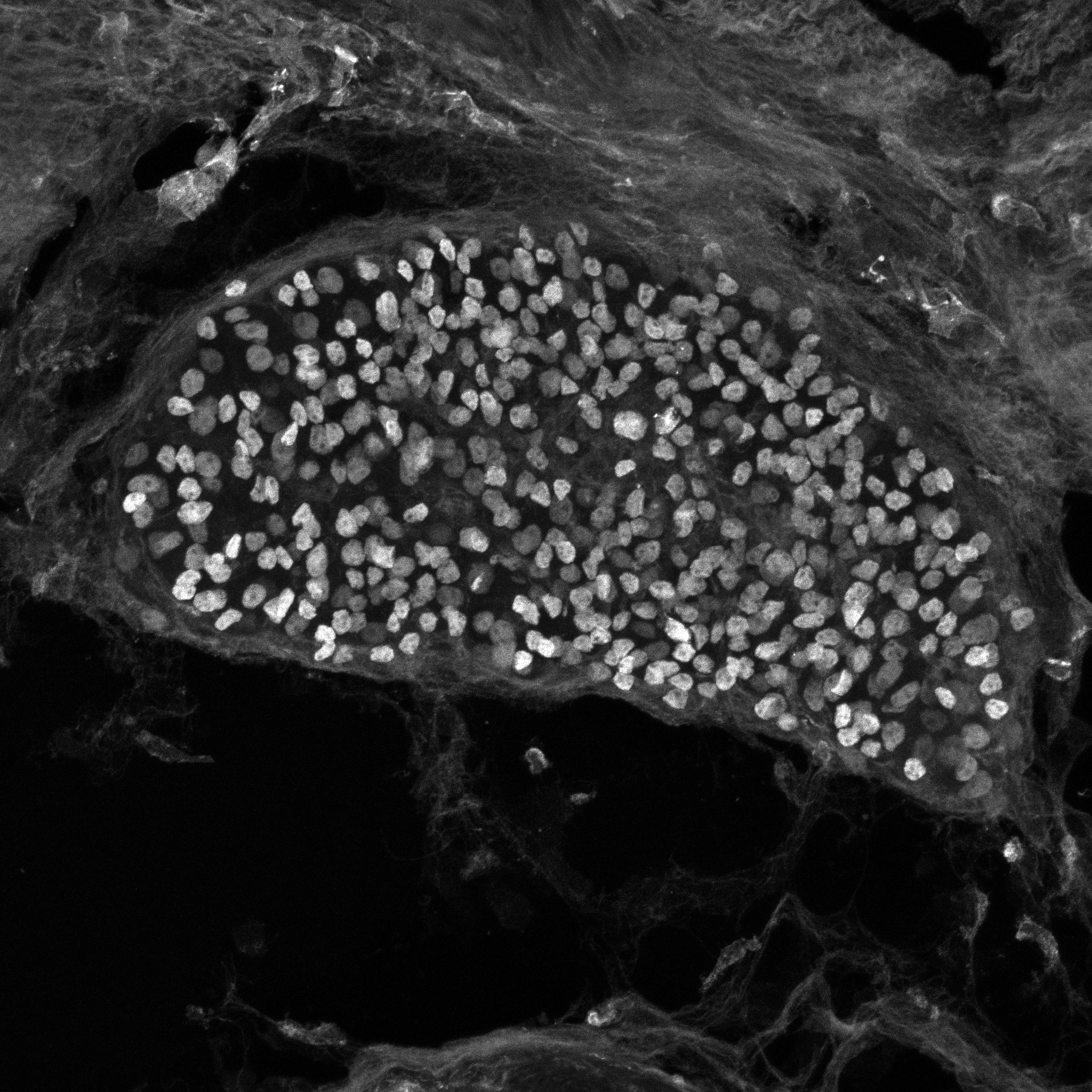

Supplement: Supplementary file 7 — Source data Fig. 2 [file 44318_2025_427_MOESM7_ESM.zip › Figure 2/2D/MAX_Ctrl-IF Dach1+Isl1 - E18-5_2.tif]

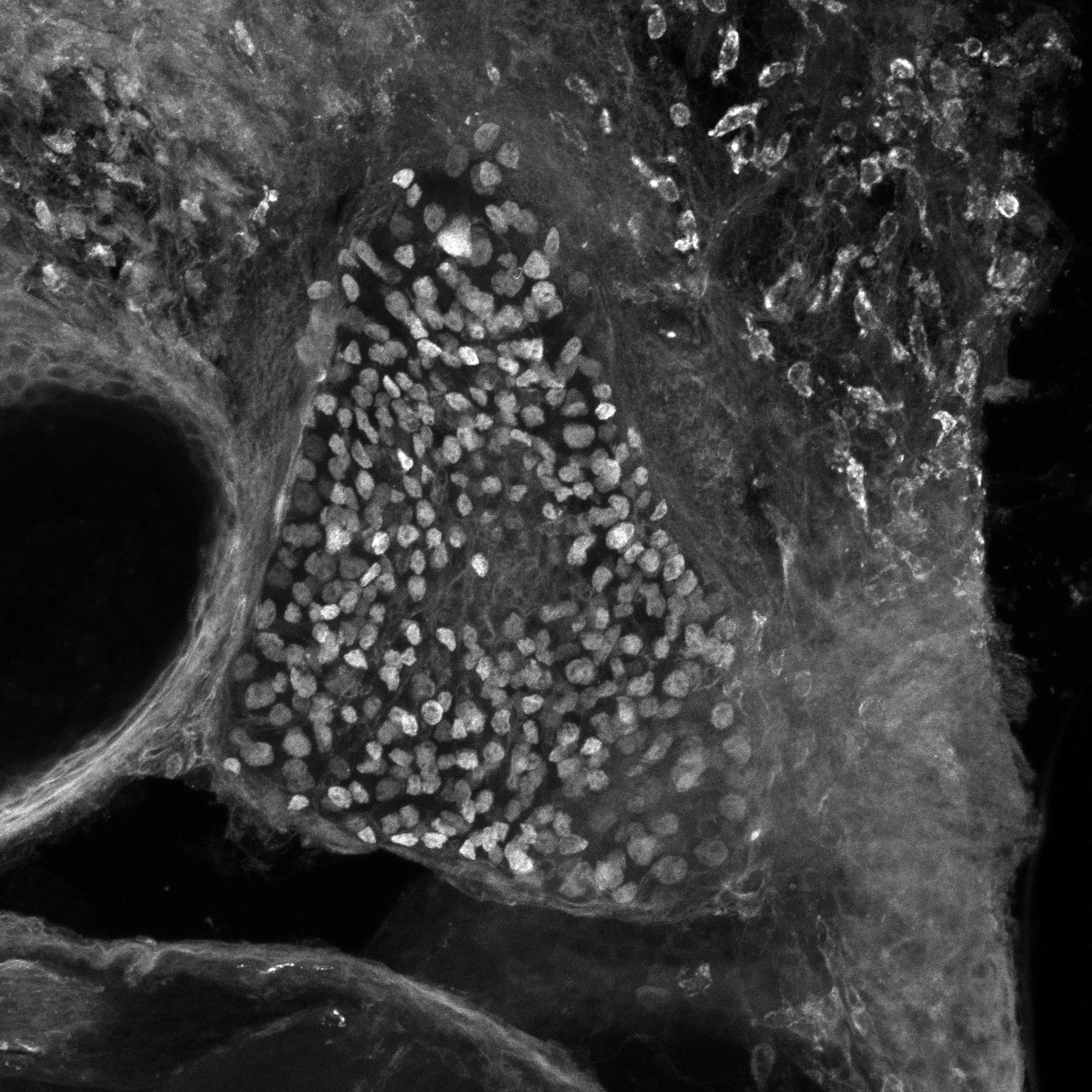

Supplement: Supplementary file 7 — Source data Fig. 2 [file 44318_2025_427_MOESM7_ESM.zip › Figure 2/2D/MAX_nav1-8_P12 cko-IF Dach1+Isl1 - E18-5.tif]

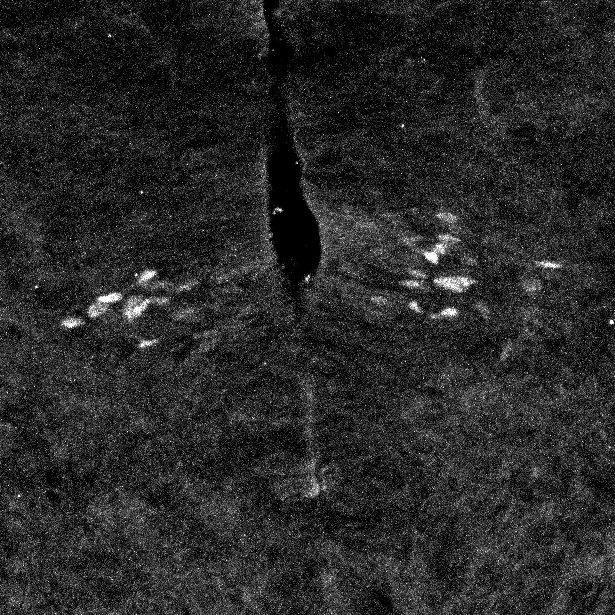

Supplement: Supplementary file 7 — Source data Fig. 2 [file 44318_2025_427_MOESM7_ESM.zip › Figure 2/2I/MAX_P12 KI E12.5 Prdm12 red V5 green.tif]

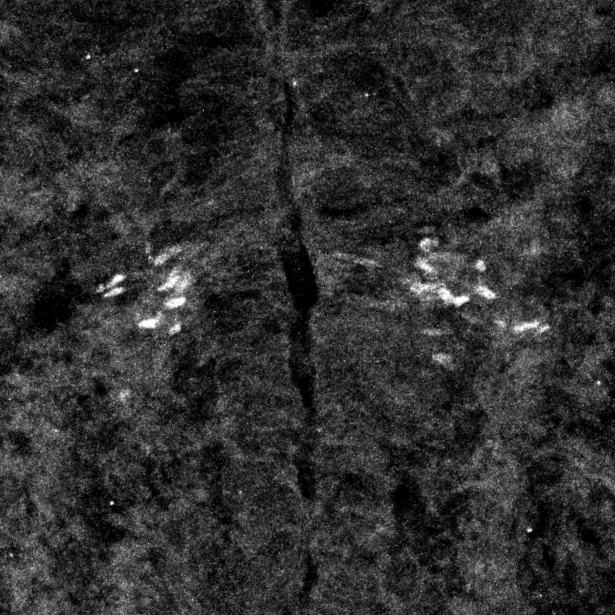

Supplement: Supplementary file 7 — Source data Fig. 2 [file 44318_2025_427_MOESM7_ESM.zip › Figure 2/2I/MAX_P12 WT E12.5 Prdm12 red V5 green 001.tif]

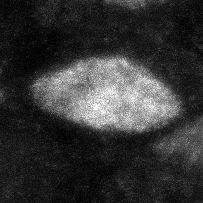

Supplement: Supplementary file 7 — Source data Fig. 2 [file 44318_2025_427_MOESM7_ESM.zip › Figure 2/2J/MAX_P12 KI 1 E12.5 Prdm12 red V5 green 001 63x-1.tif]

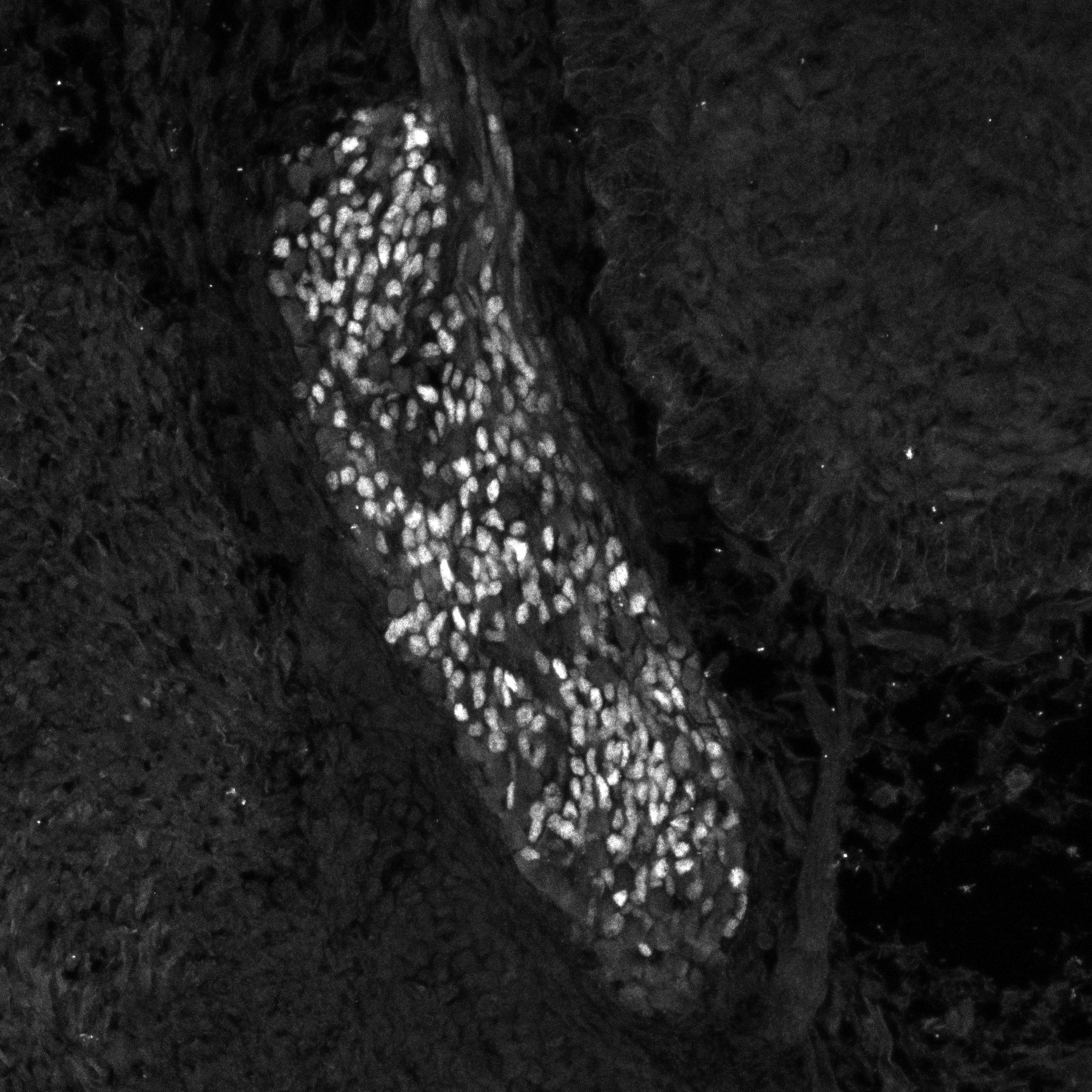

Supplement: Supplementary file 7 — Source data Fig. 2 [file 44318_2025_427_MOESM7_ESM.zip › Figure 2/2J/MAX_P12 KI 1 E12.5 Prdm12 red V5 green 001.tif]

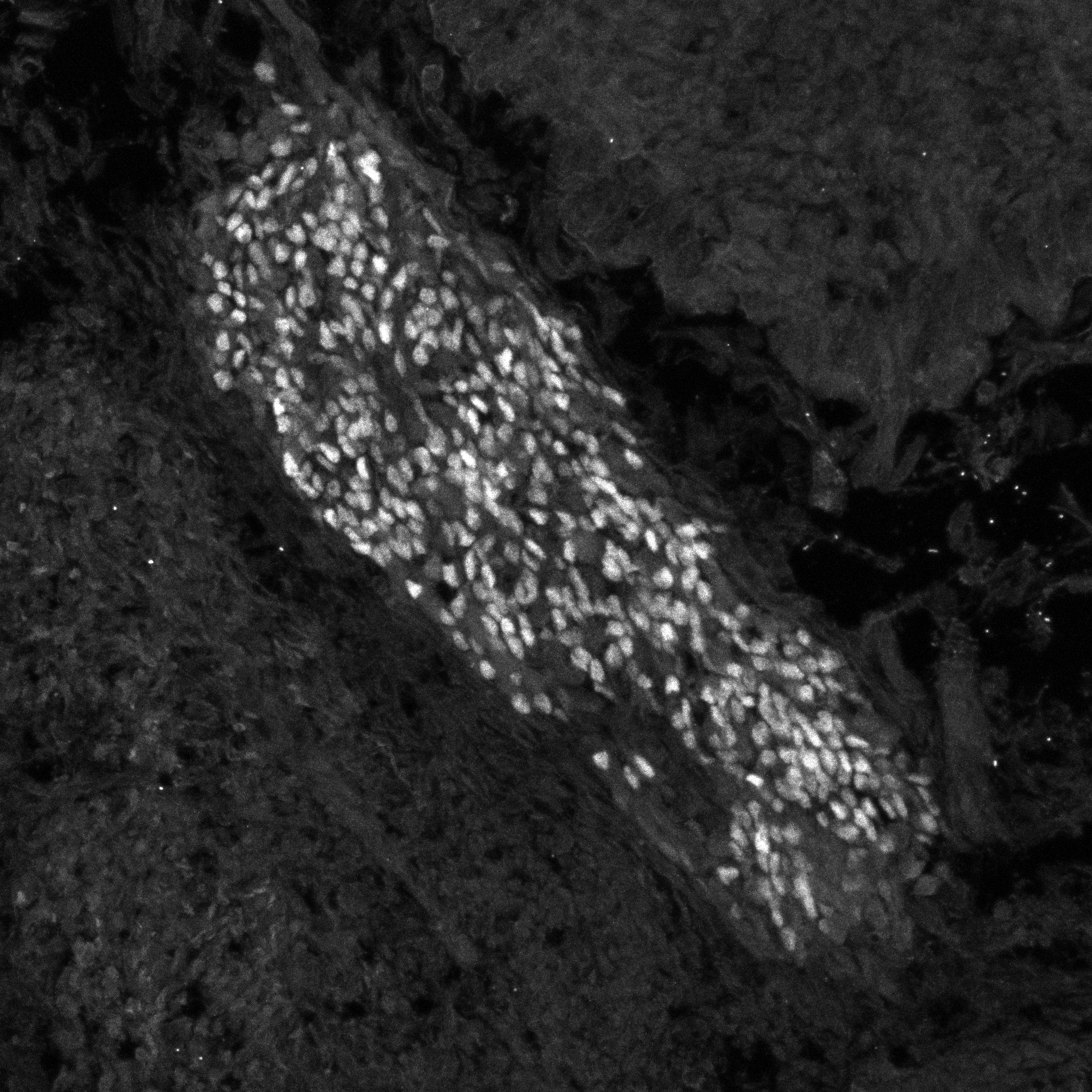

Supplement: Supplementary file 7 — Source data Fig. 2 [file 44318_2025_427_MOESM7_ESM.zip › Figure 2/2J/MAX_P12 WT E12.5 Prdm12 red V5 green.tif]

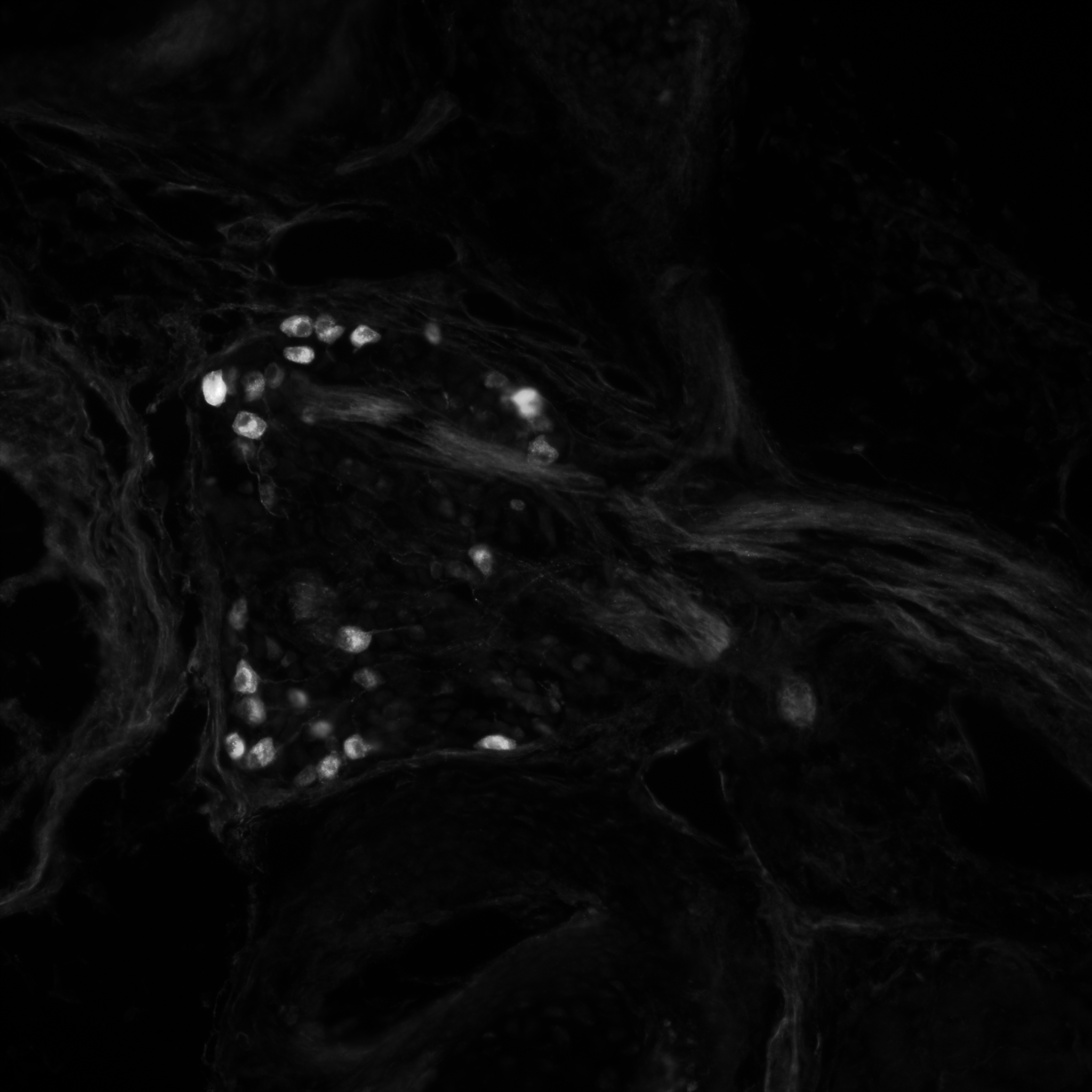

Supplement: Supplementary file 8 — Source data Fig. 3 [file 44318_2025_427_MOESM8_ESM.zip › Figure 3/3A/AD1 cKO#2 E18.5 Parvalbumin green TrkC red 3d.tif]

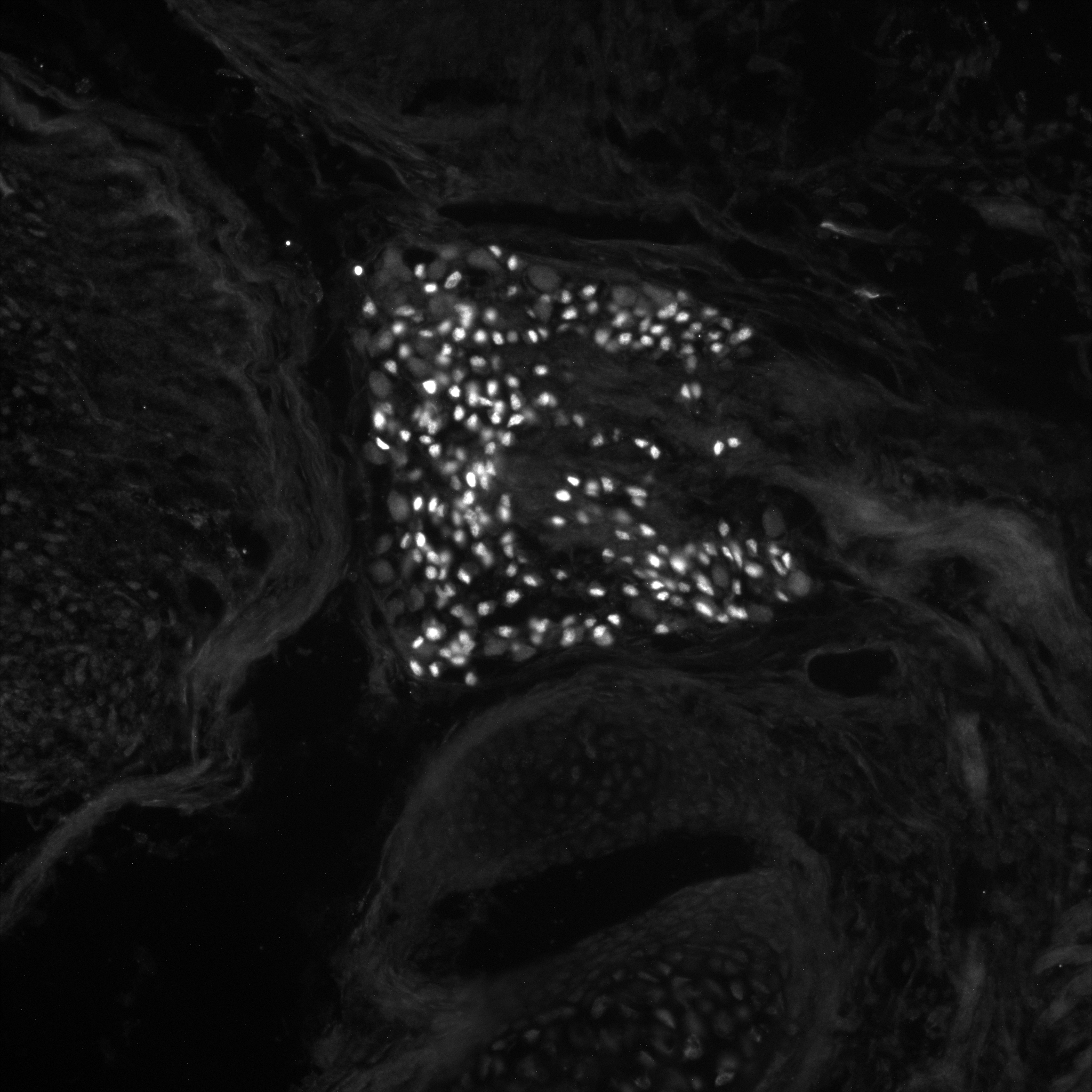

Supplement: Supplementary file 8 — Source data Fig. 3 [file 44318_2025_427_MOESM8_ESM.zip › Figure 3/3A/AD1 cKO#2 E18.5 Prdm12 red Mef2c green 11g.tif]

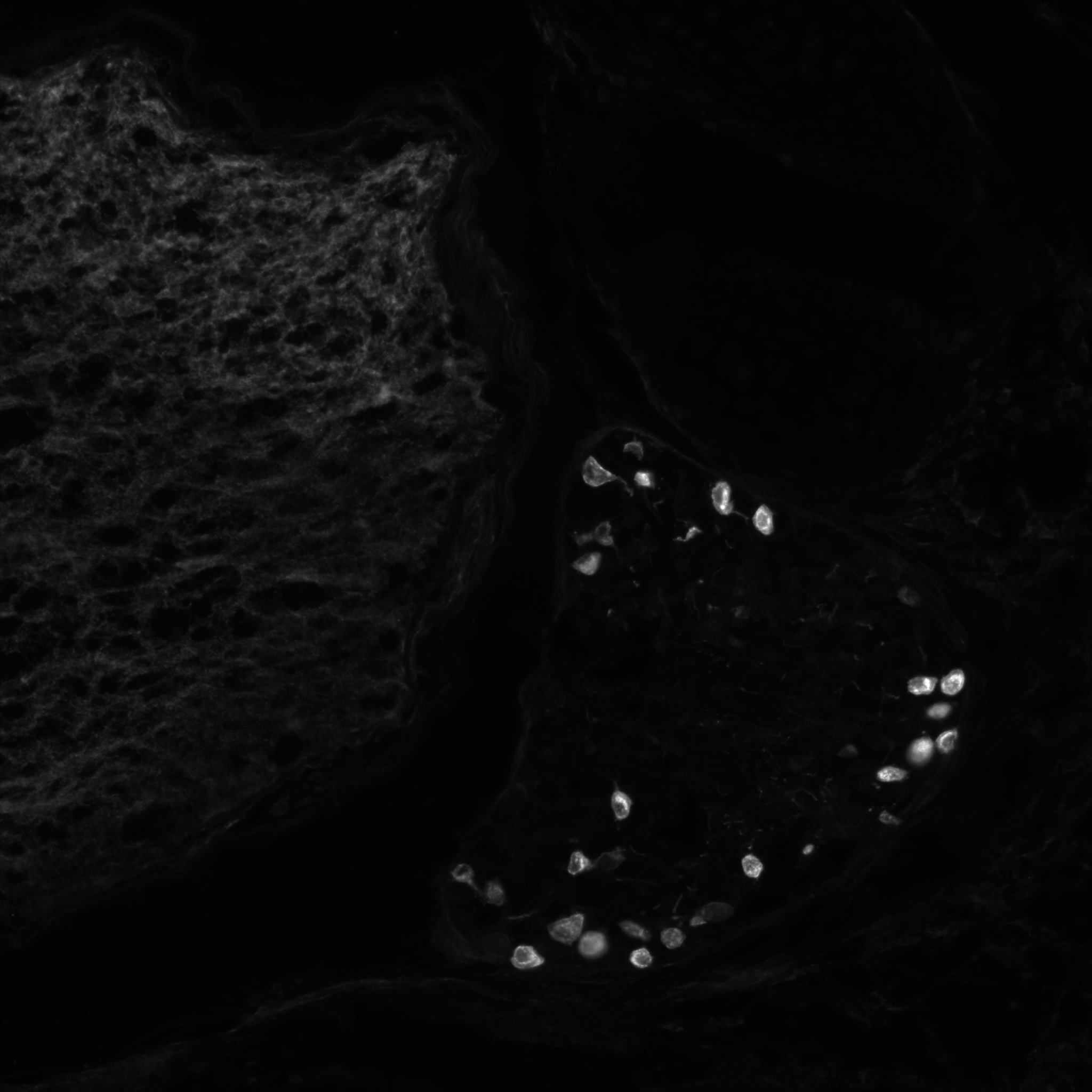

Supplement: Supplementary file 8 — Source data Fig. 3 [file 44318_2025_427_MOESM8_ESM.zip › Figure 3/3A/AD1 cKO#6 E18.5 TH green TrkB red 3d.tif]

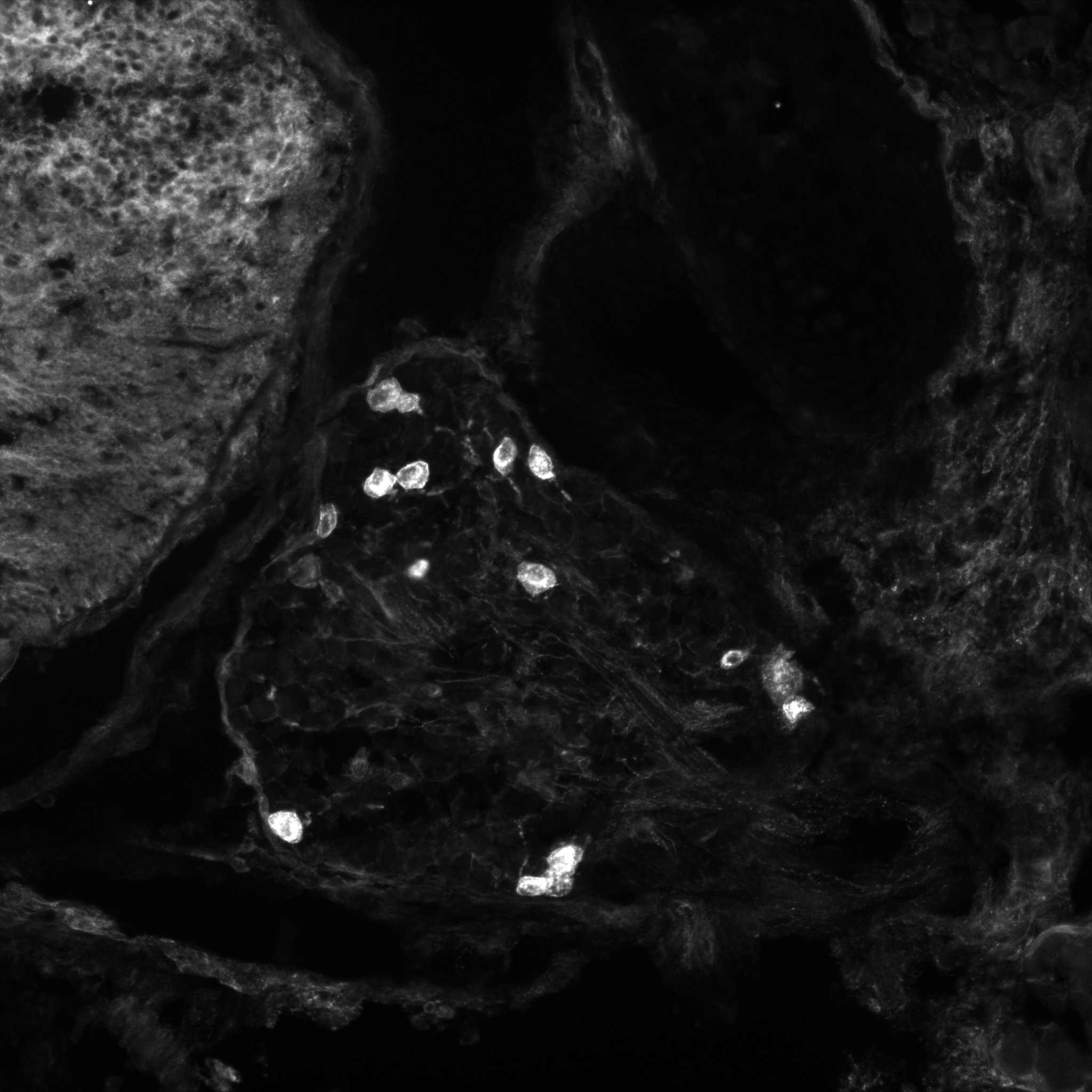

Supplement: Supplementary file 8 — Source data Fig. 3 [file 44318_2025_427_MOESM8_ESM.zip › Figure 3/3A/W1D1 cKO#18 E18.5 Mef2c green TrkB red 11g.tif]

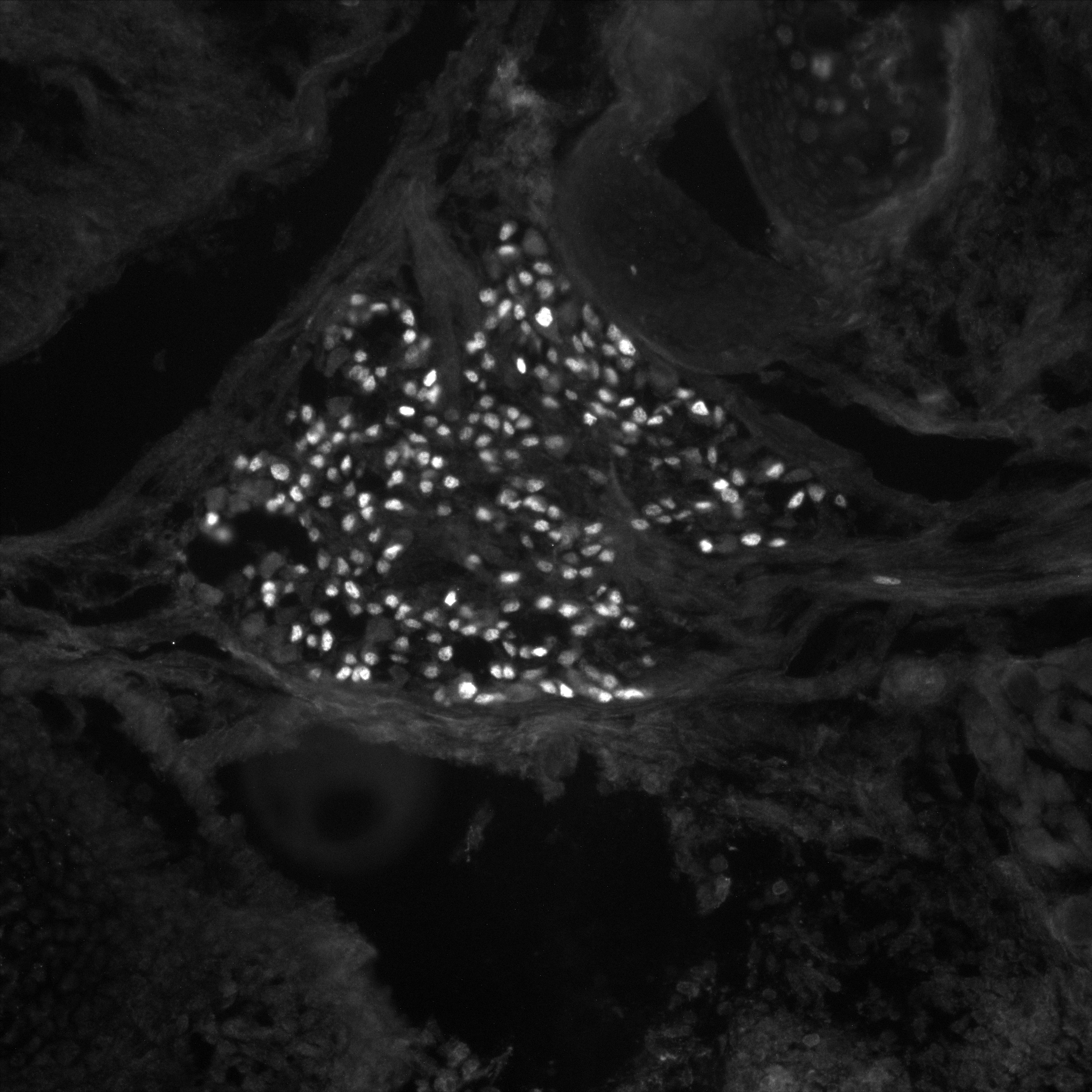

Supplement: Supplementary file 8 — Source data Fig. 3 [file 44318_2025_427_MOESM8_ESM.zip › Figure 3/3A/W1D1 cKO#18 E18.5 Prdm12 red TH green 6d.tif]

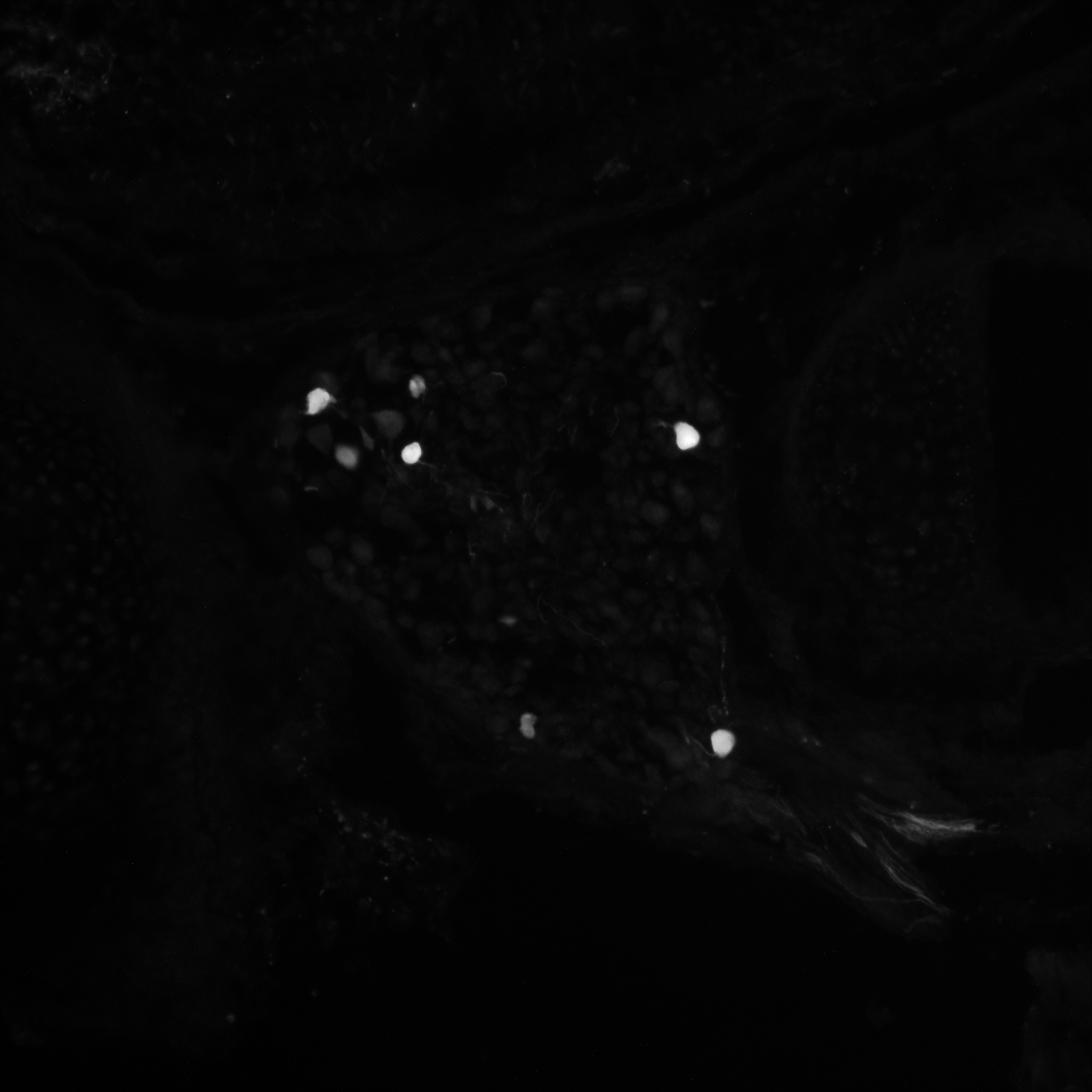

Supplement: Supplementary file 8 — Source data Fig. 3 [file 44318_2025_427_MOESM8_ESM.zip › Figure 3/3A/W1D1 cKO#22 E18.5 PV red 2g.tif]

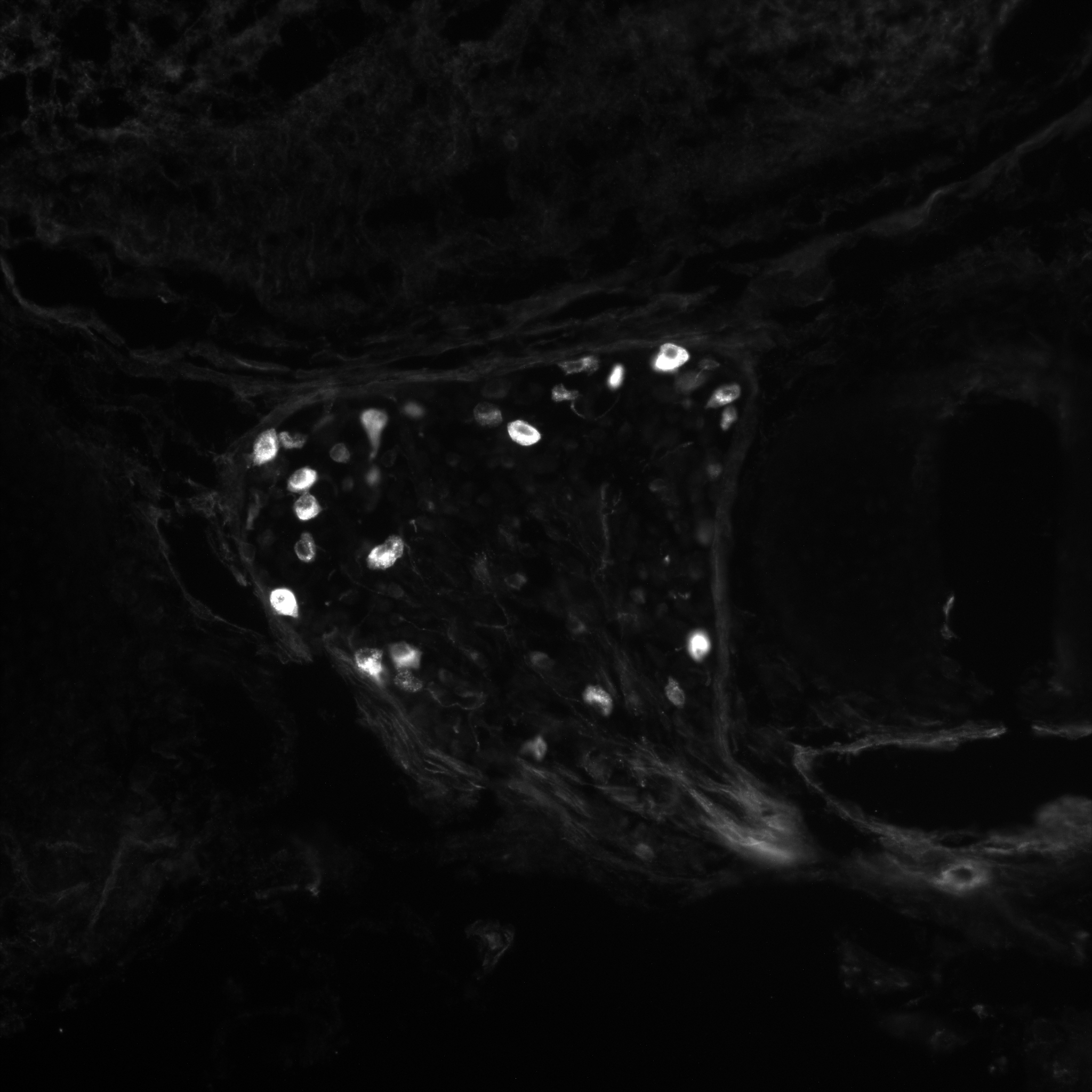

Supplement: Supplementary file 8 — Source data Fig. 3 [file 44318_2025_427_MOESM8_ESM.zip › Figure 3/3A/W1D1 cKO#22 E18.5 TrkC red 11d.tif]

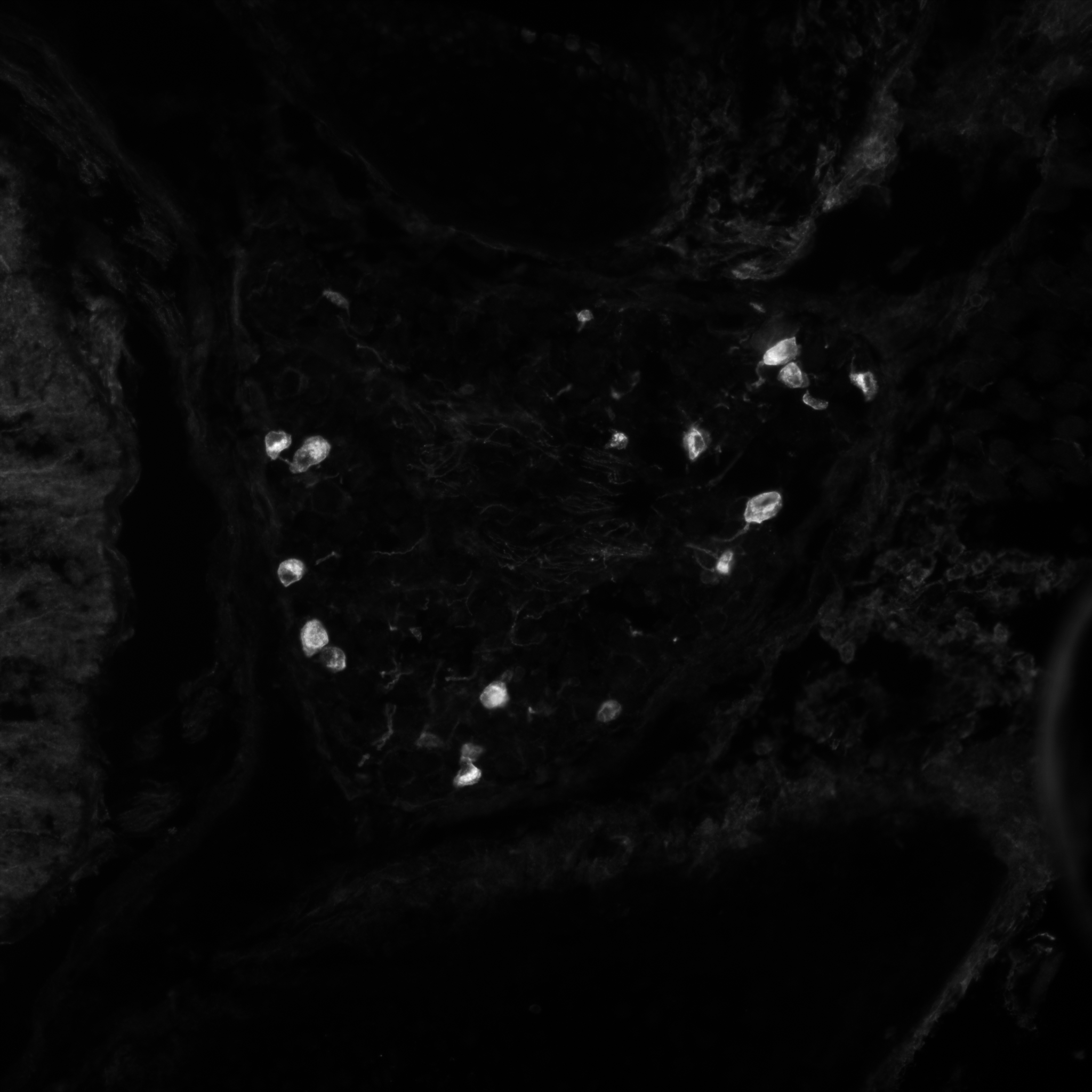

Supplement: Supplementary file 8 — Source data Fig. 3 [file 44318_2025_427_MOESM8_ESM.zip › Figure 3/3A/W1D1 ctrl#19 E18.5 Mef2c green TrkB red 8g.tif]

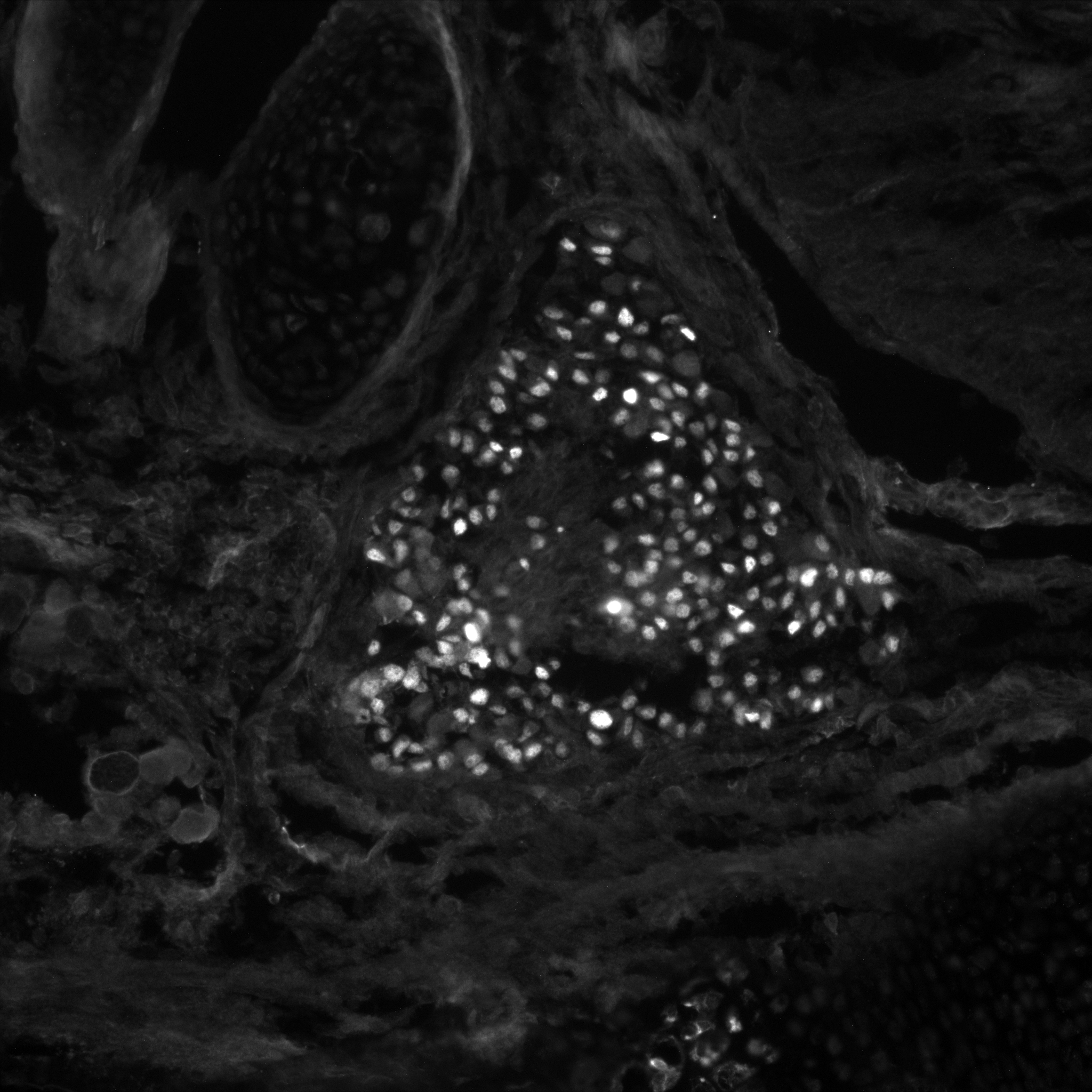

Supplement: Supplementary file 8 — Source data Fig. 3 [file 44318_2025_427_MOESM8_ESM.zip › Figure 3/3A/W1D1 ctrl#19 Prdm12 red TH green 6g.tif]

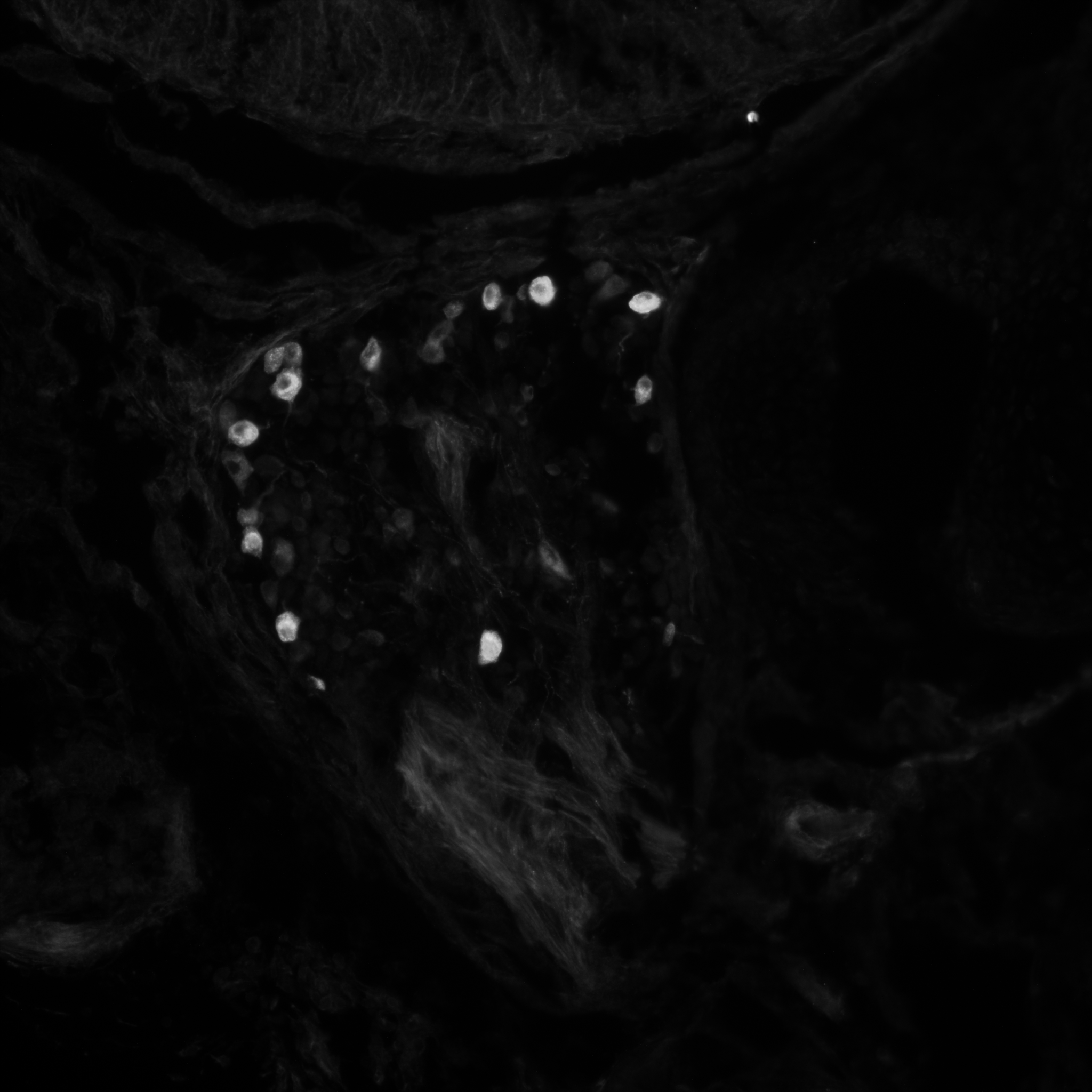

Supplement: Supplementary file 8 — Source data Fig. 3 [file 44318_2025_427_MOESM8_ESM.zip › Figure 3/3A/W1D1 ctrl#21 E18.5 TrkC red 11d.tif]

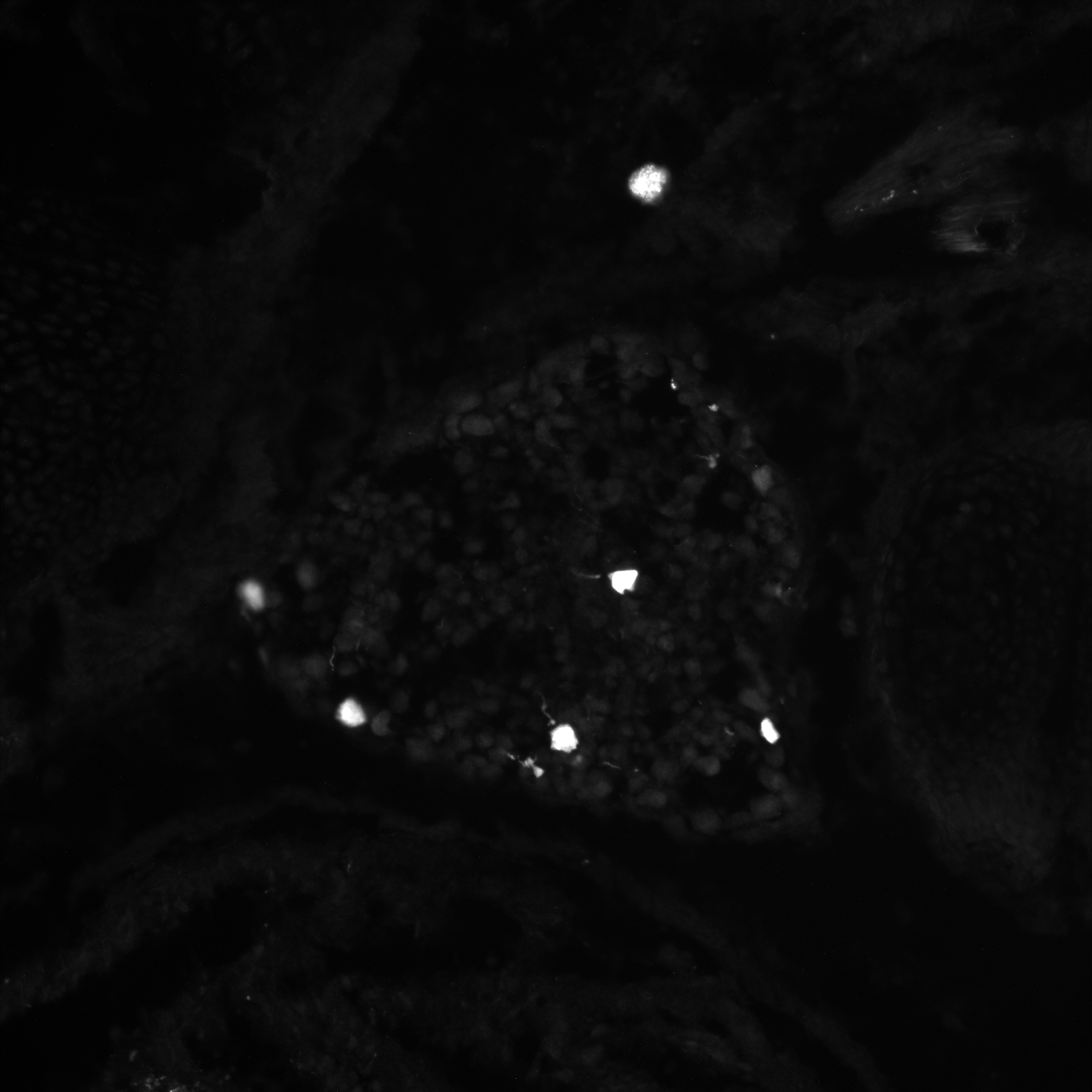

Supplement: Supplementary file 8 — Source data Fig. 3 [file 44318_2025_427_MOESM8_ESM.zip › Figure 3/3A/W1D1 ctrl#25 E18.5 PV red 8d.tif]

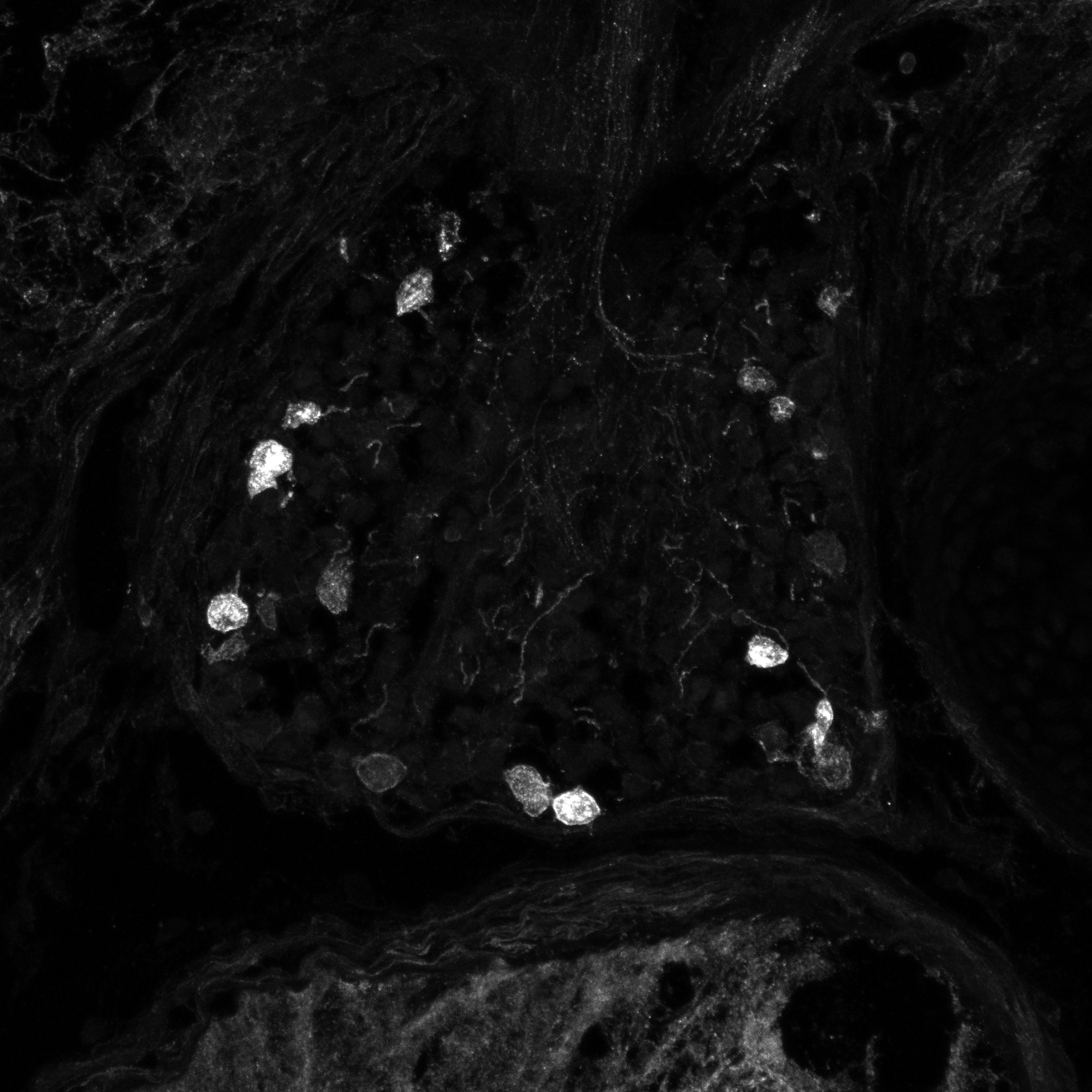

Supplement: Supplementary file 9 — Source data Fig. 4 [file 44318_2025_427_MOESM9_ESM.zip › Figure 4/4D/MAX_AD1 cKO #2 Mef2c green TrkB red 20X 001.tif]

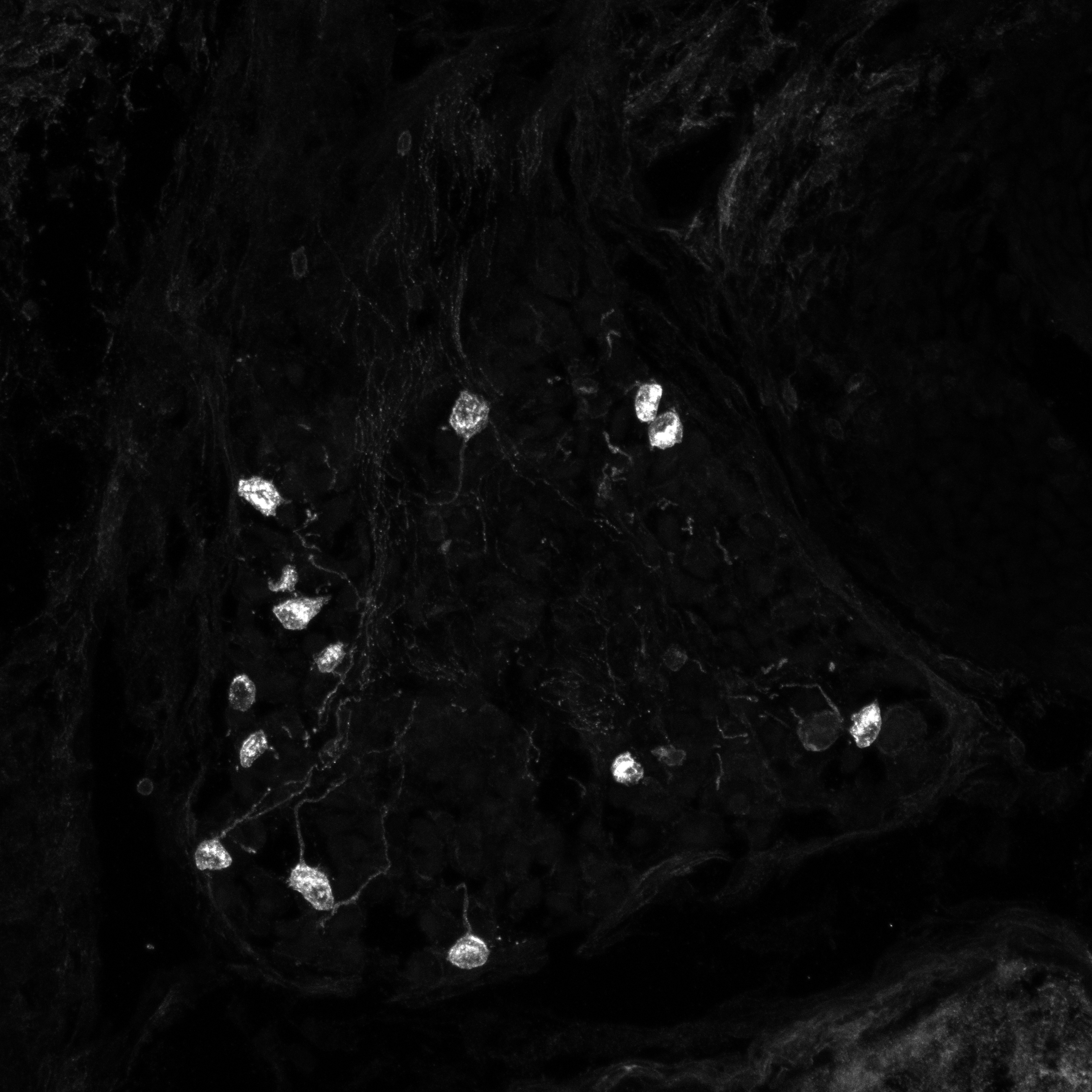

Supplement: Supplementary file 9 — Source data Fig. 4 [file 44318_2025_427_MOESM9_ESM.zip › Figure 4/4D/MAX_ctrl #2 Mef2c green TrkB red 20X 003.tif]

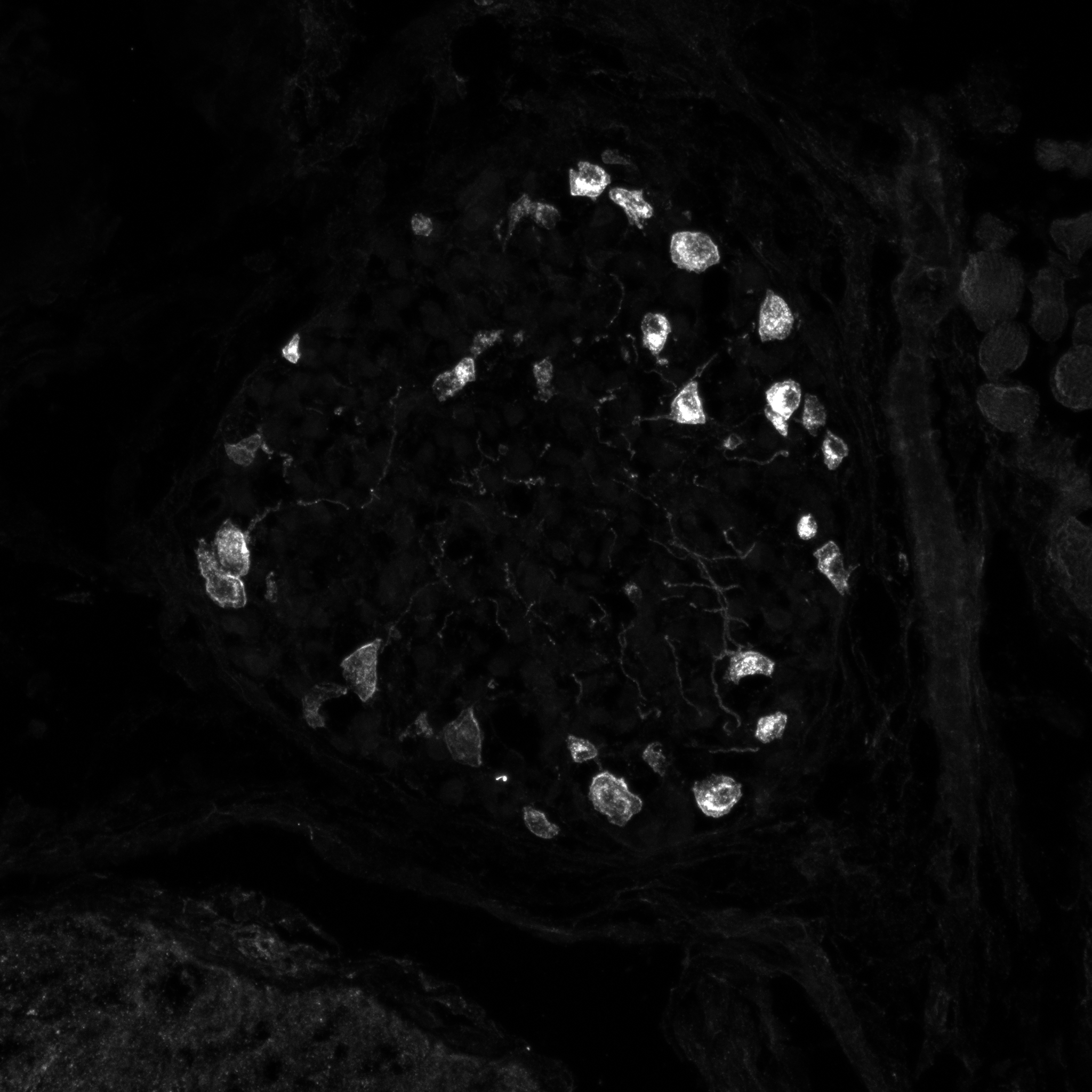

Supplement: Supplementary file 9 — Source data Fig. 4 [file 44318_2025_427_MOESM9_ESM.zip › Figure 4/4D/MAX_W1D1 cKO 30 TrkB red Mef2c green 002.tif]

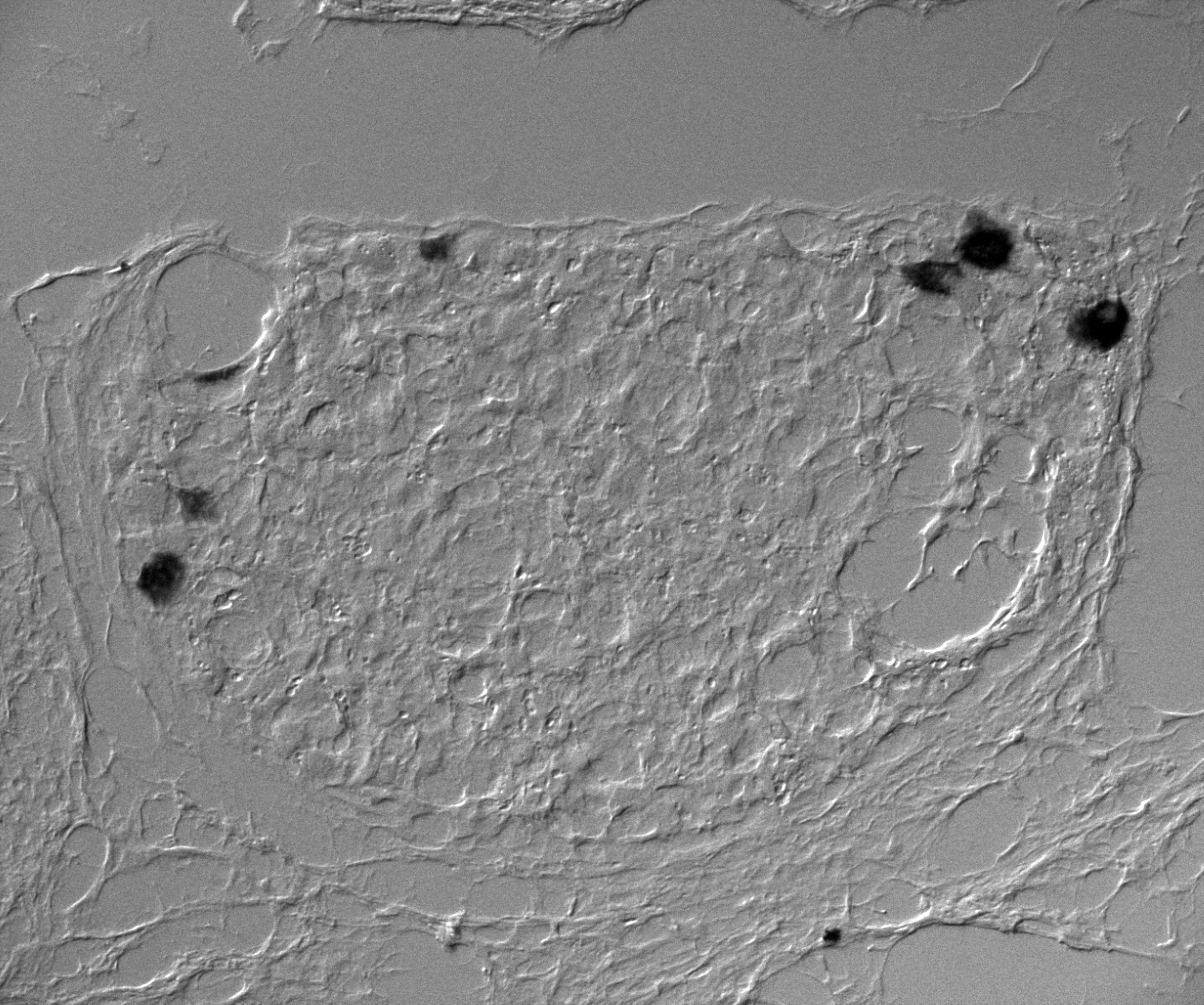

Supplement: Supplementary file 9 — Source data Fig. 4 [file 44318_2025_427_MOESM9_ESM.zip › Figure 4/4F/AD1 cko 33 Chodl 029.tif]

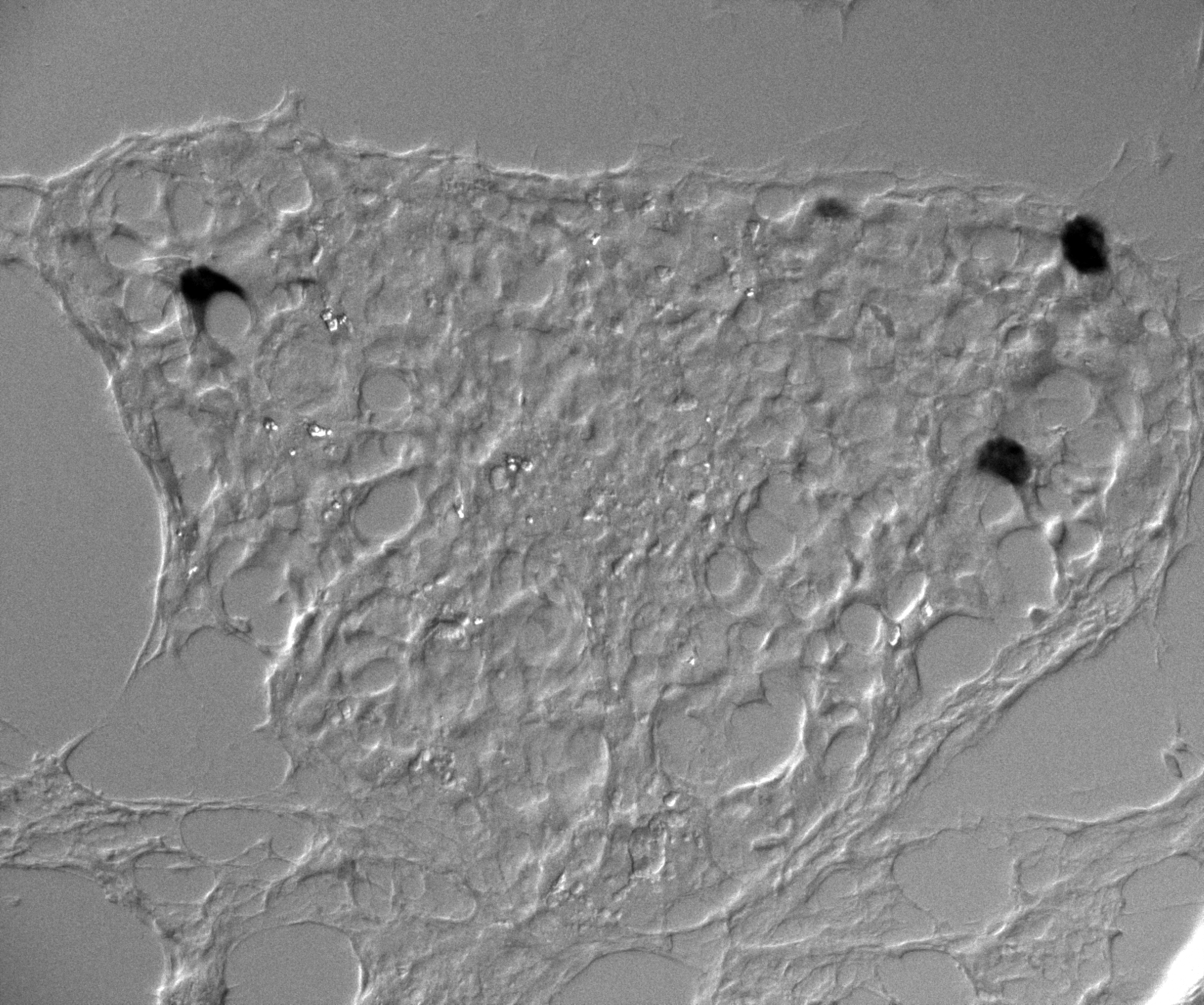

Supplement: Supplementary file 9 — Source data Fig. 4 [file 44318_2025_427_MOESM9_ESM.zip › Figure 4/4F/AD1 WT 32 Chodl 8d.tif]

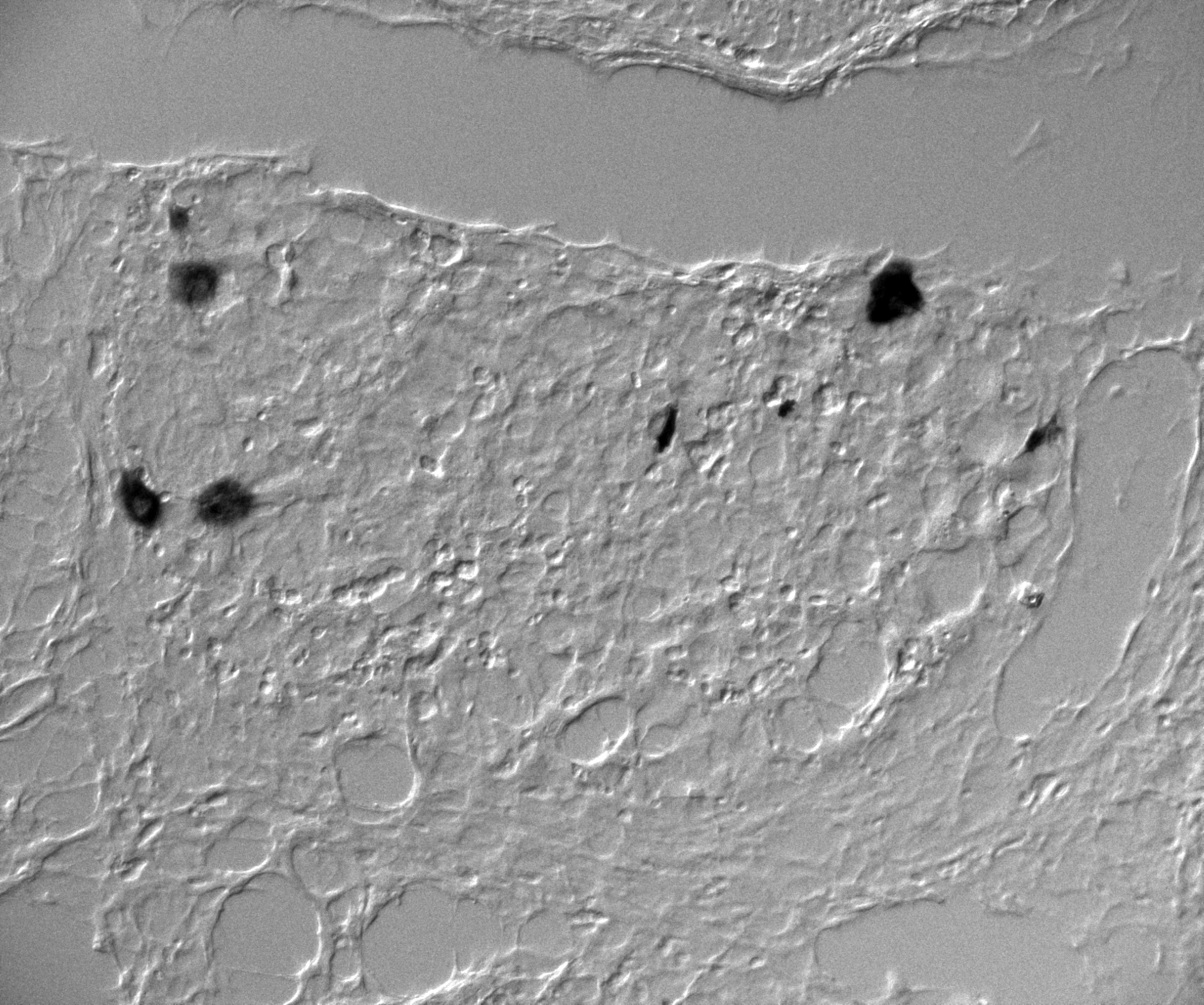

Supplement: Supplementary file 9 — Source data Fig. 4 [file 44318_2025_427_MOESM9_ESM.zip › Figure 4/4F/W1D1 E18.5 cko 32 Chodl 004.tif]

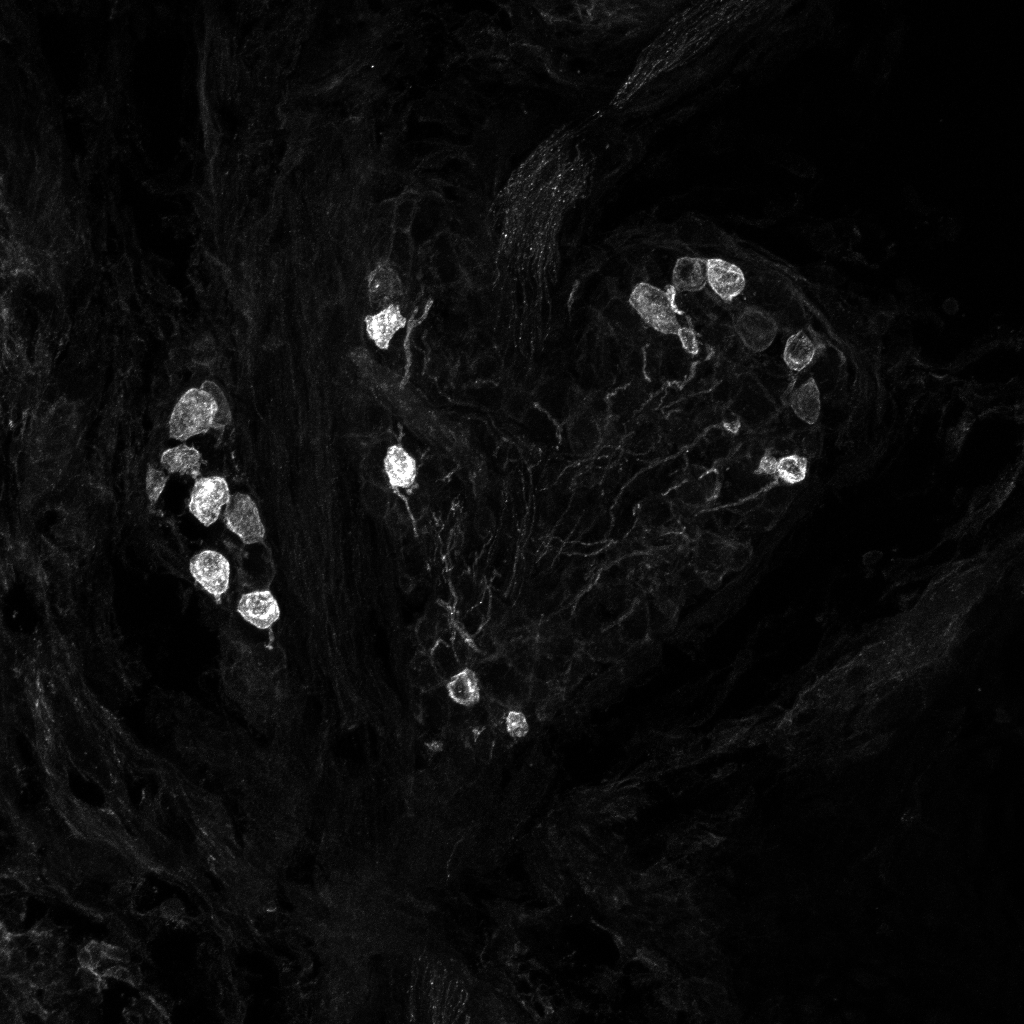

Supplement: Supplementary file 9 — Source data Fig. 4 [file 44318_2025_427_MOESM9_ESM.zip › Figure 4/4G/AD1 cKO.tif]

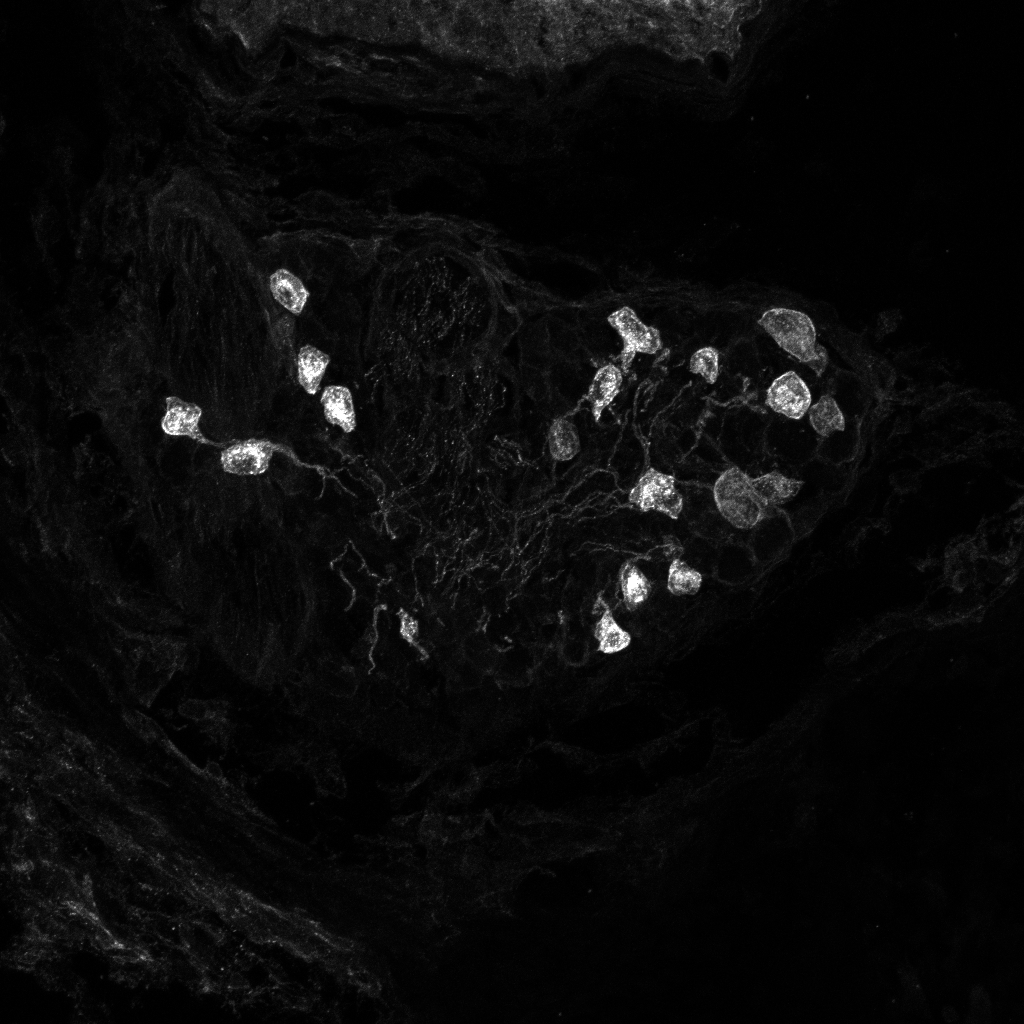

Supplement: Supplementary file 9 — Source data Fig. 4 [file 44318_2025_427_MOESM9_ESM.zip › Figure 4/4G/Ctrl.tif]

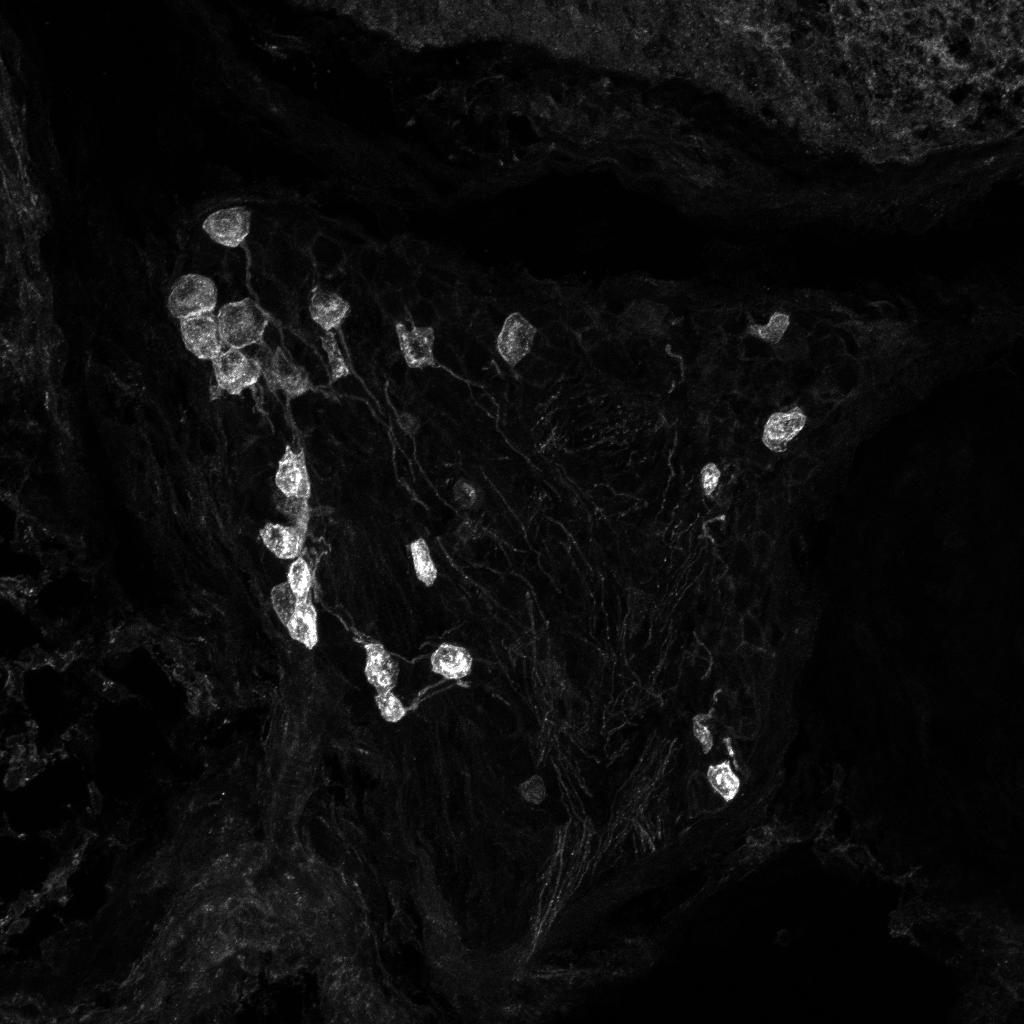

Supplement: Supplementary file 9 — Source data Fig. 4 [file 44318_2025_427_MOESM9_ESM.zip › Figure 4/4G/WD1 cKO.tif]

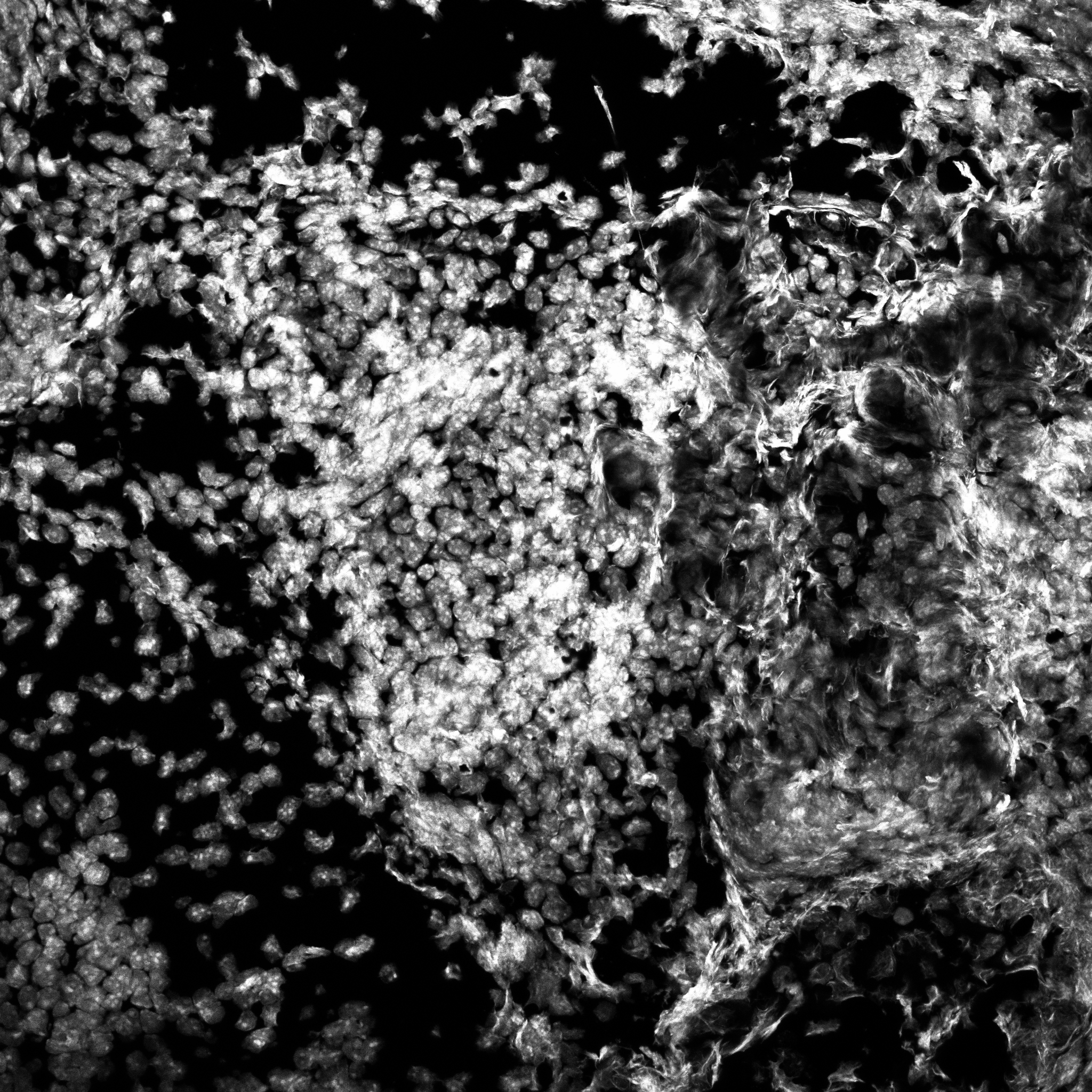

Supplement: Supplementary file 9 — Source data Fig. 4 [file 44318_2025_427_MOESM9_ESM.zip › Figure 4/4H/Control E18.5 #6 TrkB Chodl Shox2.tif]

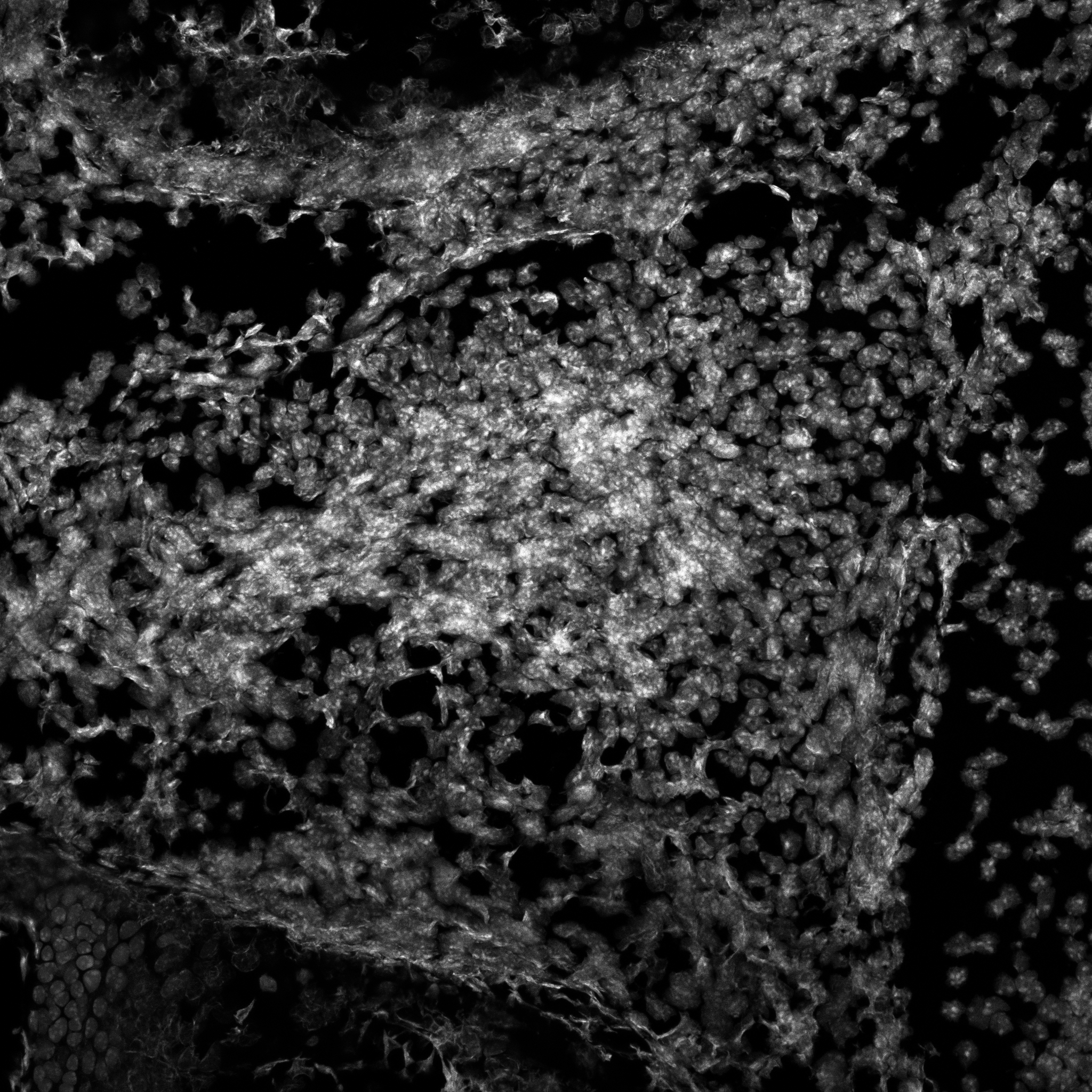

Supplement: Supplementary file 9 — Source data Fig. 4 [file 44318_2025_427_MOESM9_ESM.zip › Figure 4/4H/WD1 E18.5 #5 TrkB Chodl Shox2.tif]

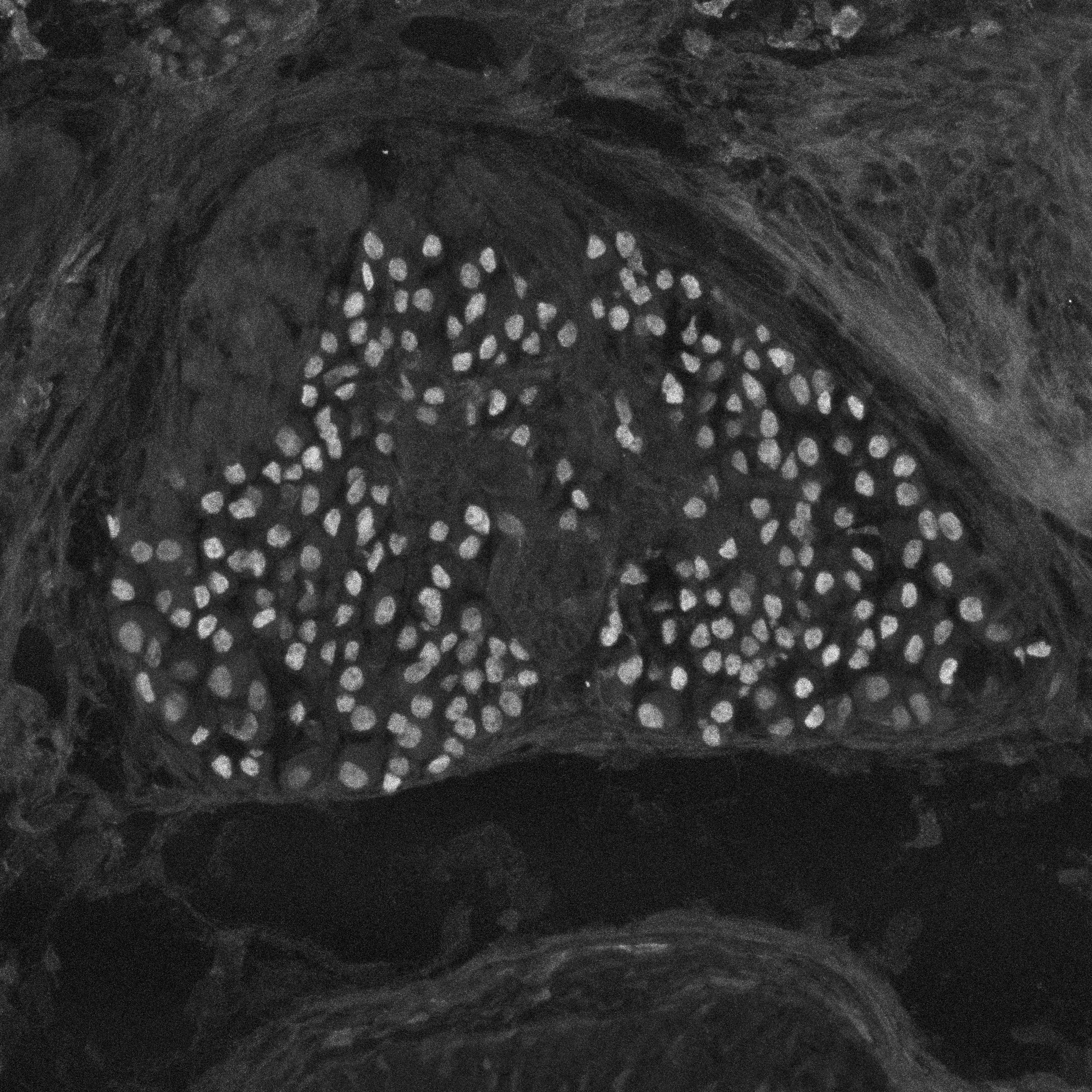

Supplement: Supplementary file 10 — Source data Fig. 5 [file 44318_2025_427_MOESM10_ESM.zip › Figure 5/5A/AD1OE Isl1 E18-5.tif]

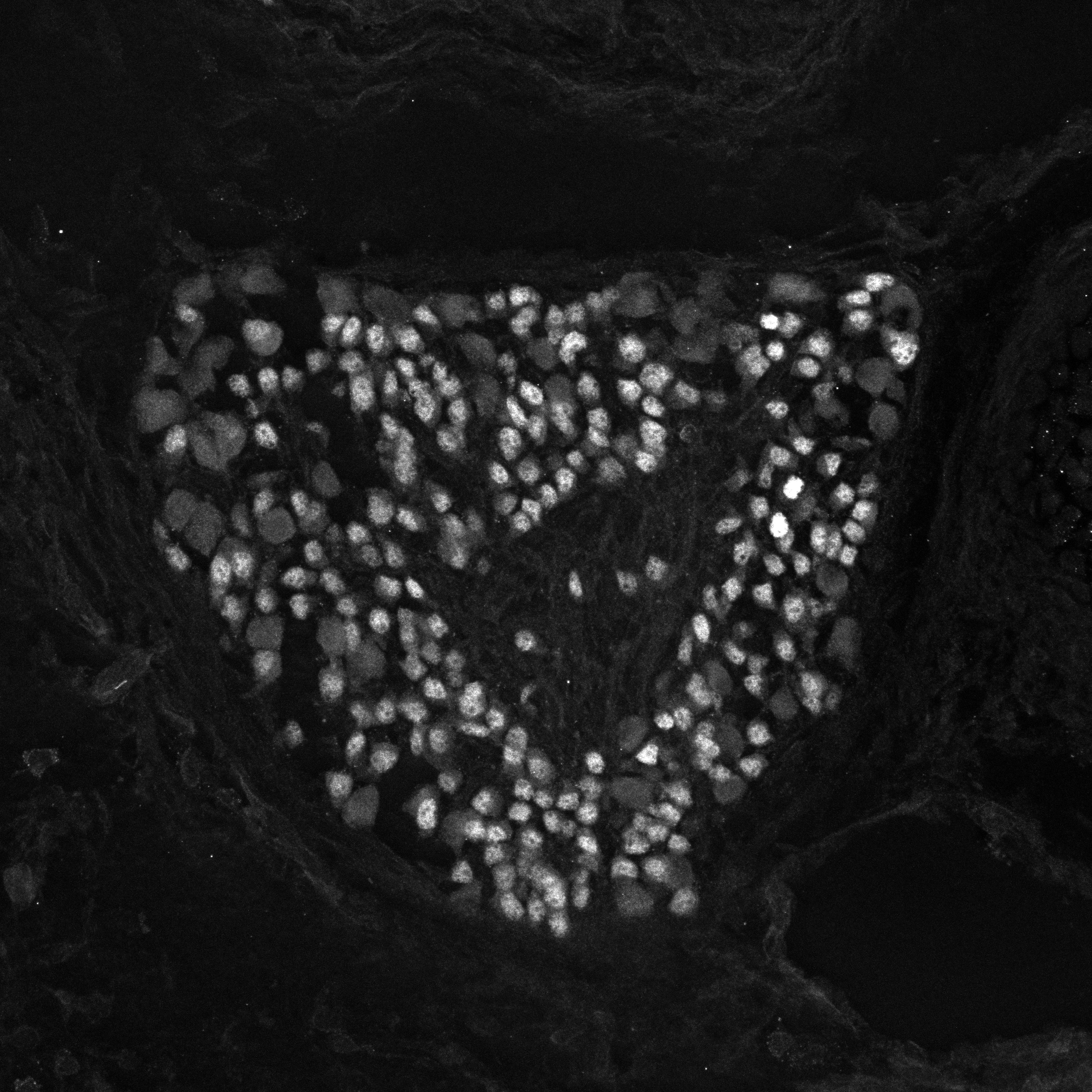

Supplement: Supplementary file 10 — Source data Fig. 5 [file 44318_2025_427_MOESM10_ESM.zip › Figure 5/5A/AD1OE-P12 E18-5.tif]

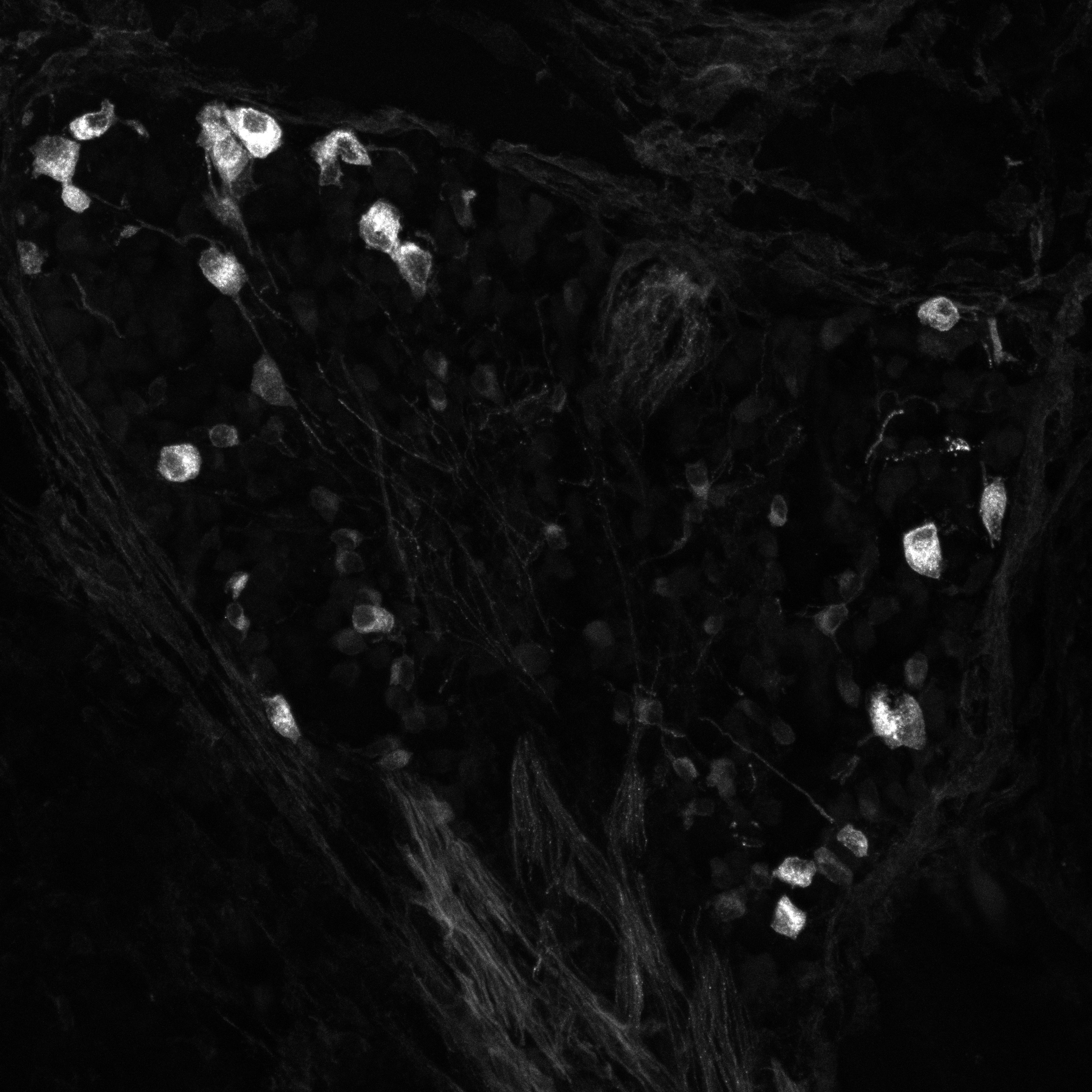

Supplement: Supplementary file 10 — Source data Fig. 5 [file 44318_2025_427_MOESM10_ESM.zip › Figure 5/5A/AD1OE-Th+trkC E18-5.tif]

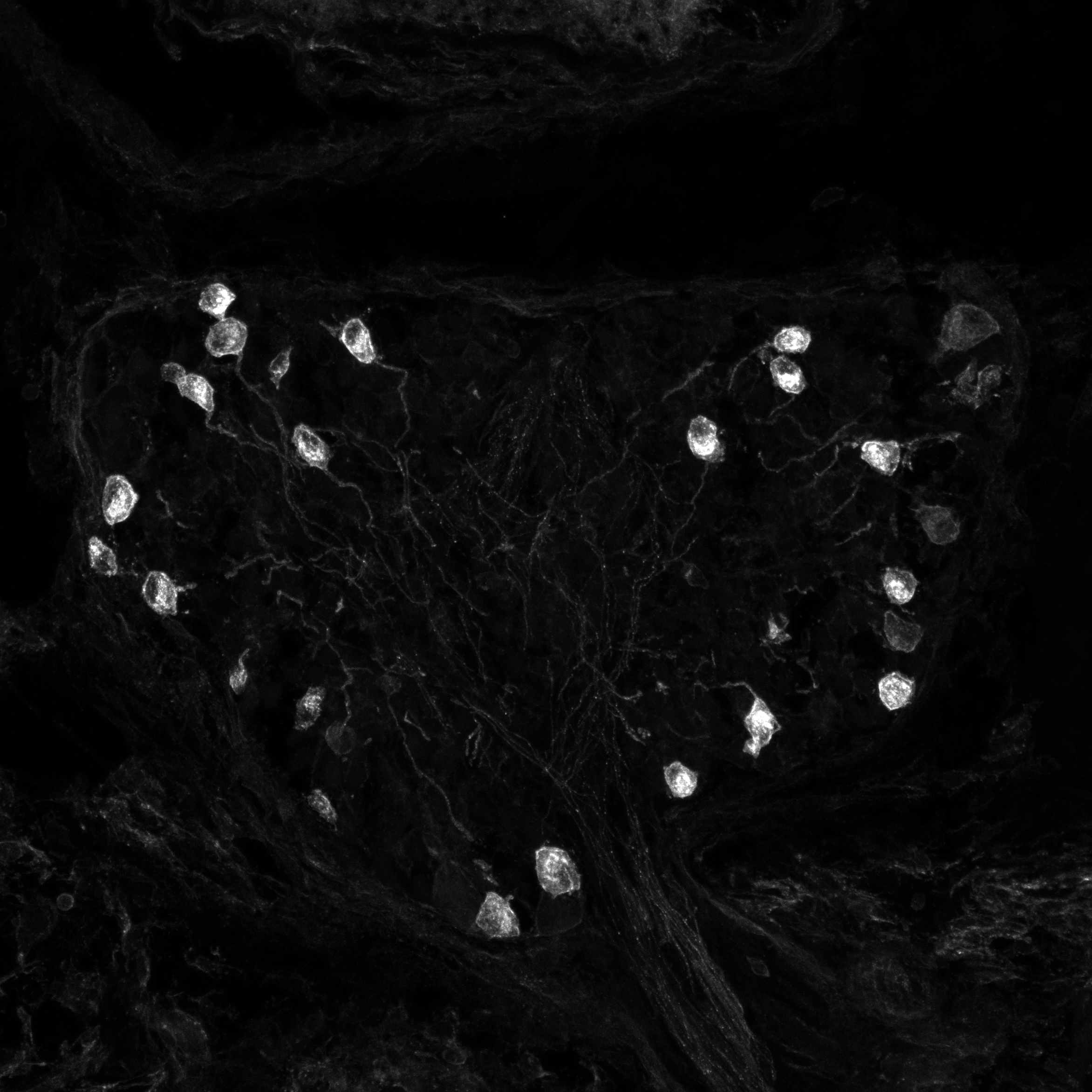

Supplement: Supplementary file 10 — Source data Fig. 5 [file 44318_2025_427_MOESM10_ESM.zip › Figure 5/5A/AD1OE-trkB E18-5.tif]

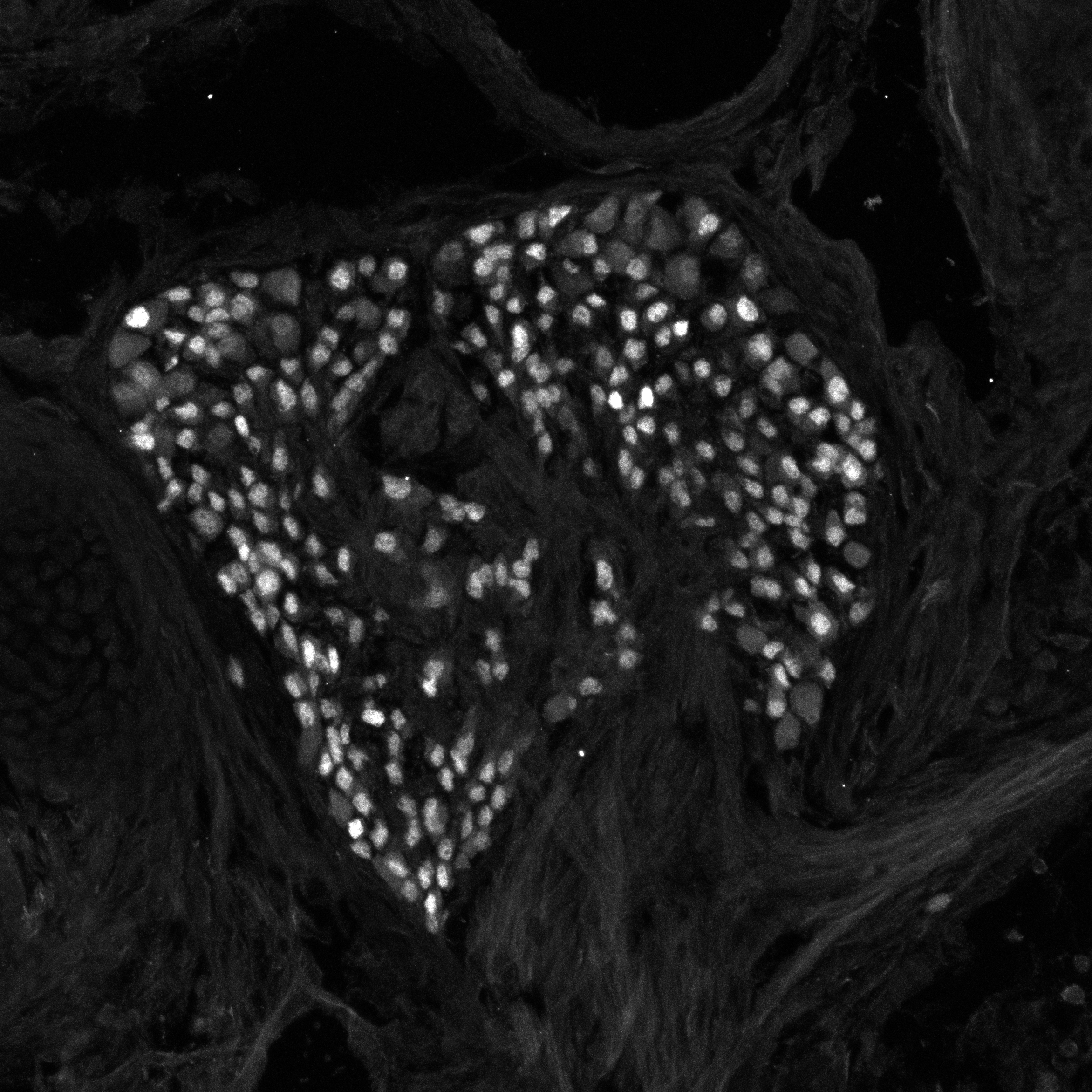

Supplement: Supplementary file 10 — Source data Fig. 5 [file 44318_2025_427_MOESM10_ESM.zip › Figure 5/5A/Ctl-P12-E18-5.tif]

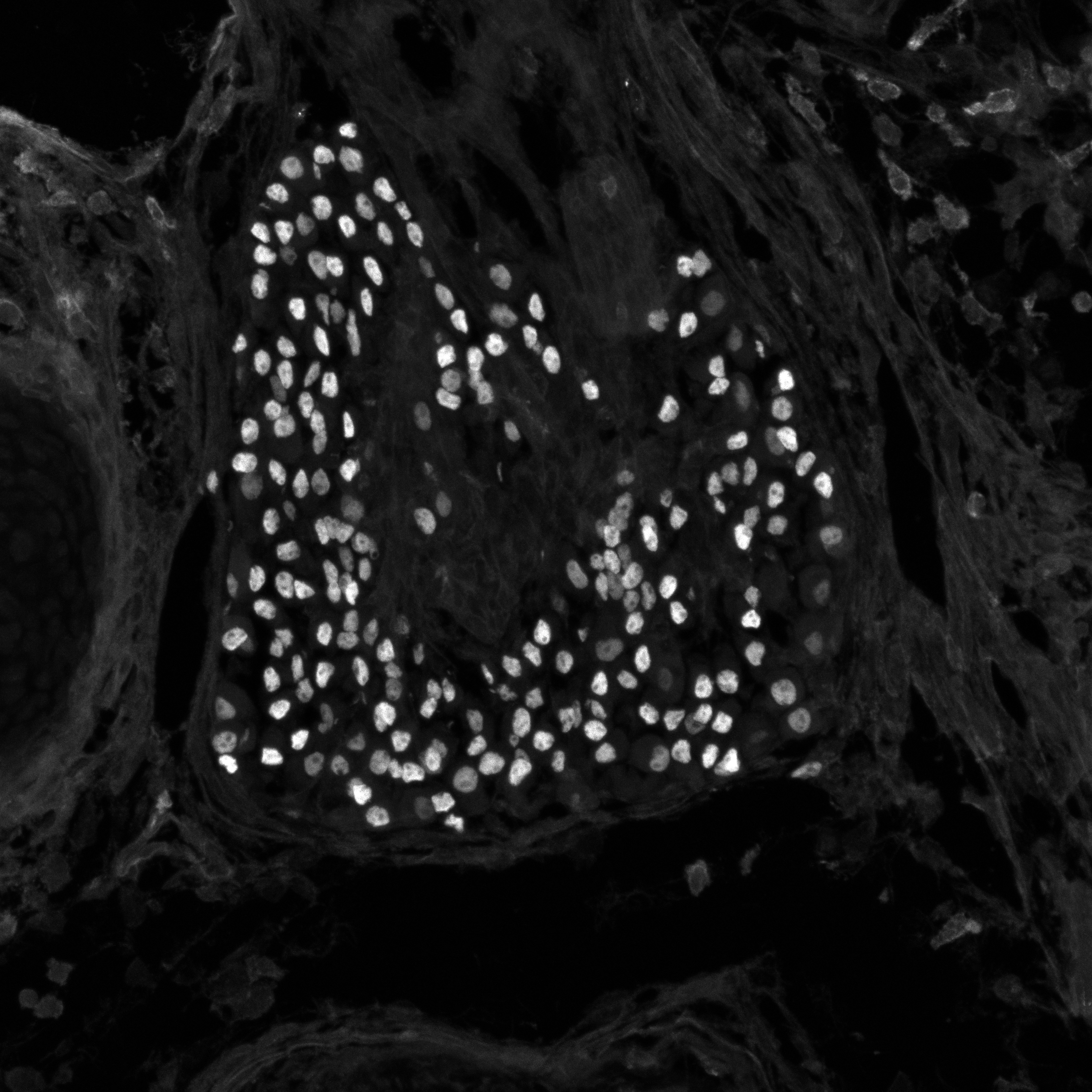

Supplement: Supplementary file 10 — Source data Fig. 5 [file 44318_2025_427_MOESM10_ESM.zip › Figure 5/5A/Ctrl-Isl1-E18.5.tif]

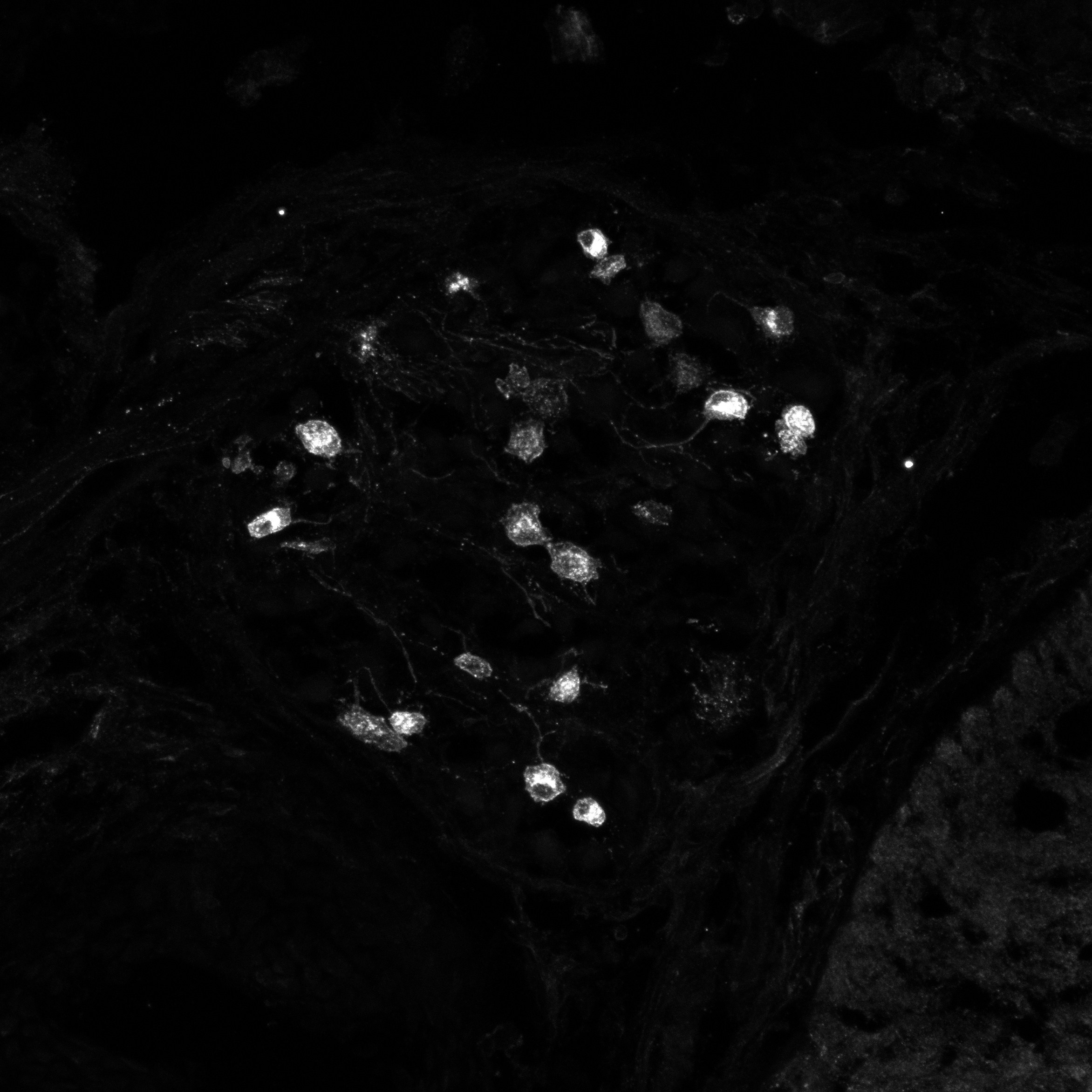

Supplement: Supplementary file 10 — Source data Fig. 5 [file 44318_2025_427_MOESM10_ESM.zip › Figure 5/5A/Ctrl-TrkB-E18-5]

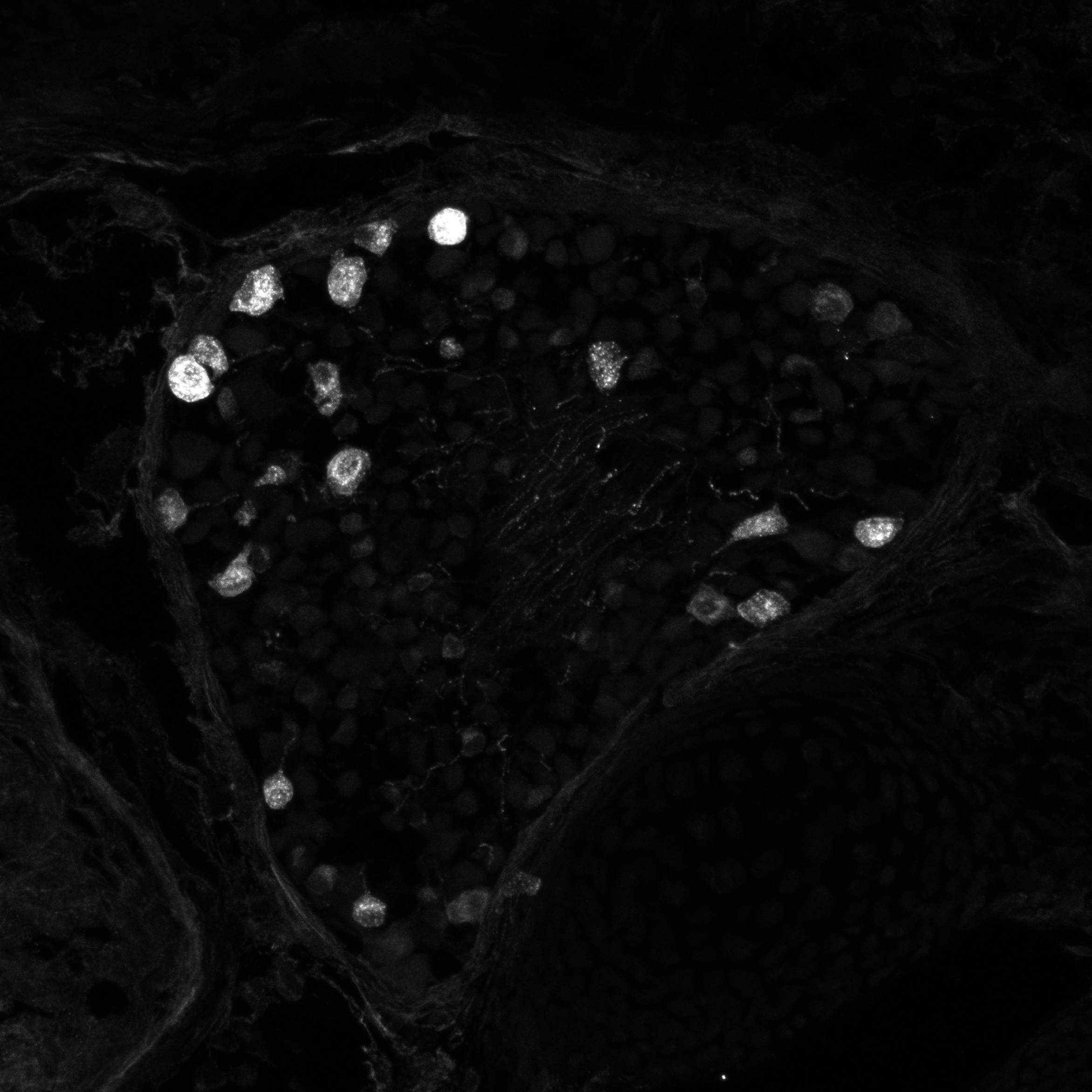

Supplement: Supplementary file 10 — Source data Fig. 5 [file 44318_2025_427_MOESM10_ESM.zip › Figure 5/5A/Ctrl-TrkC-E18.5.tif]

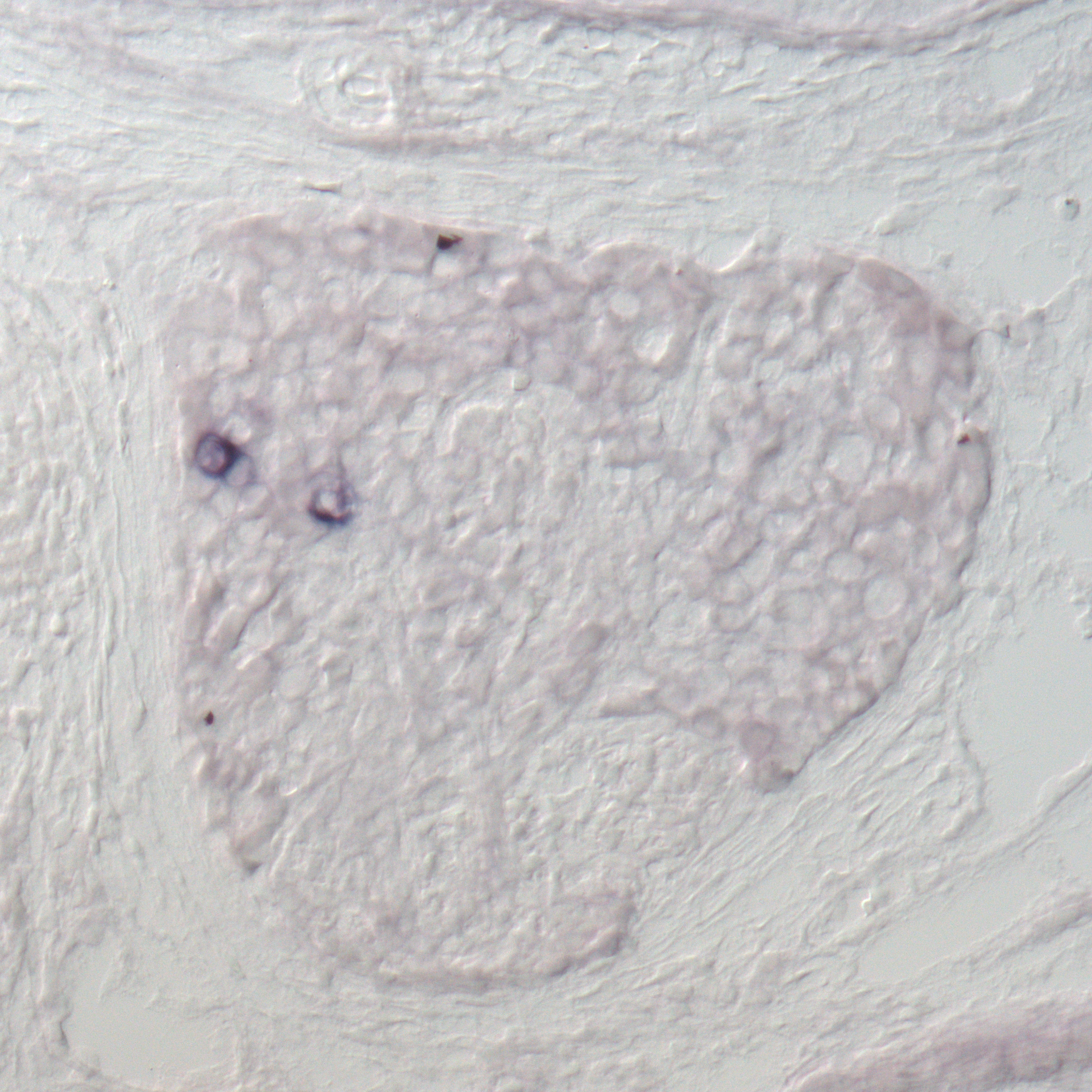

Supplement: Supplementary file 10 — Source data Fig. 5 [file 44318_2025_427_MOESM10_ESM.zip › Figure 5/5G/AD1OE-TrpM8-E18-5.tif]

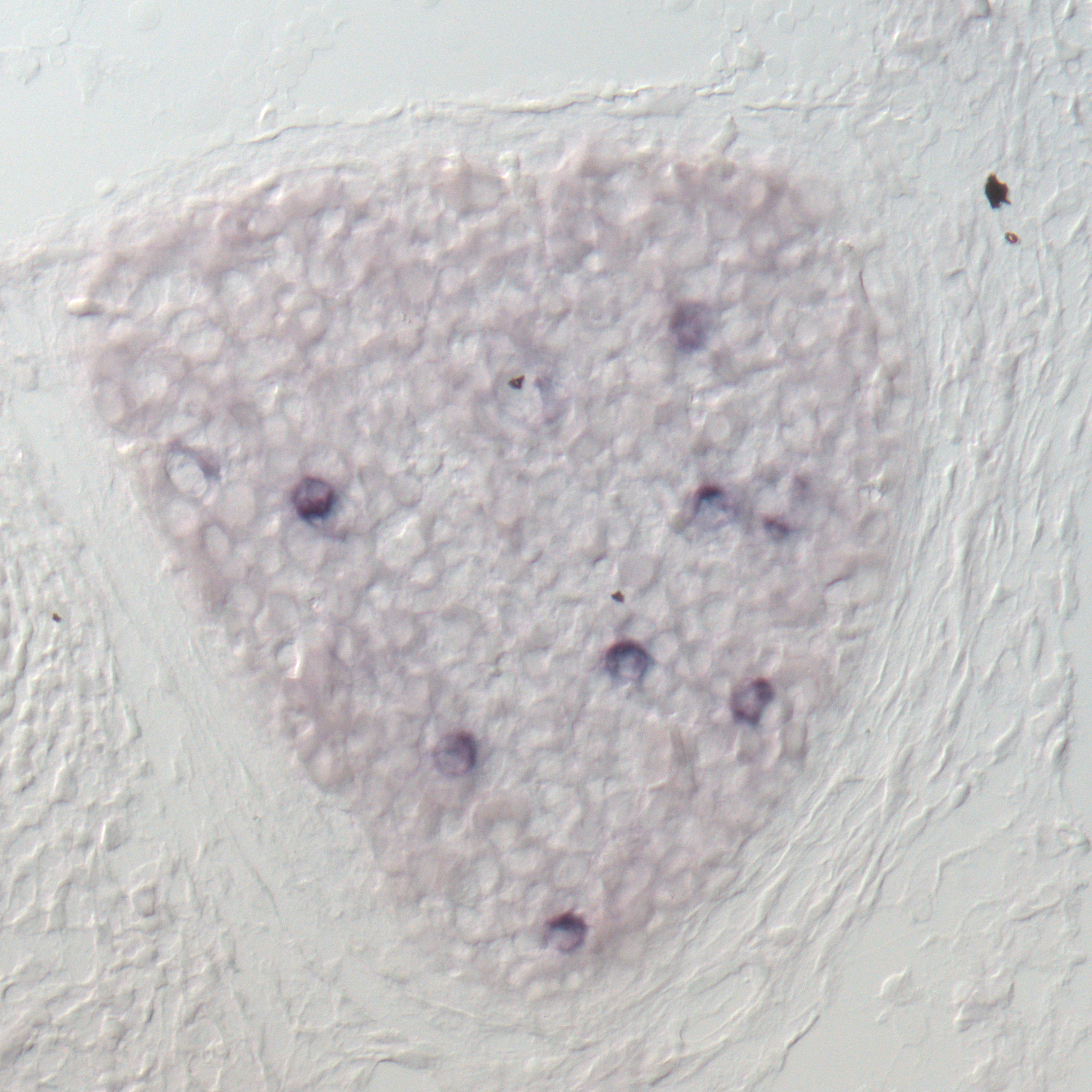

Supplement: Supplementary file 10 — Source data Fig. 5 [file 44318_2025_427_MOESM10_ESM.zip › Figure 5/5G/Ctrl-TrpM8-E18-5.tif]

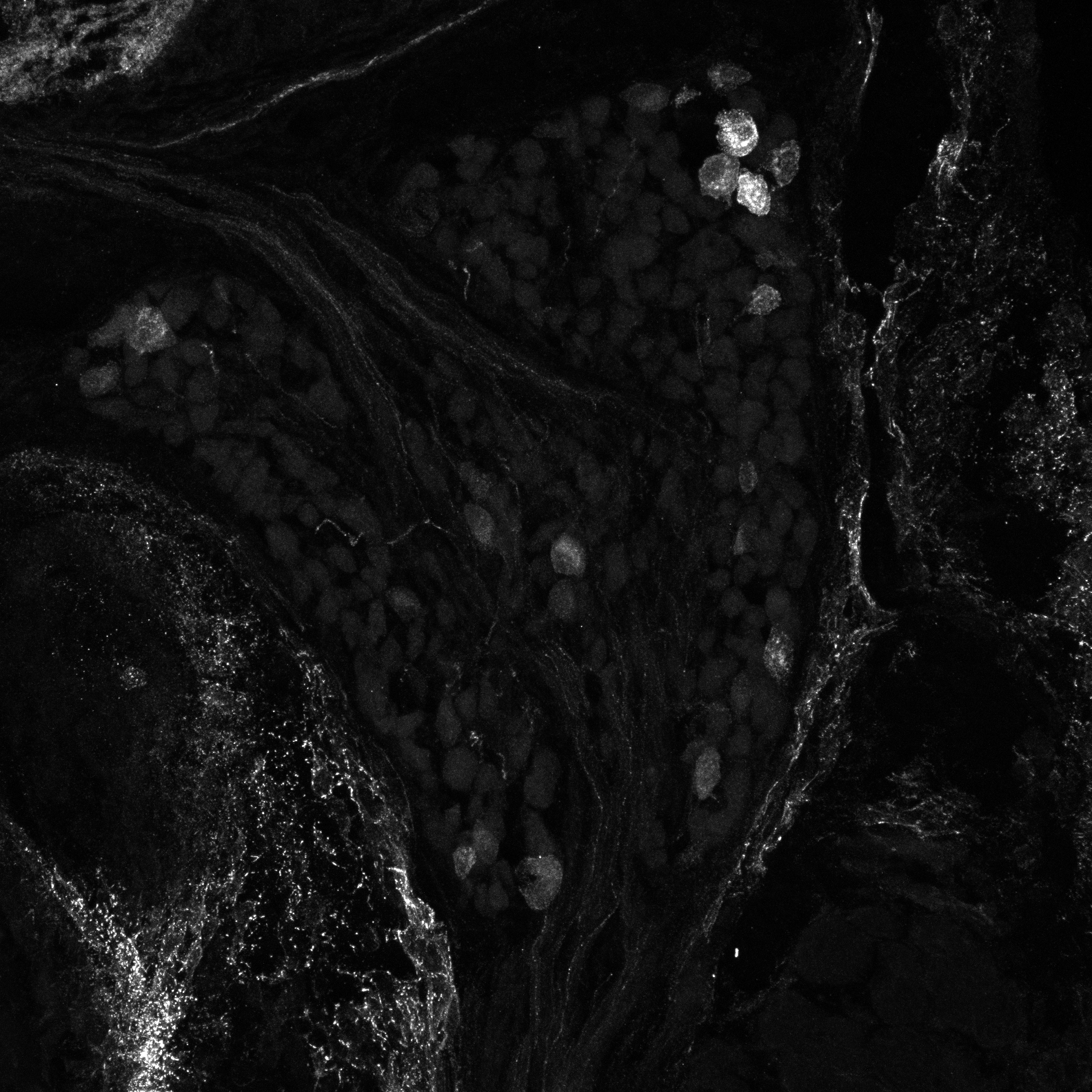

Supplement: Supplementary file 10 — Source data Fig. 5 [file 44318_2025_427_MOESM10_ESM.zip › Figure 5/5G/MAX_AD1OE-cdh3-e18-5.tif]

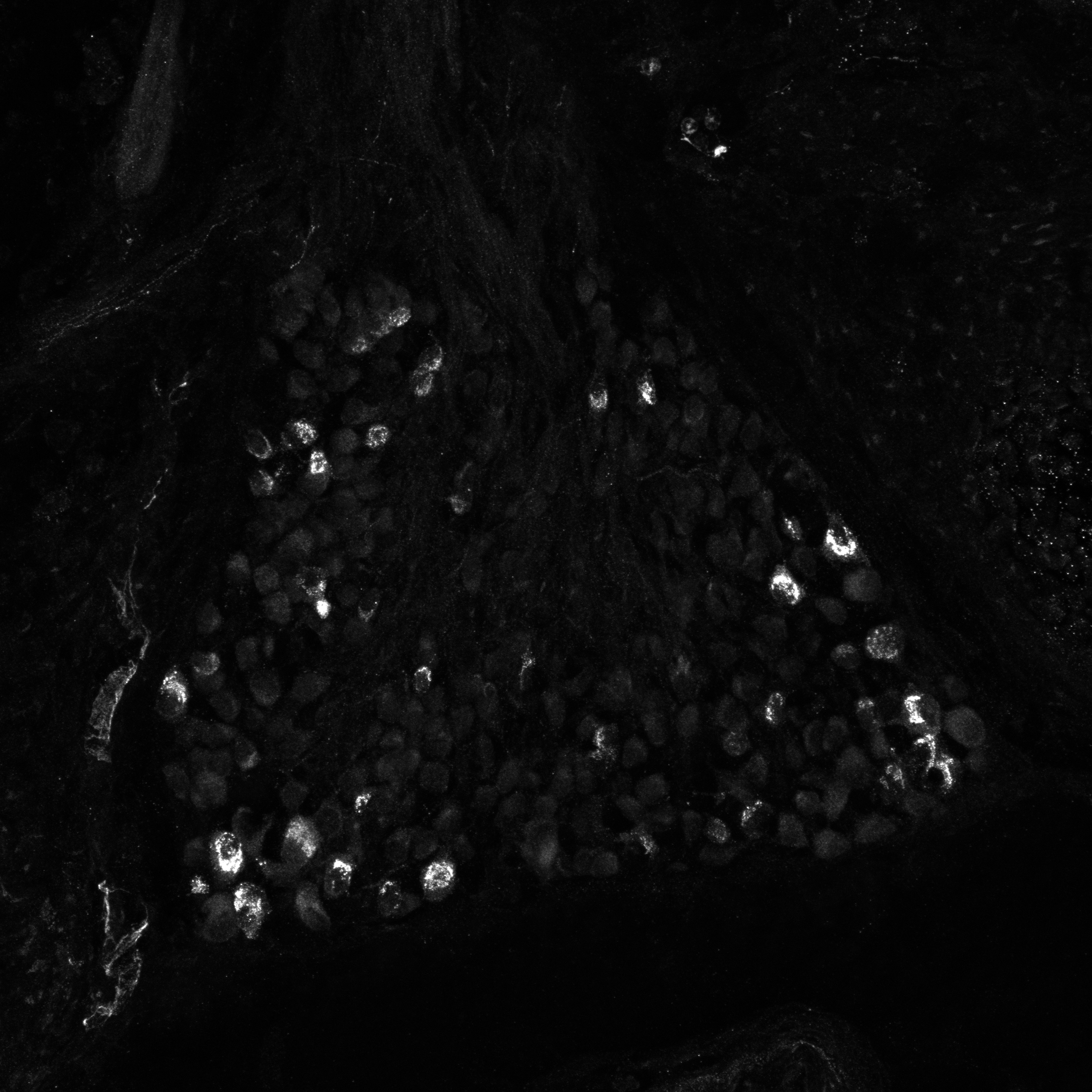

Supplement: Supplementary file 10 — Source data Fig. 5 [file 44318_2025_427_MOESM10_ESM.zip › Figure 5/5G/MAX_AD1OE-CGRP E18-5.tif]

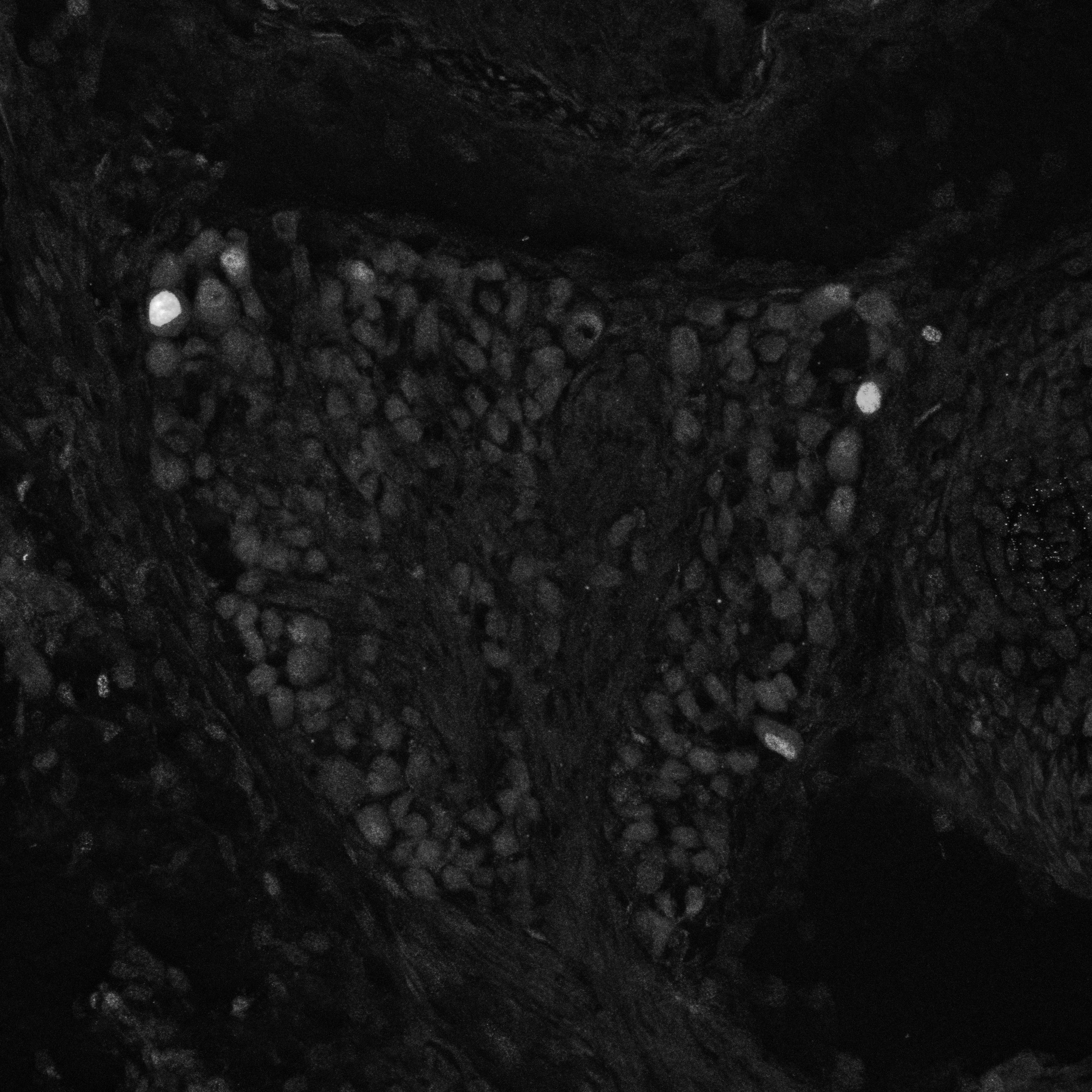

Supplement: Supplementary file 10 — Source data Fig. 5 [file 44318_2025_427_MOESM10_ESM.zip › Figure 5/5G/MAX_AD1OE-MafA E18-5.tif]

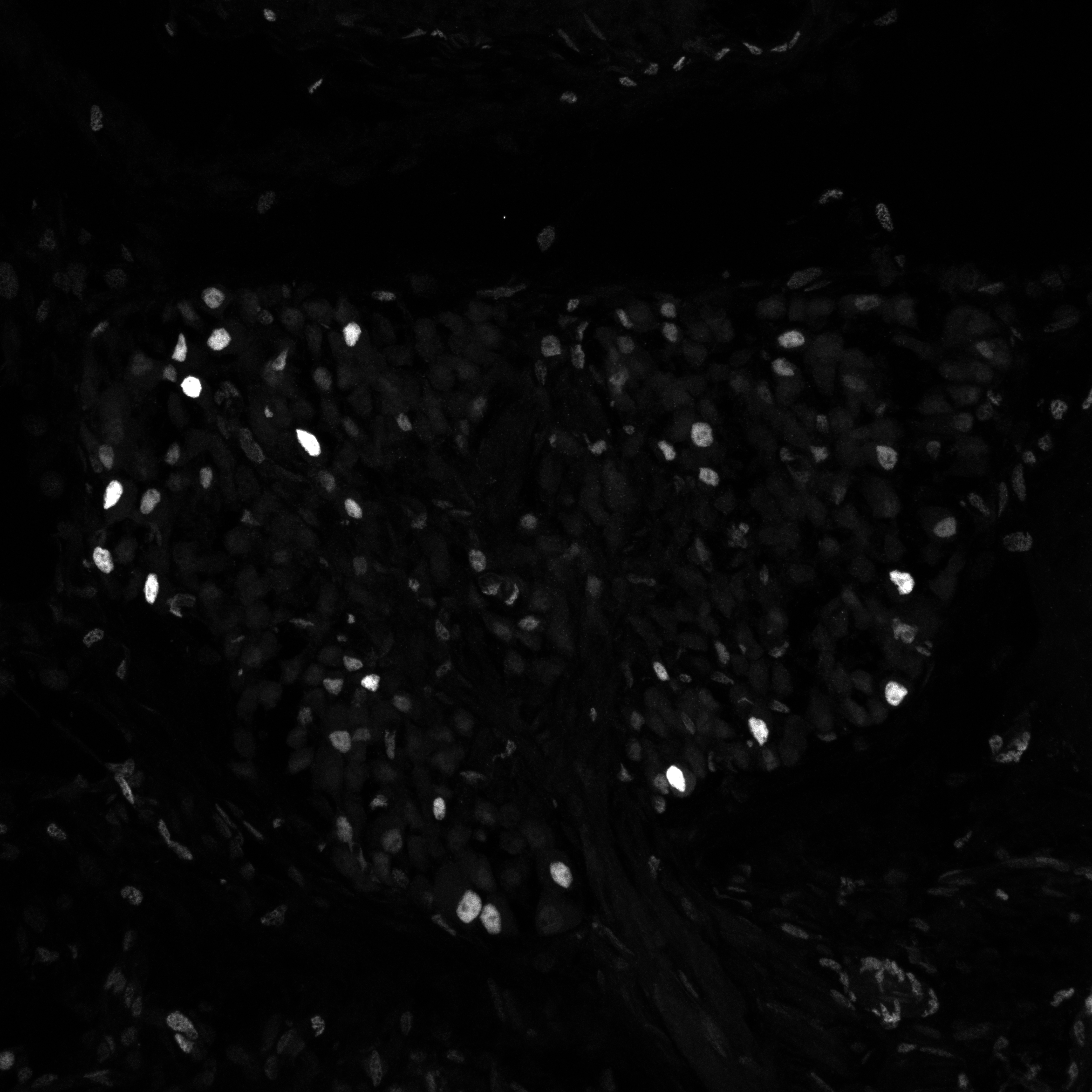

Supplement: Supplementary file 10 — Source data Fig. 5 [file 44318_2025_427_MOESM10_ESM.zip › Figure 5/5G/MAX_AD1OE-mef2c E18-5.tif]

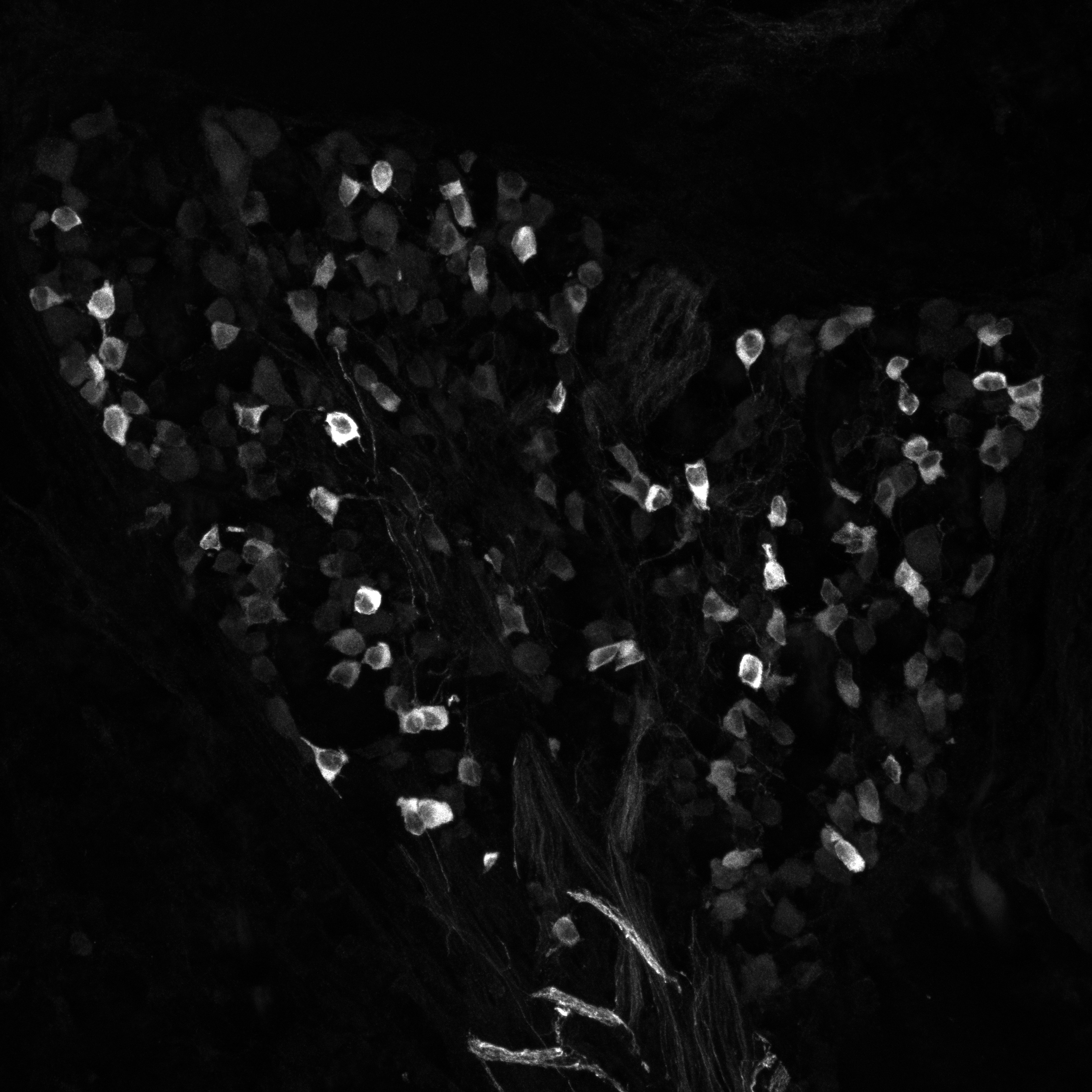

Supplement: Supplementary file 10 — Source data Fig. 5 [file 44318_2025_427_MOESM10_ESM.zip › Figure 5/5G/MAX_AD1OE-Th E18-5-2.tif]

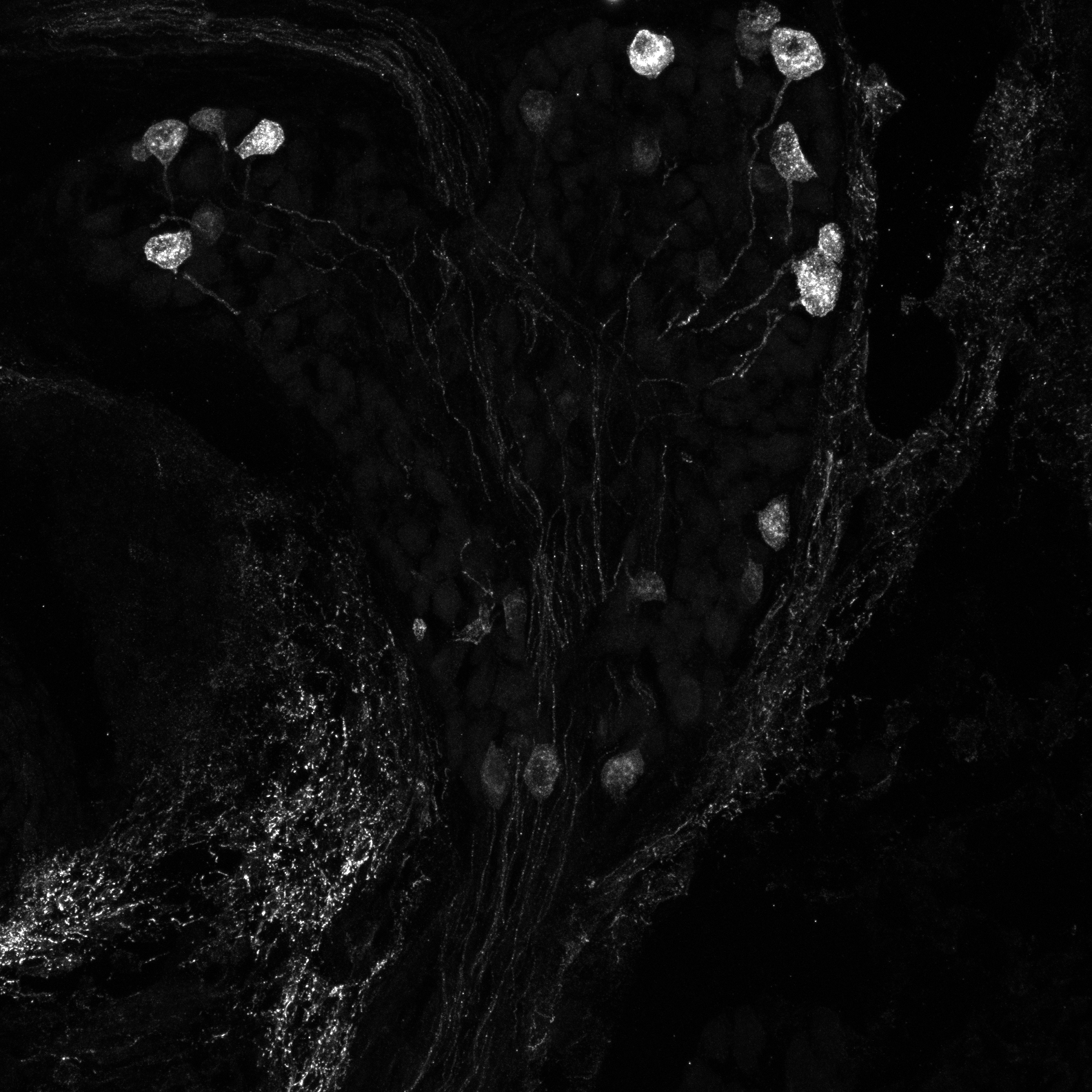

Supplement: Supplementary file 10 — Source data Fig. 5 [file 44318_2025_427_MOESM10_ESM.zip › Figure 5/5G/MAX_Ctrl-cdh3-e18-5.tif]

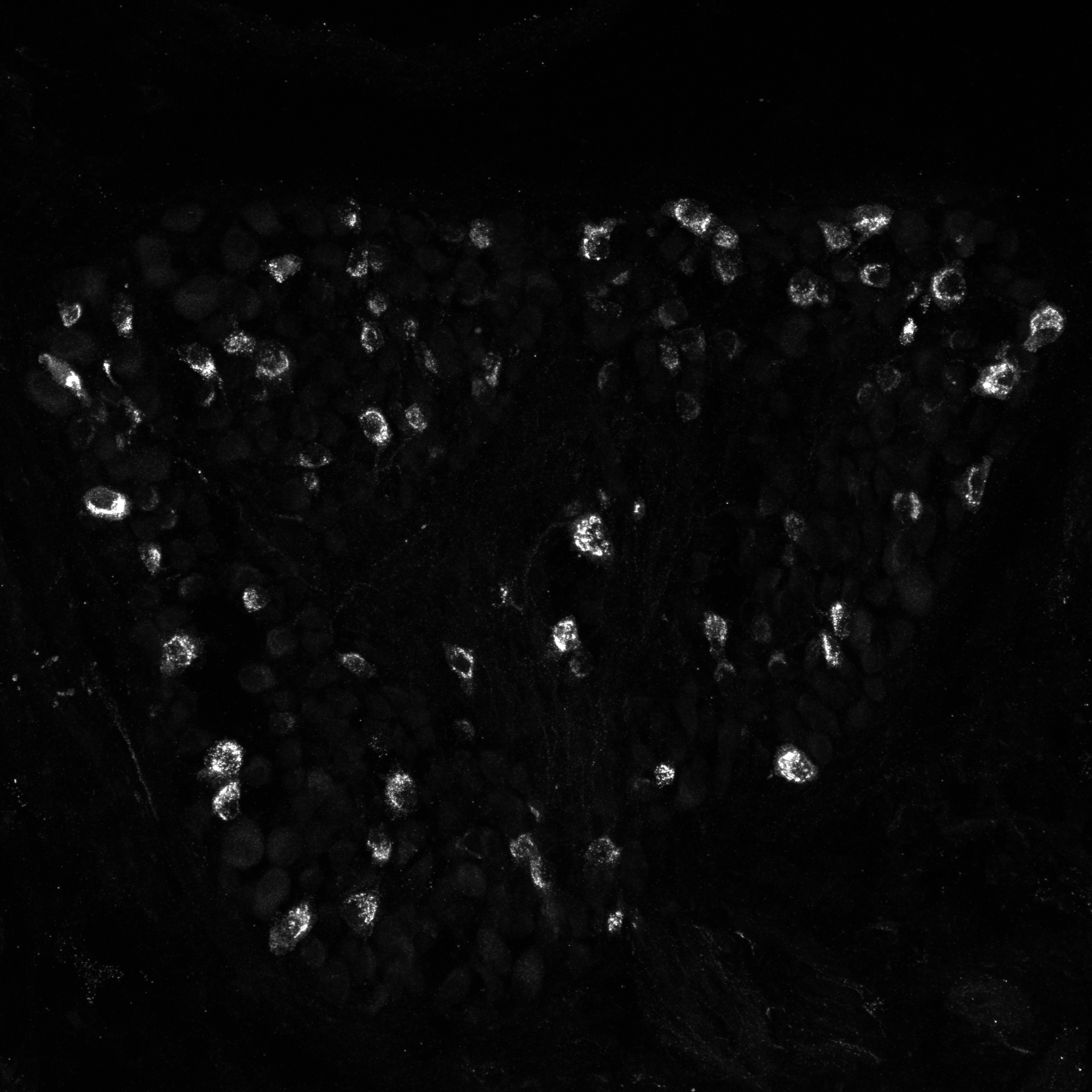

Supplement: Supplementary file 10 — Source data Fig. 5 [file 44318_2025_427_MOESM10_ESM.zip › Figure 5/5G/MAX_Ctrl-CGRP E18-5.tif]

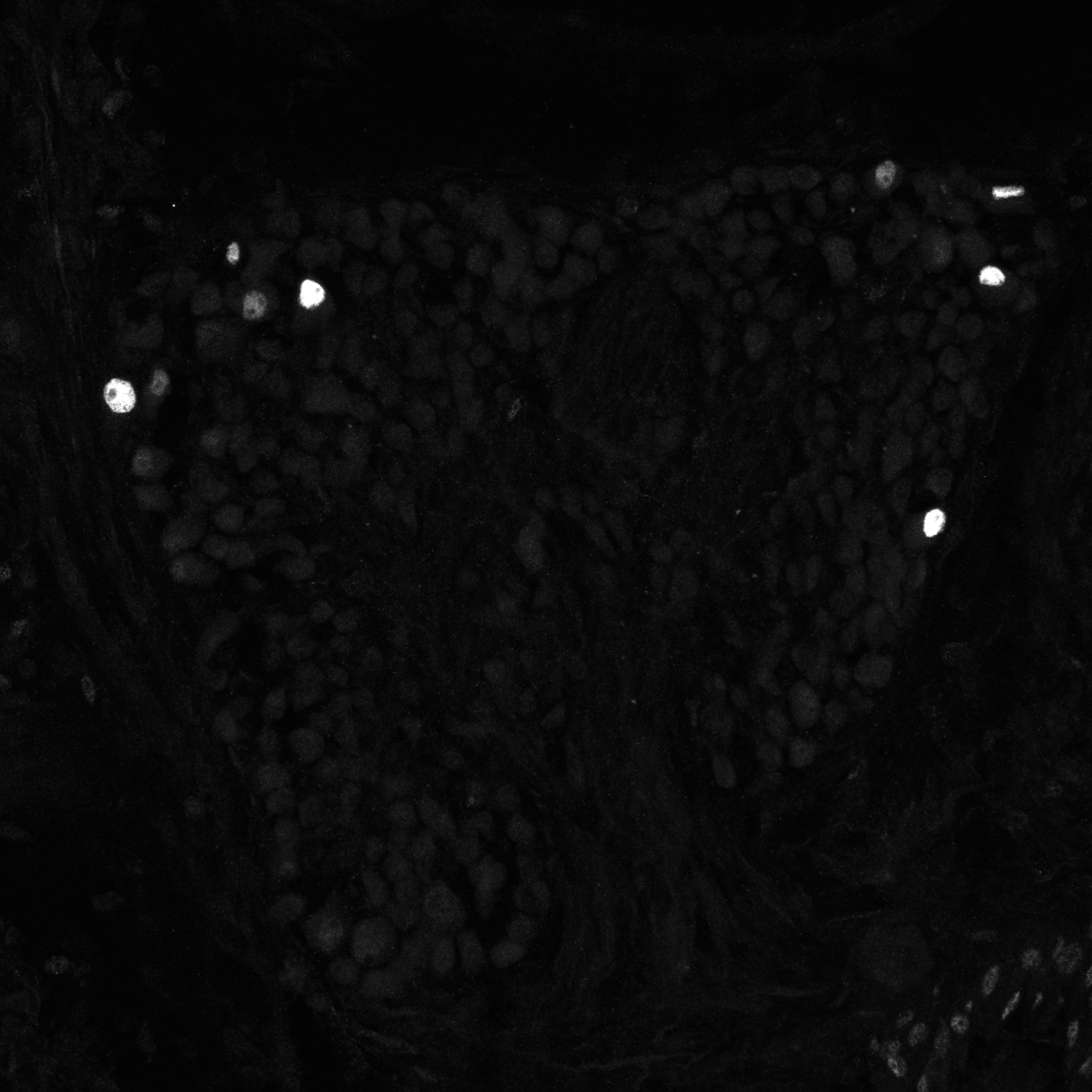

Supplement: Supplementary file 10 — Source data Fig. 5 [file 44318_2025_427_MOESM10_ESM.zip › Figure 5/5G/MAX_Ctrl-MafA E18-5.tif]

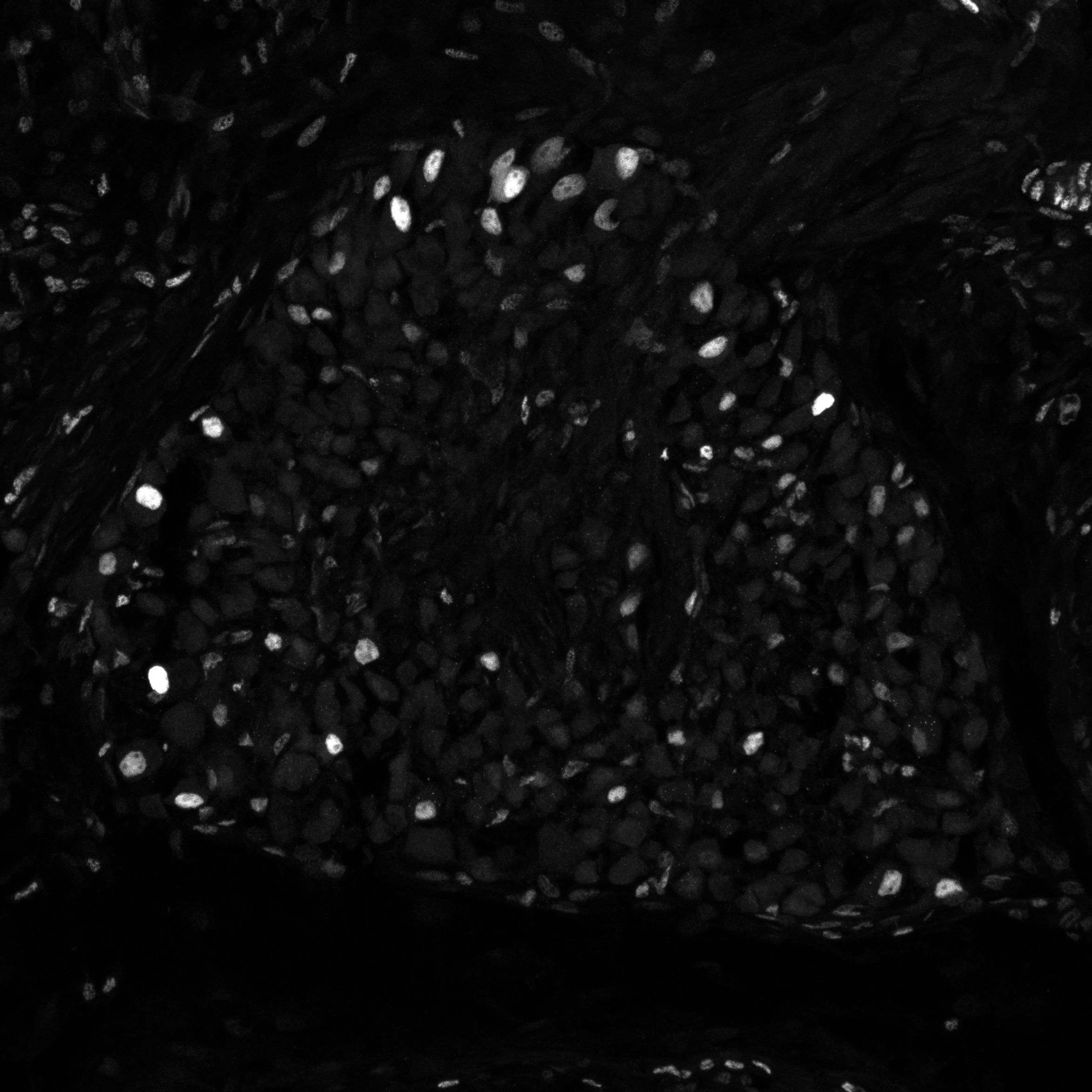

Supplement: Supplementary file 10 — Source data Fig. 5 [file 44318_2025_427_MOESM10_ESM.zip › Figure 5/5G/MAX_Ctrl-mef2c E18-5.tif]

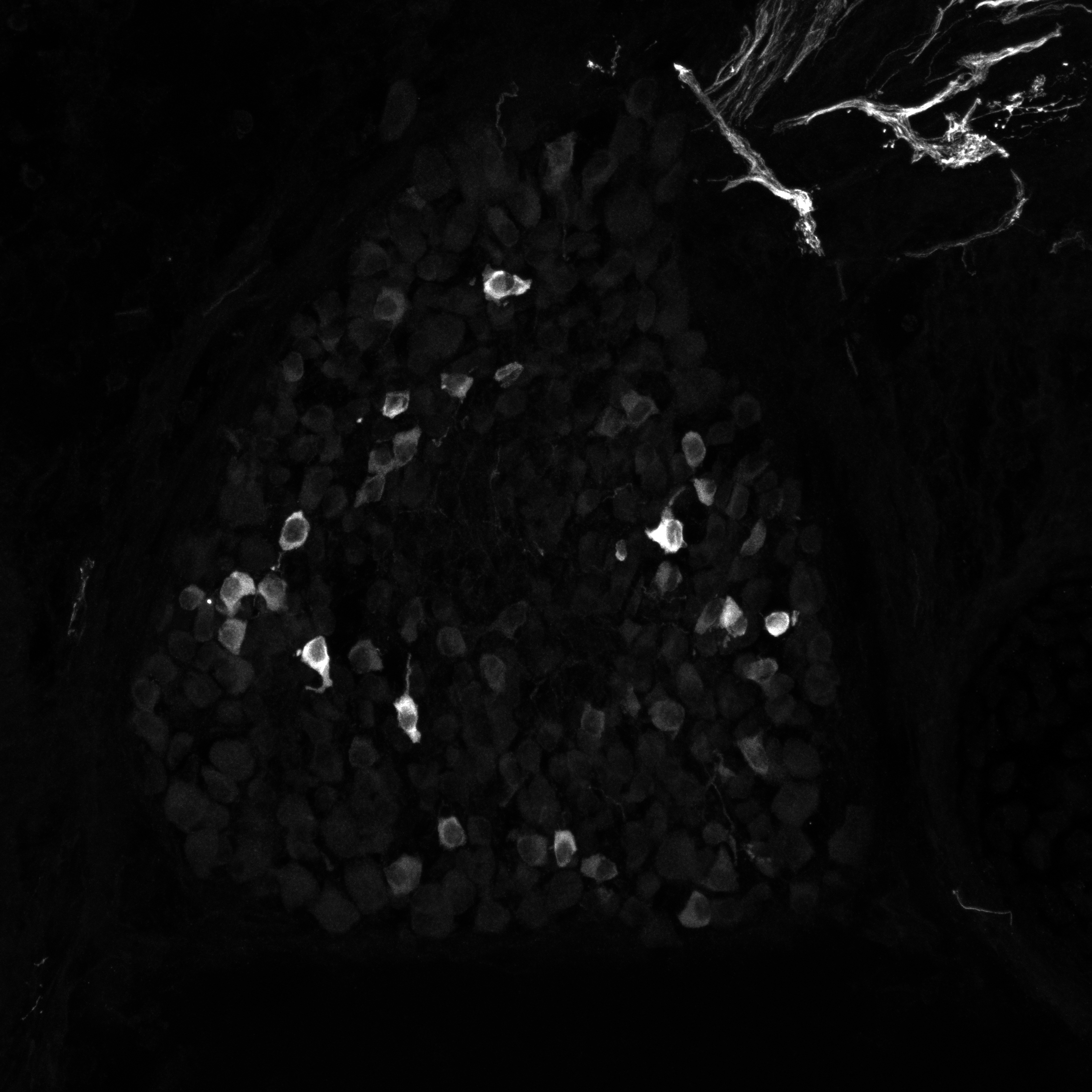

Supplement: Supplementary file 10 — Source data Fig. 5 [file 44318_2025_427_MOESM10_ESM.zip › Figure 5/5G/MAX_Ctrl-Th E18-5.tif]

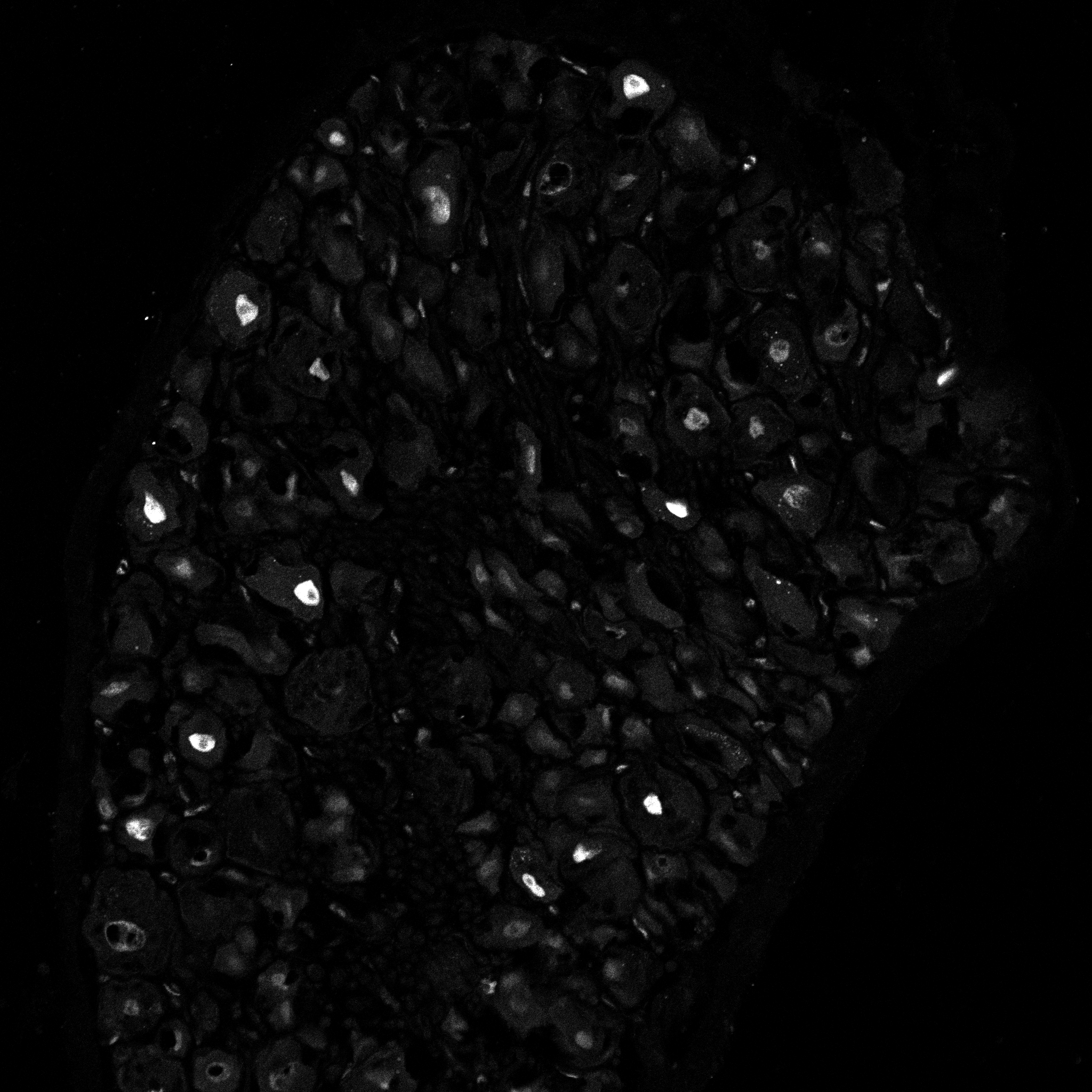

Supplement: Supplementary file 10 — Source data Fig. 5 [file 44318_2025_427_MOESM10_ESM.zip › Figure 5/5H/Ctrl - DRG IF Dach1 + DIC.tif]

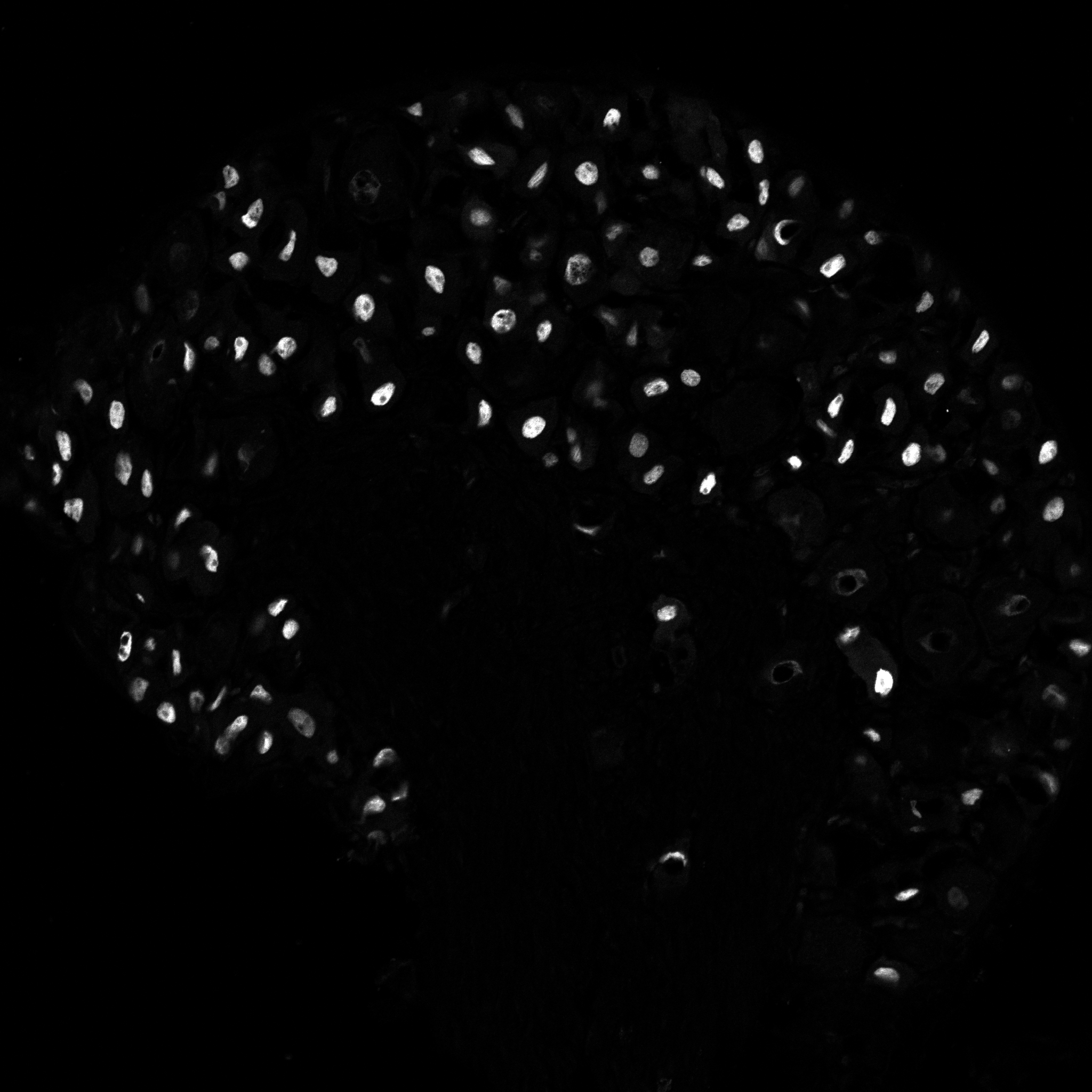

Supplement: Supplementary file 10 — Source data Fig. 5 [file 44318_2025_427_MOESM10_ESM.zip › Figure 5/5H/ND1OE- DRG IF Dach1 + DIC.tif]

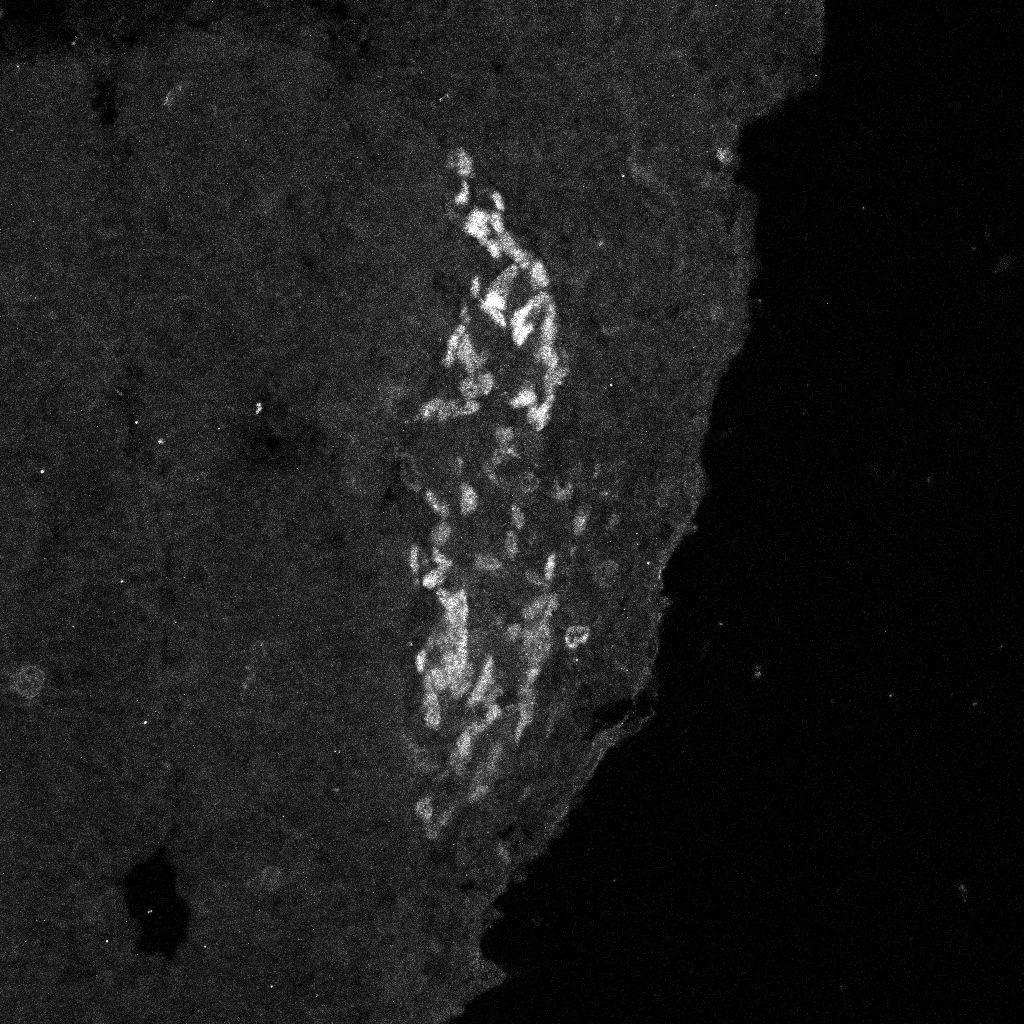

Supplement: Supplementary file 11 — Figure EV1 Source Data [file 44318_2025_427_MOESM11_ESM.zip › Figure EV1/EV1A/E10-5-Dach1 green + Sox10 red.tif]

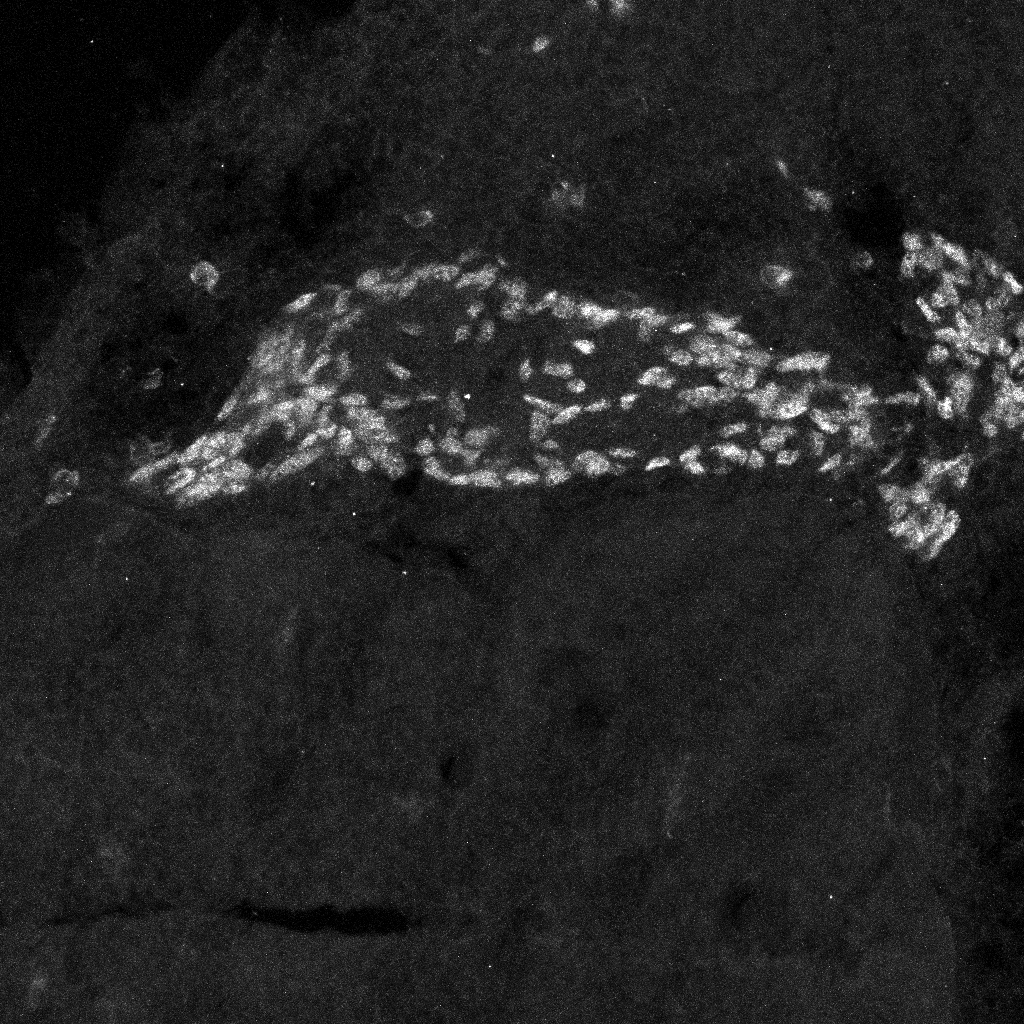

Supplement: Supplementary file 11 — Figure EV1 Source Data [file 44318_2025_427_MOESM11_ESM.zip › Figure EV1/EV1A/E11-5-Dach1 green + Sox10 red.tif]

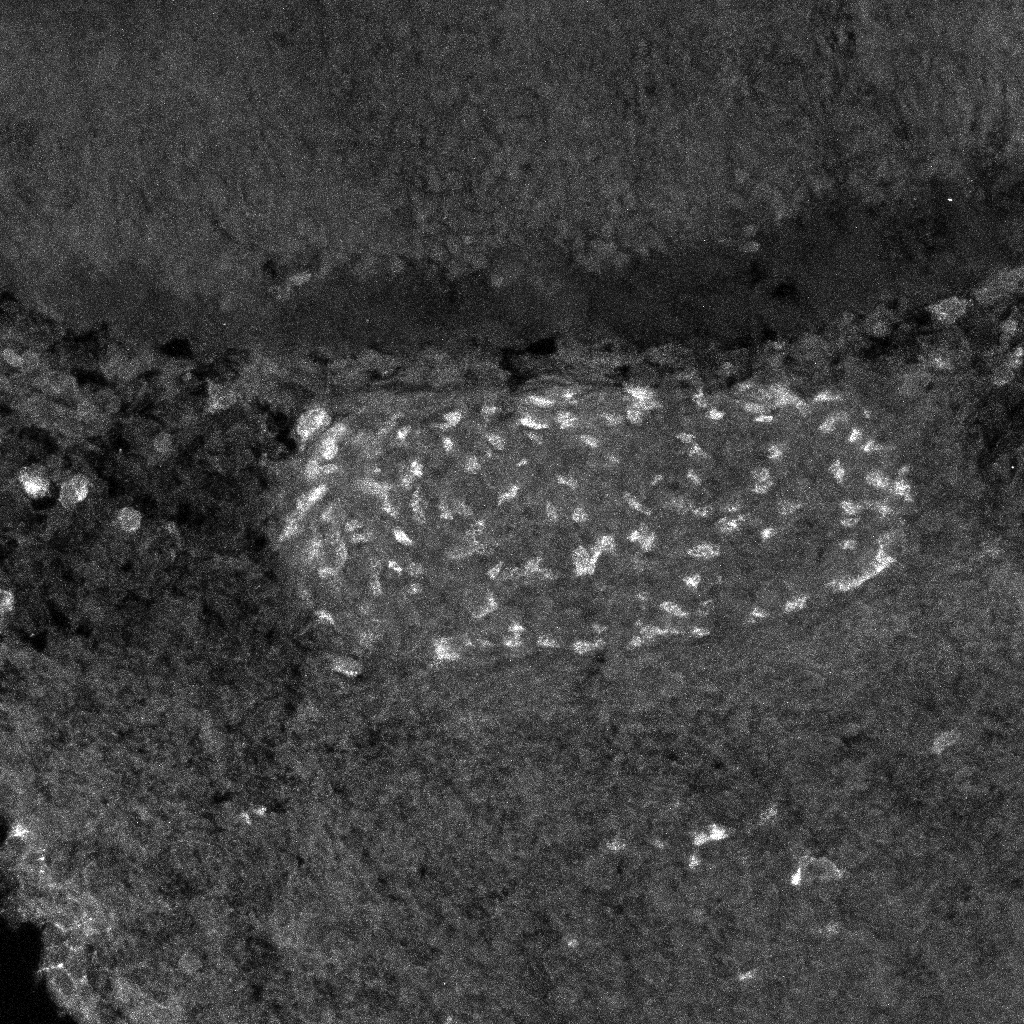

Supplement: Supplementary file 11 — Figure EV1 Source Data [file 44318_2025_427_MOESM11_ESM.zip › Figure EV1/EV1A/E12-5-Dach1 green + Sox10 red.tif]

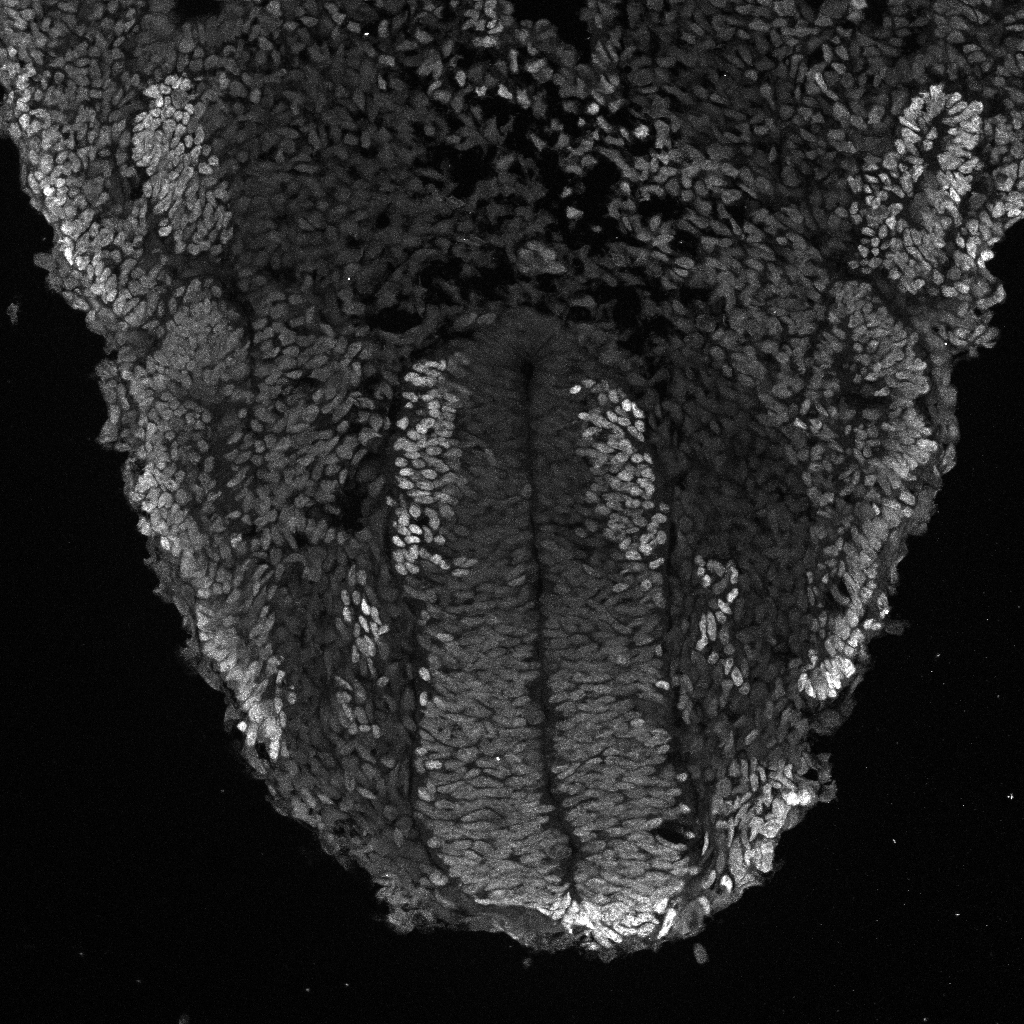

Supplement: Supplementary file 11 — Figure EV1 Source Data [file 44318_2025_427_MOESM11_ESM.zip › Figure EV1/EV1B/E10-5-BrdU red+Sox10 green+Dach1 blue.tif]

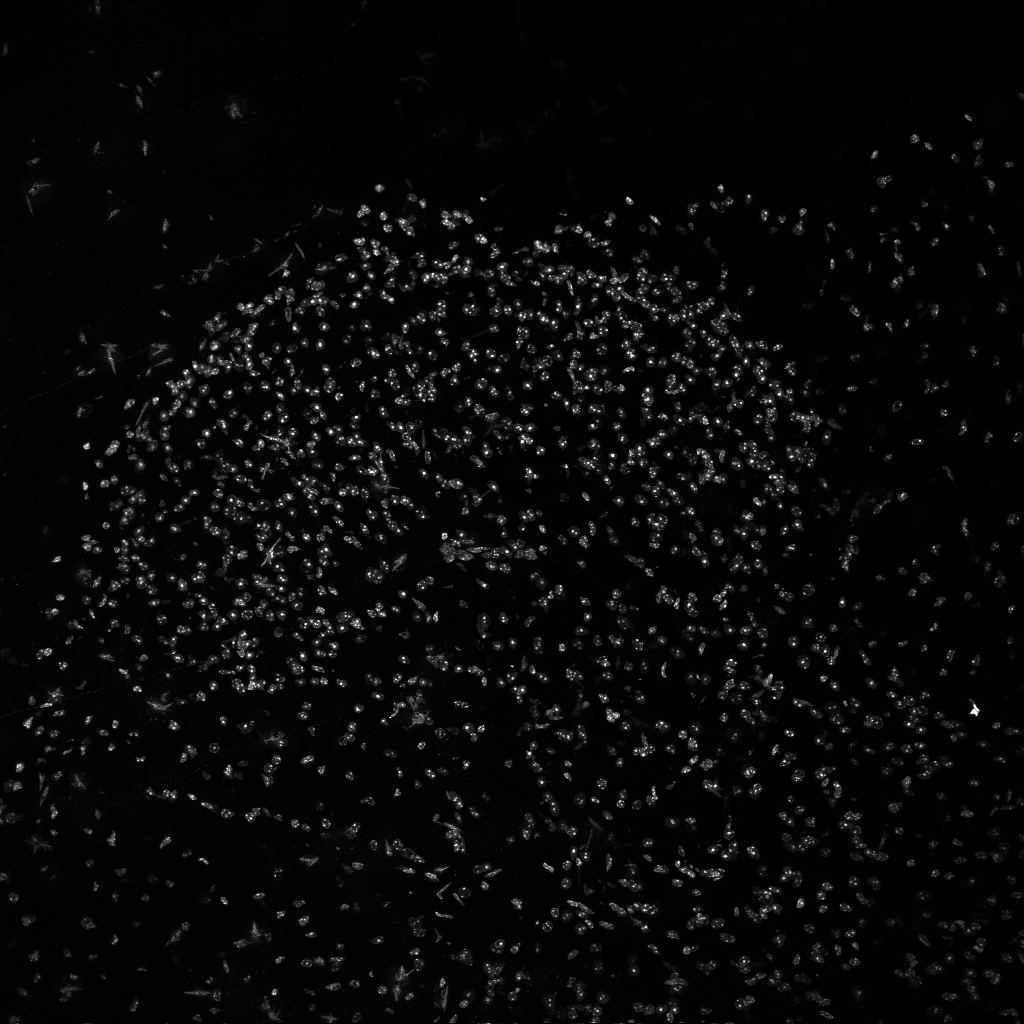

Supplement: Supplementary file 12 — Figure EV3 Source Data [file 44318_2025_427_MOESM12_ESM.zip › Figure EV3/EV3B/P21 ctrl spinal cord -TrkA+Homer1+Dapi.tif]

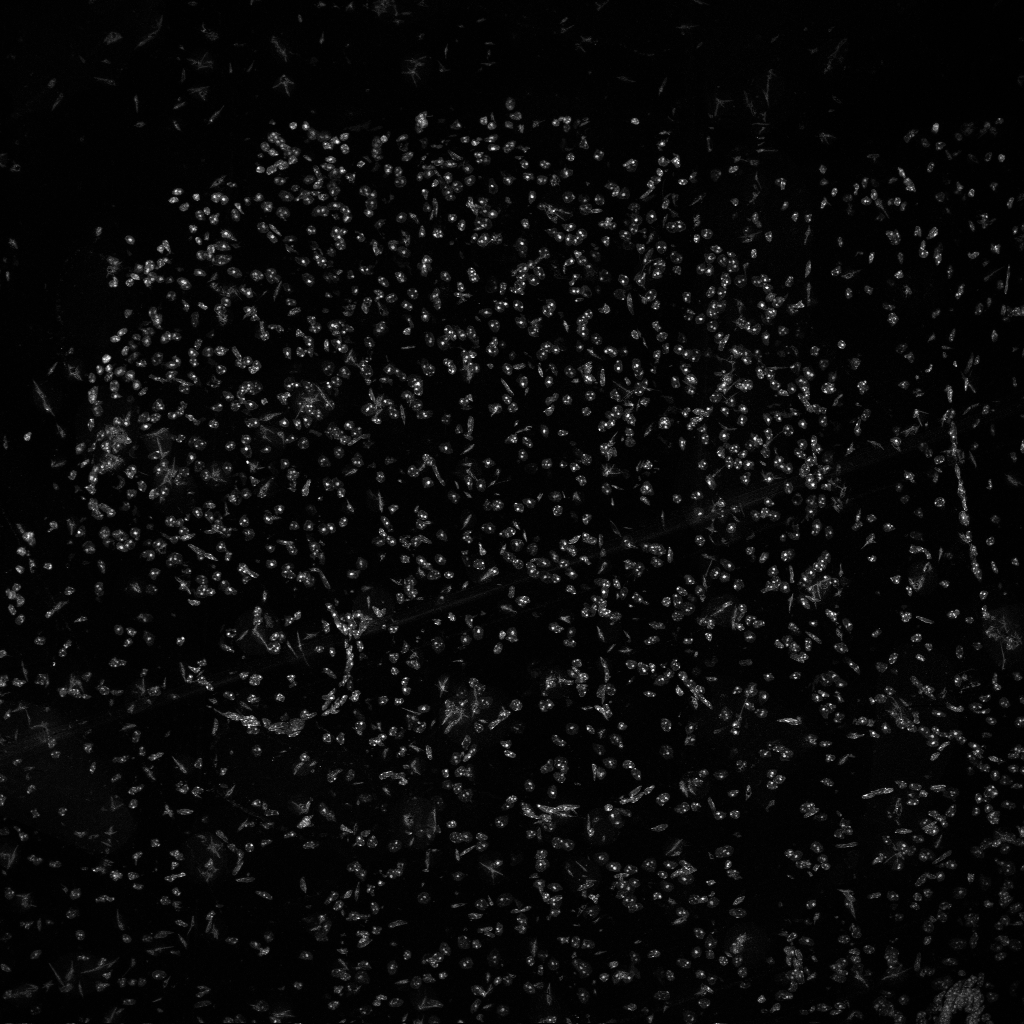

Supplement: Supplementary file 12 — Figure EV3 Source Data [file 44318_2025_427_MOESM12_ESM.zip › Figure EV3/EV3B/P21 SD1OE spinal cord -TrkA+Homer1+Dapi.tif]
