# Supplementary material for: Temporal refinement of Dach1 expression contributes to the development of somatosensory neurons
Source: EMBO J. 2025 Apr 9;44(10):2882–905. doi: 10.1038/s44318-025-00427-y (PMC12084601; doi:10.1038/s44318-025-00427-y)
Supplement: Supplementary file 13 — Expanded View Figures [file 44318_2025_427_MOESM13_ESM.pdf]

Expanded View Figures

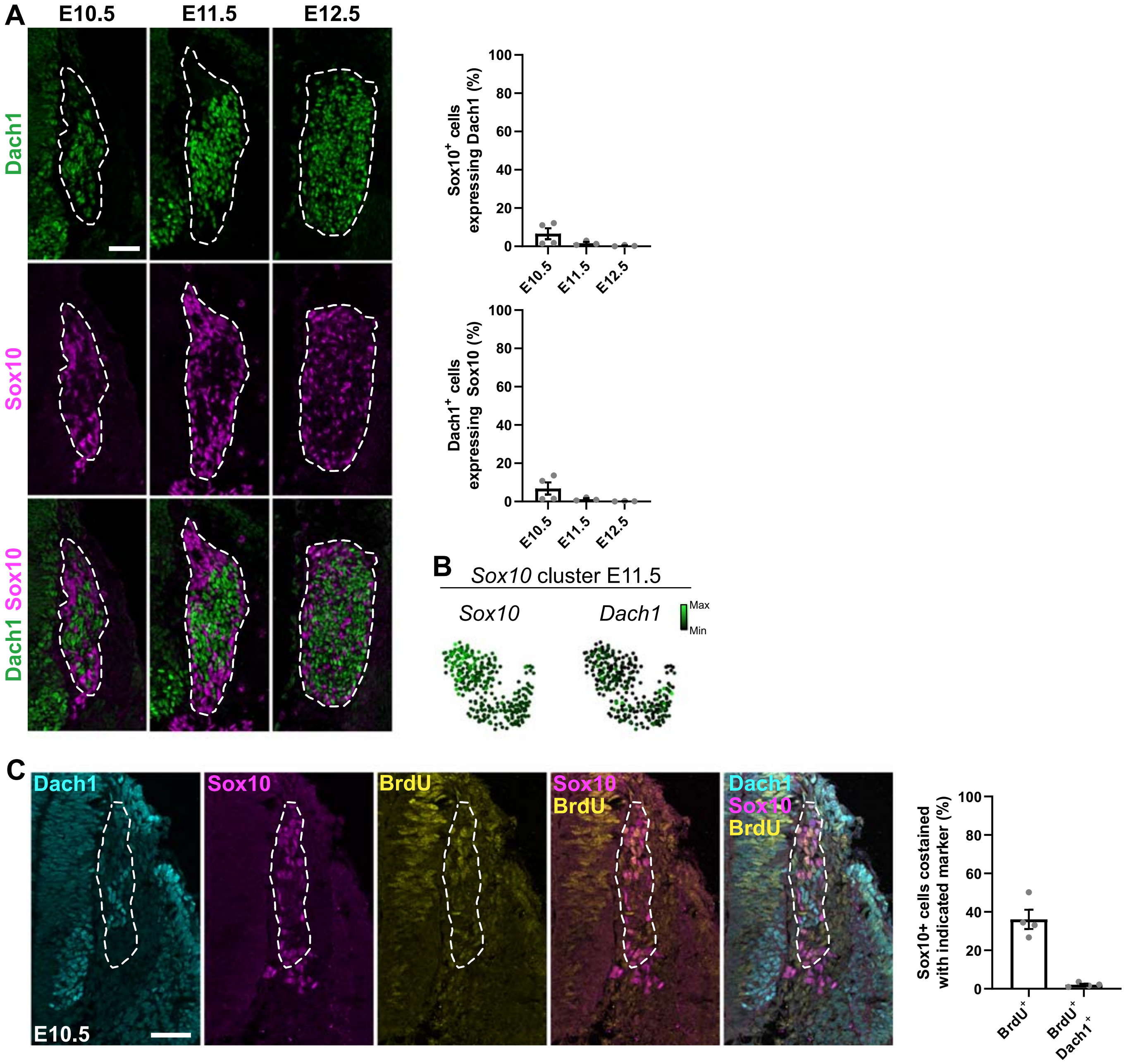

Figure EV1. Characterization of Dach1 expression in Sox10$^+$ progenitors.

(**A**) Left, representative pictures of double immunostainings of Dach1 with the progenitor marker Sox10 performed on thoracic DRG sections of murine embryos at the indicated developmental stages. Right, histogram quantifications of the percentage of Sox10$^+$ cells expressing Dach1 or of Dach1$^+$ cells expressing Sox10 at indicated developmental stages. Graphical data are presented as scatter plots reporting mean ± SEM where each dot represents the mean value of one biological replicate (E10.5, $n = 4$; E11.5, $n = 3$; E12.5, $n = 3$). (**B**) Force-directed layout of single-cell RNA-seq data of E11.5 DRG neurons found to express *Sox10* overlaid with expression levels of indicated genes. Data were obtained from Sharma et al, 2020. (**C**) Left, representative pictures of triple immunostainings of Dach1, Sox10 and BrdU performed on thoracic DRG sections of murine embryos at E10.5 following a 20 min BrdU treatment. Right, histogram quantifications of the percentage of Sox10$^+$ cells expressing Dach1 or of Dach1$^+$ cells expressing Sox10 at indicated developmental stages. Right, histogram quantification showing the percentage of Sox10+ cells co-labelled with BrdU alone or with BrdU and Dach1. Graphical data are presented as a scatter plot reporting mean ± SEM where each dot represents the mean value of one biological replicate ($n = 4$). DRG in pictures are delineated by dashed lines. Scale bars, 50 µm. Source data are available online for this figure.

 © The Author(s)

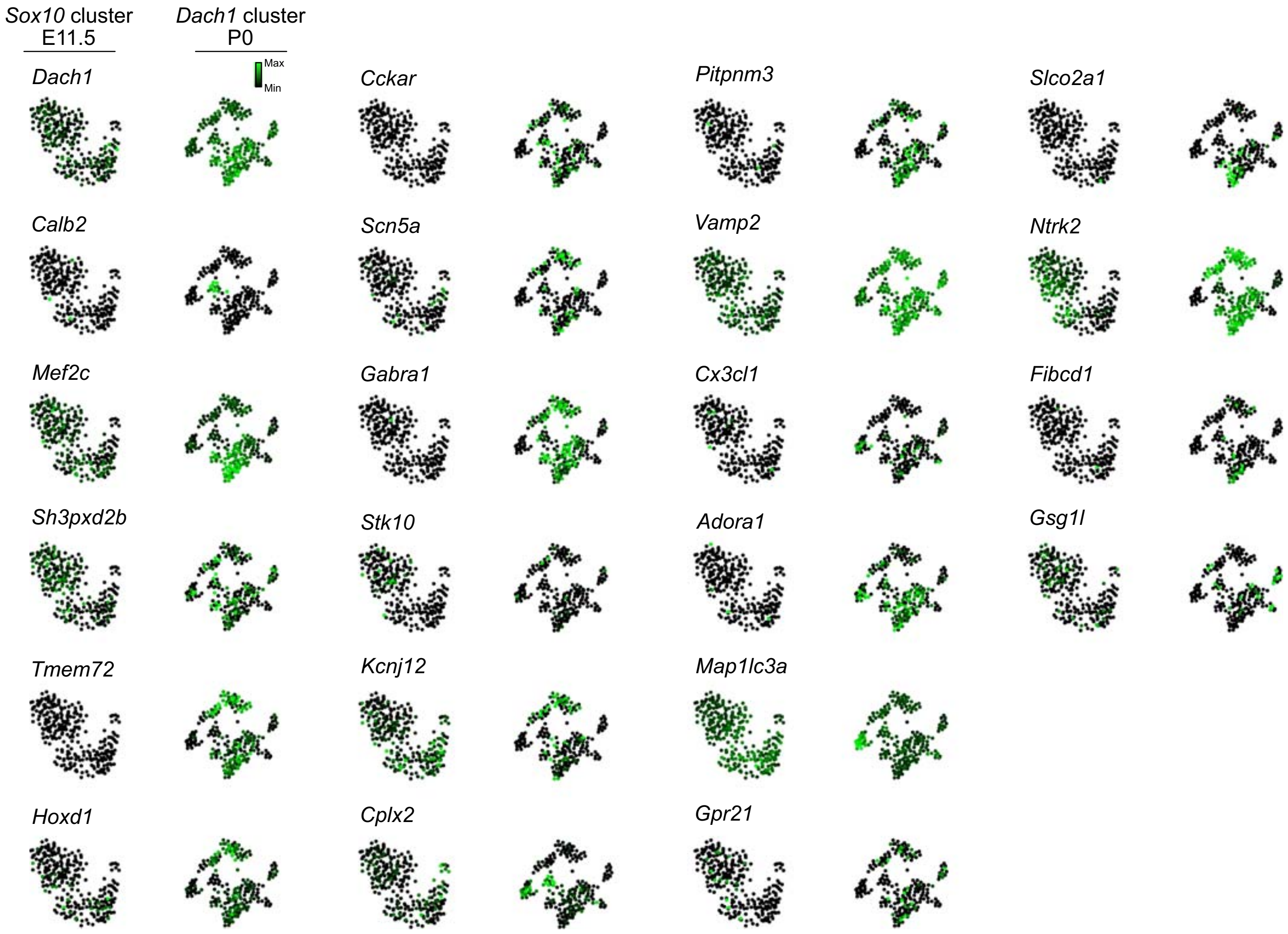

Figure EV2. Downregulated genes identified following Dach1 depletion are preferentially found in postmitotic neurons.

Force-directed layout of single-cell RNA-seq data of E11.5 DRG neurons found to express *Sox10* and P0 DRG neurons found to express *Dach1* overlaid with expression levels of indicated genes. Data were obtained from Sharma et al, 2020.

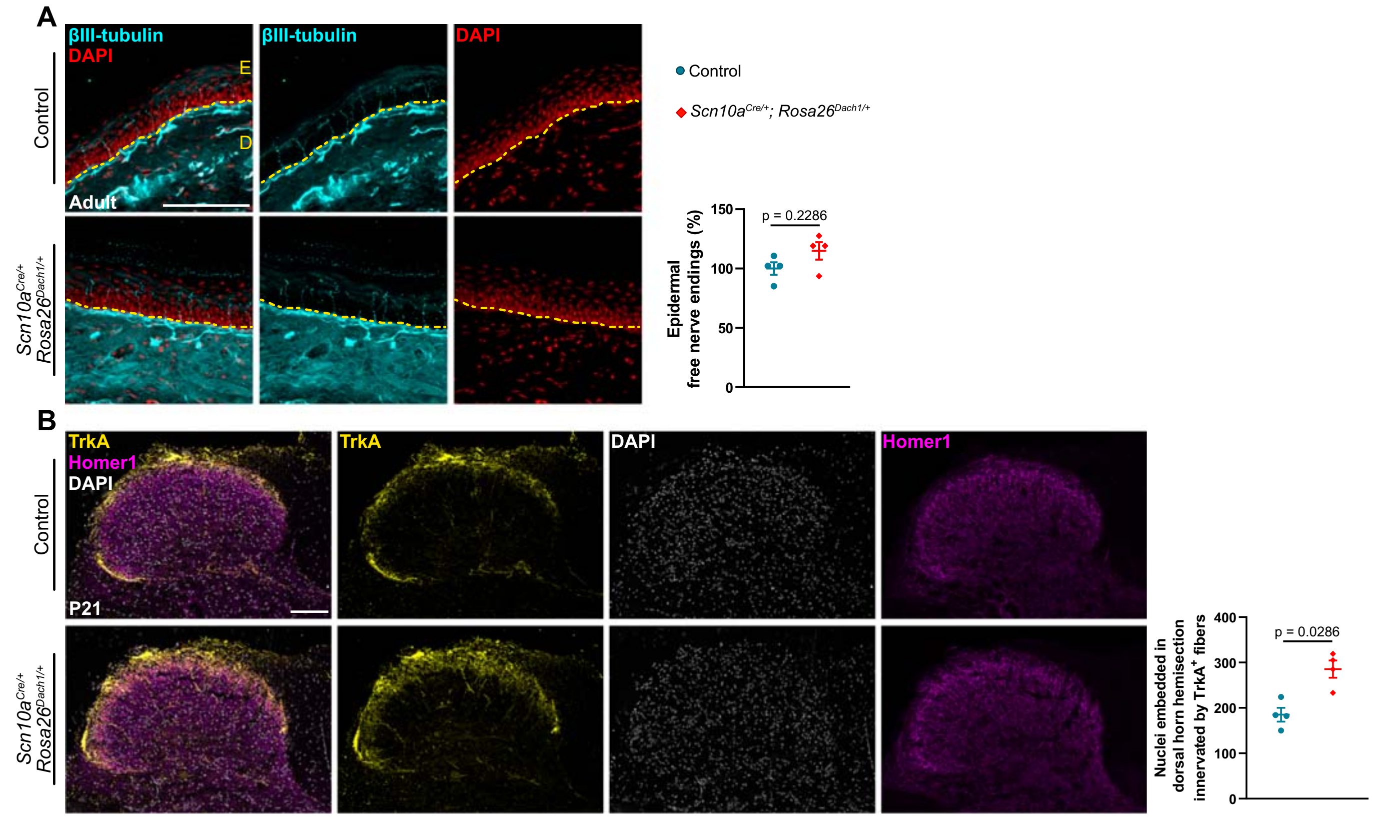

Figure EV3. Characterization of central and peripheral innervation following Dach1 overexpression.

(**A**) Left, representative pictures of immunostainings of βIII-Tubulin labelling nociceptive free nerve endings performed on glabrous hindpaw skin sections of control and *SD1* OE mice counterstained with DAPI (E, Epidermis; D, Dermis). The yellow dashed line delineates the epidermis/dermis boundary. Right, histogram quantification representing the percentage of nociceptive free nerve endings invading the epidermis in *SD1* OE mice ($n = 4$) compared to controls ($n = 4$). Graphical data are presented as a scatter plot reporting mean ± SEM where each dot represents the mean value of one biological replicate. Mann–Whitney test. (**B**) Left, representative pictures of immunostainings of TrkA and Homer1, respectively labelling nociceptive fibres and glutamatergic synapses performed on spinal dorsal horn section of P21 control ($n = 4$) and *SD1* OE ($n = 4$) mice and counterstained with DAPI. Right, histogram quantification representing the mean number of $DAPI^+$ nuclei embedded in the dorsal horn region innervated by $TrkA^+$ nociceptive fibres. Graphical data are presented as a scatter plot reporting mean ± SEM where each dot represents the mean value of one biological replicate. Mann–Whitney test. Scale bars, 100 μm. Source data are available online for this figure.

 © The Author(s)
